# Statistical methods to harmonize electronic health record data across healthcare systems: case study and lessons learned

**DOI:** 10.1093/bioinformatics/btag107

**Published:** 2026-03-02

**Authors:** Xu Shi, Yuqi Zhai, Xianshi Yu, Xiaoou Li, Brian L Hazlehurst, Denis B Nyongesa, Daniel S Sapp, Brian D Williamson, David S Carrell, Luesa Healy, Kara L Cushing-Haugen, Jenna Wong, Shirley V Wang, James S Floyd, Kathleen Shattuck, Samuel McGown, Sarah Alam, José J Hernández-Muñoz, Jie Li, Yong Ma, Danijela Stojanovic, Sudha R Raman, Sharon E Davis, Tianxi Cai, Jennifer C Nelson, Patrick J Heagerty

**Affiliations:** Department of Biostatistics, University of Michigan School of Public Health, Ann Arbor, MI 48109, United States; Department of Biostatistics, University of Michigan School of Public Health, Ann Arbor, MI 48109, United States; Department of Computer Sciences, University of Wisconsin, Madison 53706, United States; School of Statistics, University of Minnesota, Minneapolis, MN 55455, United States; Kaiser Permanente Northwest, Center for Health Research, Portland, OR 97227, United States; Kaiser Permanente Northwest, Center for Health Research, Portland, OR 97227, United States; Kaiser Permanente Northwest, Center for Health Research, Portland, OR 97227, United States; Kaiser Permanente Washington Health Research Institute, Seattle, WA 98101, United States; Kaiser Permanente Washington Health Research Institute, Seattle, WA 98101, United States; Division of Biomedical Health Informatics, Department of Biomedical Informatics and Medical Education, University of Washington School of Medicine, Seattle, WA 98195, United States; Kaiser Permanente Washington Health Research Institute, Seattle, WA 98101, United States; Kaiser Permanente Washington Health Research Institute, Seattle, WA 98101, United States; Department of Population Medicine, Harvard Medical School and Harvard Pilgrim Health Care Institute, Boston, MA 02215, United States; Division of Pharmacoepidemiology and Pharmacoeconomics, Brigham and Women’s Hospital, Boston, MA 02115, United States; Department of Medicine, School of Medicine, University of Washington, Seattle, WA 98195, United States; Department of Epidemiology, School of Public Health, University of Washington, Seattle, WA 98195, United States; Cardiovascular Health Research Unit, University of Washington, Seattle, WA 98195, United States; Department of Population Medicine, Harvard Pilgrim Health Care Institute, Boston, MA 02215, United States; Department of Population Medicine, Harvard Pilgrim Health Care Institute, Boston, MA 02215, United States; Department of Population Medicine, Harvard Pilgrim Health Care Institute, Boston, MA 02215, United States; Office of Surveillance and Epidemiology, Center for Drug Evaluation and Research, US Food and Drug Administration, Silver Spring, MD 20993, United States; Office of Surveillance and Epidemiology, Center for Drug Evaluation and Research, US Food and Drug Administration, Silver Spring, MD 20993, United States; Office of Biostatistics, Office of Translational Sciences, Center for Drug Evaluation and Research, US Food and Drug Administration, Silver Spring, MD 20993, United States; Office of Surveillance and Epidemiology, Center for Drug Evaluation and Research, US Food and Drug Administration, Silver Spring, MD 20993, United States; Department of Population Health Sciences, Duke University School of Medicine, Durham, NC 27710, United States; Department of Biomedical Informatics, Vanderbilt University Medical Center, Nashville, TN 37232, United States; Department of Biostatistics, Harvard T.H. Chan School of Public Health, Boston, MA 02115, United States; Kaiser Permanente Washington Health Research Institute, Seattle, WA 98101, United States; Department of Biostatistics, University of Washington, Seattle, WA 1415 Washington Heights, Ann Arbor, MI 48109, United States

## Abstract

**Motivation:**

Although common data models for electronic health record (EHR) data can facilitate multi-site data organization and querying, the same medical event may still be coded differently between healthcare systems. In this paper, we present statistical methods to identify and mitigate coding discrepancies using summary-level data, and demonstrate these methods using data from two FDA Sentinel data partners: Kaiser Permanente Washington and Kaiser Permanente Northwest.

**Results:**

We first characterize differences in coding patterns, then compute a code mapping matrix to harmonize data between systems. Our findings reveal significant heterogeneity in coded EHR data, even after adopting a common data model with the same coding system, highlighting the importance of data harmonization before downstream analyses. Our study also demonstrates the effectiveness of the data harmonization approaches, which provide a foundational data quality step to promote semantic interoperability, enhance data integration, and improve the integrity of study conclusions.

**Availability and implementation:**

Computation prototypes, including R/Python codes and examples, are included in Section 7, available as supplementary data at *Bioinformatics* online and will be posted on GitHub upon publication.

## 1 Introduction

The U.S. Food and Drug Administration (FDA) launched the Sentinel Initiative to enhance medical product safety through a national system that answers safety questions about approved drugs and vaccines using electronic healthcare data (Robb *et al.* 2012). Sentinel’s Distributed Data Network securely integrates insurance claims data, electronic health records (EHR), and other healthcare databases (Curtis *et al.* 2012) by sharing summary-level data across organizations while protecting patient privacy. This allows the FDA to analyze extensive real-world data to rapidly identify and assess potential safety concerns and make regulatory decisions.

A key challenge for efforts like Sentinel is the heterogeneity in medical coding over time and across healthcare systems. Current practices to harmonize structured EHR data across sites include using a common data model (Klann *et al.* 2019, Haendel *et al.* 2021) and mapping to standardized coding systems, with principles published by organizations such as the World Health Organization and International Organization for Standardization (Pathak *et al.* 2011, Cartwright 2013). Although common data models and mappings between coding systems can facilitate multi-site data organization and querying with standard variable names and formats, the same medical event may still be coded differently within the same system due to varying clinical and administrative coding mechanisms (Zhou *et al.* 2022). For instance, billing incentives may encourage more detailed coding in one system than another. Code substitution, when two similar codes are used interchangeably for the same medical condition, can occur if systems have different technological or organizational factors affecting coding patterns. Coding granularity differences, whereby systems use more or less specific codes for procedures or diagnoses, may also exist. These differences in code usage may be mistaken for true differences in clinical outcomes. Unless detected and harmonized, this heterogeneity can lead to measurement errors, lack of model portability across health systems, and ultimately downstream bias in study conclusions.

In this article, we study approaches to harmonize structured EHR data by detecting and reducing heterogeneity in coding differences between two healthcare systems. We focus on harmonization after datasets have been mapped to the same common data model and coding system. That is, we study how the same set of codes can be used differently across healthcare systems and how to mitigate such differences. Developing a formal approach to reduce coding heterogeneity is critical but challenging due to high data dimensionality and data-sharing constraints to protect patient privacy in federated systems like Sentinel. Manual inspection is often ad hoc and not scalable for all codes of interest. Moreover, validation of harmonization results is difficult without a known truth and requires multidisciplinary expertise from clinicians, informaticians, statisticians, and others.

To tackle these challenges, we apply and evaluate data-driven, privacy-enhancing statistical methods to describe and mitigate coding differences between healthcare systems. We illustrate these methods using data from two Sentinel healthcare systems: Kaiser Permanente Washington (KPWA) and Kaiser Permanente Northwest (KPNW). We use summary-level data to characterize heterogeneity in coding patterns and then perform harmonization of medical codes. We validate our results using novel statistical methods.

**Figure 1:**
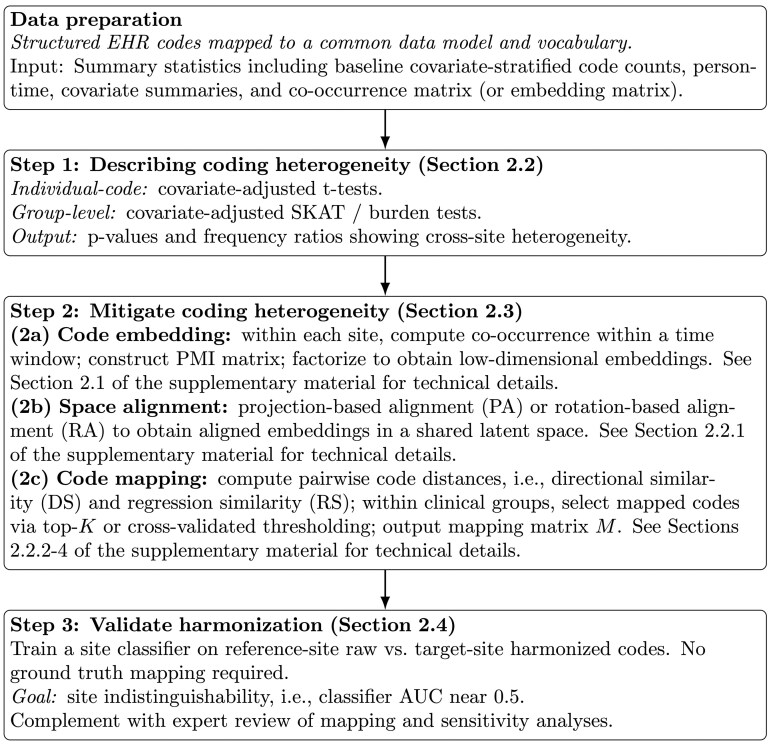
Workflow schematic for describing and mitigating cross-site coding heterogeneity in federated EHR networks using summary-level information.

The primary contribution of this work is the development of an end-to-end framework for quality checking and harmonizing structured EHR data in federated data networks while preserving patient privacy. While group-wise association tests and embedding alignment and mapping build on prior literature, their integration into a unified pipeline that operates entirely on summary-level information distinguishes this approach from existing methods. In addition, the proposed framework incorporates an empirical validation strategy that does not rely on the availability of ground-truth mappings. Figure 1 presents a schematic of the end-to-end harmonization workflow, including heterogeneity detection, code embedding, space alignment, code mapping, and validation.

## 2 Materials and methods

### 2.1 Data source

We assembled data from 2011 to 2022 to form a dynamic cohort of diabetes patients at KPWA and KPNW, two integrated healthcare systems in Washington and Oregon, respectively. Data from both sites were mapped to the Sentinel Common Data Model with standard coding systems, and we obtained information on enrollment, ICD-9, ICD-10, CPT, LOINC, HCPCS, local health system-specific codes, and other code types (Xu *et al.* 2015, Garza *et al.* 2016). Patients newly meeting eligibility criteria (detailed below) on January 1 of each year (referred to as the eligibility year) entered the study cohort. The following year served as the study year, during which diagnosis, procedures, and laboratory data were captured. Existing cohort members who no longer met eligibility criteria were censored. Eligibility criteria included: age 50+, had been enrolled in KPWA or KPNW for at least 9 months of the eligibility year, had a diagnosis of diabetes in the eligibility year, and ≥1 month of observed data during the study year. We restricted the cohort to patients aged 50 years and older to focus on a population with a higher prevalence of chronic comorbidities and richer coding patterns, thereby improving power and comparability across sites. The cohort includes patients with any type of diabetes, including type I, type II, and secondary diabetes, identified using diagnosis codes, laboratory measurements, and medication use, consistent with Sentinel definitions. See Section 1, available as supplementary data at *Bioinformatics* online for more details.

For each cohort member, we defined baseline characteristics from the first qualifying eligibility year: age, sex, insulin use, Elixhauser comorbidity index (Austin *et al.* 2015) and its components (e.g. cardiovascular disease, chronic kidney disease, depression), BMI, HbA1c, and healthcare utilization measures (e.g. number of hospitalizations, visits, and medications).

### 2.2 Describing coding heterogeneity

To assess coding heterogeneity between KPWA and KPNW, we compared the use of both individual and groups of codes across all patients and study years. We studied ICD, CPT, LOINC, revenue, HCPCS, local, and other codes. We computed a frequency ratio for each code, defined as the ratio of coding frequency (total number of code endorsements divided by total person-time) at KPWA to KPNW. A ratio substantially higher or lower than one indicates stronger or weaker endorsement at KPWA compared to KPNW. We also compared coding between consecutive study years to detect heterogeneity over time within each KP system.

For code-level comparisons, we used a weighted two-sample t-test, adjusting for person-time and baseline characteristics. For group-level comparisons, we aggregated ICD-9, ICD-10, and CPT codes into coarser clinical categories, using standard ICD phecode groups from the phenome-wide association study catalog and the Clinical Classification Software for CPT grouping (Denny *et al.* 2013, Wei *et al.* 2017). We applied the sequence kernel association test (SKAT) and burden test, association tests initially proposed for genomic research (Lee *et al.* 2014, Shi *et al.* 2017). All tests were conservatively corrected for multiple comparisons using Bonferroni correction and adjusted for baseline age, sex, insulin use, and Elixhauser index.

Specifically, we fit a logistic regression model in which the outcome is the healthcare system indicator, and the predictors include grouped medical code endorsements and patient characteristics. The null hypothesis for both the SKAT and burden test is that, after adjustment, the grouped codes are not associated with the healthcare system, indicating no residual coding heterogeneity. While the burden test assumes effects in a common direction, SKAT allows heterogeneous directions and is particularly sensitive to code substitution within a group. We further enhanced privacy in these methods for distributed data networks like Sentinel by creating versions that require only summary data sharing but produce the same results as using individual-level data.

### 2.3 Mitigating coding heterogeneity

Inspired by language translation in the natural language processing (NLP) literature (Mikolov *et al.* 2013), we harmonize data by creating a mapping of medical codes between KPWA and KPNW (the reference site) using a three-step process:

1. Code embedding: Generate site-specific code embeddings, which are analogous to word embeddings in NLP, using a method proposed in Beam *et al.* (2020).
2. Space alignment: align two sets of code embeddings (each from a site) into a common “language” space, such that one can measure and compare distances across sites.
3. Code mapping: find the nearest neighbor(s) of each KPWA code from KPNW codes within the same clinical category.

Word embeddings are vector representations of words, where words with similar meanings have similar directions in the vector space. Based on the analogy between words in human language and codes in healthcare data, one can generate code embeddings using the same strategy by analyzing the co-occurrence patterns of codes in patients’ medical records (Beam *et al.* 2020). See Section 2.1, available as supplementary data at *Bioinformatics* online for more details.

We clarify related terminology with distinct meanings as follows. Throughout this paper, alignment refers to transforming site-specific embedding spaces into a common latent coordinate system; mapping refers to the learned correspondence between codes across systems; and harmonization refers to the overall process that integrates code embedding, embedding space alignment, and code mapping to improve cross-site comparability.

We considered two methods for space alignment: projection-based alignment (PA) and rotation-based alignment (RA). Briefly, projection-based alignment uses regression techniques to predict codes across sites, while rotation-based alignment includes an additional constraint that the predictions remain standardized or have a common length (Xing *et al.* 2015, Shi *et al.* 2021). See Section 2.2.1, available as supplementary data at *Bioinformatics* online for more details.

We define two versions of similarity for code mapping: directional similarity (DS) and regression similarity (RS). Directional similarity, i.e. the cosine similarity, is commonly used in NLP. It essentially produces an unadjusted association between a pair of codes, while the regression similarity corresponds to an adjusted association. With a given similarity measure, for each code in KPWA, we select either the first K codes with the highest similarities (top-K matching) or all codes whose corresponding similarities are higher than a data-driven threshold selected by cross-validation. See Section 2.2.2, available as supplementary data at *Bioinformatics* online for more details.

We denote our main analysis as RADS, which consisted of rotation-based embedding alignment followed by computing directional similarity of each pair of codes between KPWA and KPNW for mapping. RADS was selected for the primary analysis because it is based on directional similarity in the embedding space and is therefore robust to scale differences across site-specific embeddings, which can arise from differences in code frequency distributions and co-occurrence structure (Xing *et al.* 2015, Shi *et al.* 2021). Sensitivity analysis included: (i) PADS (projection-based alignment followed by directional similarity); (ii) RARS (rotation-based alignment followed by regression similarity); and (iii) refining the similarity measure such that it matches individual code frequency between sites, which may improve code mapping (Section 4, available as supplementary data at *Bioinformatics* online).

### 2.4 Validation method

To validate the learned code mapping, we evaluated its ability to harmonize data between KPWA and KPNW by asking: “Can a site classifier be confused after harmonization?” Specifically, we trained a logistic regression model using cataract-related codes as predictors and the site indicator as the outcome. Predictors were derived from raw KPNW data and harmonized KPWA data. A successful mapping should make the harmonized KPWA data statistically indistinguishable from KPNW data, resulting in a model unable to accurately predict the site of origin. Effective harmonization is therefore reflected by poor classification performance, with an AUC approaching 0.5. For comparison, we also computed the AUC before harmonization, using overlapping codes between KPNW and KPWA as predictors.

This evaluation approach is motivated by domain confusion principles and provides an empirical strategy for assessing mapping quality in the absence of ground-truth code mappings, which are infeasible to establish comprehensively across all medical codes. To complement this quantitative evaluation, clinical experts reviewed and validated code comparison and mapping results for selected disease conditions, including myocardial infarction, heart failure, diabetes, hypertension, and cataracts.

All technical details are presented in Sections 2–4, available as supplementary data at *Bioinformatics* online.

## 3 Results

### 3.1 Study population

Our final cohort included 74 475 patients at KPWA and 64 231 at KPNW. Table 1 presents patient baseline characteristics overall and by health system. Patients had a mean follow-up time of 4.5 years, age of 62.8 years, an Elixhauser comorbidity index of 3.6, and HbA1c of 7.2. KPWA and KPNW patients were similar in both demographics and prevalence of chronic conditions. KPWA had more missing data, including for race, BMI, and HbA1c. This is because, for example, the KPWA EHR does not capture HbA1c if the lab test was ordered at an outside contracting clinic.

**Table 1:** Baseline patient characteristics by health system and overall.

	KPWA (*N* = 74 475)	KPNW (*N* = 64 231)	All (*N* = 138 706)
Age, mean (SD)	62.8 (9.95)	62.8 (9.91)	62.8 (9.93)
Female	36 631 (49%)	31 461 (49%)	68 092 (49%)
Insulin use	17 184 (23%)	12 207 (19%)	29 391 (21%)
Race			
Unknown	20 570 (28%)	4168 (6%)	24 738 (18%)
American Indian or Alaska Native	1300 (2%)	925 (1%)	2225 (2%)
Asian	5776 (8%)	3661 (6%)	9437 (7%)
Black or African American	3328 (4%)	2495 (4%)	5823 (4%)
Native Hawaiian or Other Pacific Islander	773 (1%)	855 (1%)	1628 (1%)
White	42 728 (57%)	52 127 (81%)	94 855 (68%)
Hispanic			
No	52 534 (71%)	58 547 (91%)	111 081 (80%)
Unknown	19 107 (26%)	1407 (2%)	20 514 (15%)
Yes	2834 (4%)	4277 (7%)	7111 (5%)
Number of hospitalizations			
Mean (SD)	0.190 (0.598)	0.198 (0.622)	0.194 (0.609)
Median [Min, Max]	0 [0, 13.0]	0 [0, 15.0]	0 [0, 15.0]
Number of ambulatory visits			
Mean (SD)	13.7 (15.9)	11.1 (15.0)	12.5 (15.6)
Median [Min, Max]	9.00 [0, 365]	7.00 [0, 366]	8.00 [0, 366]
BMI			
Mean (SD)	33.1 (7.44)	33.9 (7.71)	33.5 (7.61)
Missing	38 188 (51%)	13 946 (22%)	52 134 (38%)
HbA1c			
Mean (SD)	7.22 (1.51)	7.17 (1.43)	7.19 (1.46)
Missing	31 151 (42%)	2916 (5%)	34 067 (25%)
Number of medications, mean (SD)	10.5 (6.67)	11.4 (7.51)	10.9 (7.09)
Use of other diabetes medications (i.e. other than insulin)	45 996 (62%)	38 051 (59%)	84 047 (61%)
Cardiovascular disease	11 781 (16%)	9425 (15%)	21 206 (15%)
Chronic kidney disease	10 772 (14%)	9833 (15%)	20 605 (15%)
Congestive heart failure	5167 (7%)	4680 (7%)	9847 (7%)
Depression	12 689 (17%)	10 768 (17%)	23 457 (17%)
Elixhauser comorbidity score			
Mean (SD)	3.59 (2.34)	3.52 (2.28)	3.56 (2.31)
Missing	339 (0.5%)	36 (0.1%)	375 (0.3%)

We identified 65 935 unique medical codes recorded on our cohort members during the study years, including 11 950 ICD-9, 36 538 ICD-10, 7749 CPT, 59 LOINC, 3174 HCPCS, 2100 local, 3654 other, and 711 revenue codes. KPWA endorsed 53 004 unique codes used 57 938 353 times (mean 664 usages per patient, 1093 per code). Of these, 16% were ICD-9, 30% ICD-10, and 35% CPT codes. KPNW endorsed 49 761 unique codes used 55 876 254 times (mean 641 usages per patient, 1123 per code). Among these, 13% were ICD-9, 27% ICD-10, and 34% CPT codes.

### 3.2 Describing heterogeneity in coding between KPWA and KPNW

**Figure 2:**
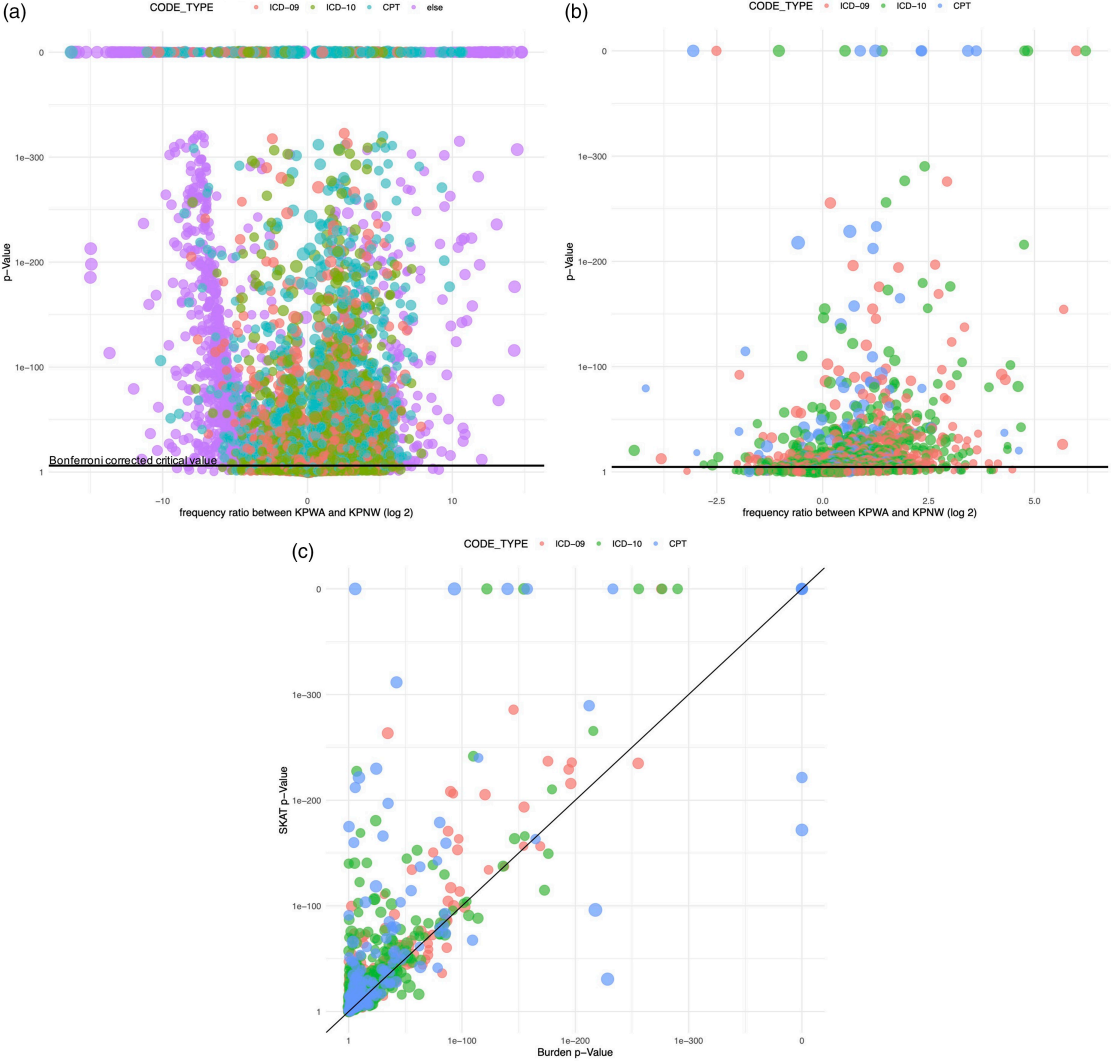
Results of individual- and group-level code comparison between KPWA and KPNW. Panel (a) presents adjusted *P*-values (*y*-axis) from t-test and frequency ratio comparing KPWA to KPNW (*x*-axis); Panel (b) presents adjusted *P*-values (*y*-axis) from burden test and frequency ratio comparing KPWA to KPNW (*x*-axis). A frequency ratio greater than one indicates more utilization per person-year at KPWA, and vice versa. Panel (c) plots adjusted *P*-values from SKAT against adjusted *P*-values from burden test. Each point corresponds to a specific code and codes are color coded by code type (e.g. ICD-9, ICD-10, CPT, etc.).

Using the adjusted and Bonferroni corrected two-sample t-test, we detected significant coding differences between KPWA and KPNW for 13% (8348/65 935) of the unique codes. The differences were substantial: 6% (4086) of codes had a frequency ratio >5, and 4% (2744) had a frequency ratio <1/5, indicating a five-fold difference between health systems. Figure 2a shows *P*-values from the t-test plotted against the (scaled) frequency ratio, revealing that KPNW uses more local and “other” codes. Because not all local codes map to common ontologies, this observation highlights the need for data-driven code mapping.

In adjusted Bonferroni-corrected group-level comparisons, the burden test and SKAT detected significant coding differences for 33% (828/2523) and 39% (994/2523) of phecode groups, respectively. Over 125 code groups had five-fold or more differences. Figure 2b shows burden test *P*-values against the (scaled) frequency ratio, indicating more unique code groups used at KPWA. Note that group-level tests were performed for ICD and CPT code groups only, and the higher frequency ratio of these groups at KPWA corroborates Fig. 2a’s finding that KPNW uses non-ICD/CPT codes more. These significant differences underscore the importance of code mapping.

Figure 2c plots the SKAT versus burden test *P*-values and shows that SKAT tended to return smaller *P*-values, indicating more significant results. This is because SKAT allows for individual coding differences within a group to be in different directions, whereas the burden test which assumes that differences are in the same direction. Thus, SKAT can better detect additional types of heterogeneity, such as code substitution. For example, SKAT detected significant heterogeneity (*P* < 1 × 10^−20^) in the ICD-10 phecode group 366 (cataract), despite a frequency ratio of 1.03, indicating similar group-level counts but differing individual code usage. Table 2 explores individual code usage within the cataract group and lists the frequency and frequency ratio of individual codes. Codes for conditions characterized as unspecified were more frequently coded at KPNW, while KPWA used more specific codes.

**Table 2:** Differences in non-rare (frequency ≥ 10) individual ICD-10 codes within the cataract group (phecode 366): frequency of usage, frequency ratio adjusting for patient follow-up time (person-years), and adjusted *P*-value from t-test

Code	Description	Frequency			Adjusted
		KPWA	KPNW	Ratio^a^	*P*-value
E08.36	Diabetes mellitus due to underlying condition with diabetic cataract	23	0	3.10	6.12 × 10^−03^
E10.36	Type 1 diabetes mellitus with diabetic cataract	92	117	0.75	6.06 × 10^−02^
E11.36	Type 2 diabetes mellitus with diabetic cataract	3065	2996	0.96	5.88 × 10^−01^
H26.40	Unspecified secondary cataract	561	1144	0.46	1.62 × 10^−39^
H26.411	Soemmering’s ring, right eye	11	1	1.79	1.26 × 10^−01^
H26.491	Other secondary cataract, right eye	3044	771	3.67	<10^−100^
H26.492	Other secondary cataract, left eye	3129	741	3.93	<10^−100^
H26.493	Other secondary cataract, bilateral	3952	636	5.76	<10^−100^
H26.499	Other secondary cataract, unspecified eye	70	0	7.51	1.55 × 10^−14^
H26.8	Other specified cataract	526	1323	0.38	5.22 × 10^−27^
H26.9	Unspecified cataract	16704	15786	0.99	8.53 × 10^−01^
H59.021	Cataract (lens) fragments in eye following cataract surgery, right eye	47	14	2.23	1.31 × 10^−01^
H59.022	Cataract (lens) fragments in eye following cataract surgery, left eye	78	10	4.13	1.03 × 10^−02^
H59.029	Cataract (lens) fragments in eye following cataract surgery, unspecified eye	1	72	0.13	1.15 × 10^−06^
Z96.1	Presence of intraocular lens	35888	44526	0.76	1.31 × 10^−79^
Z98.41	Cataract extraction status, right eye	3950	199	17.79	<10^−100^
Z98.42	Cataract extraction status, left eye	3723	195	17.10	<10^−100^
Z98.49	Cataract extraction status, unspecified eye	622	112	4.87	1.15 × 10^−33^

a Frequency ratio is defined as (frequency in KPWA + 10)/patient years in KPWA divided by (frequency in KPNW + 10)/patient years in KPNW.

We also compared group-level differences across consecutive study years within each system, focusing on ICD-10 codes (see Table 2, available as supplementary data at *Bioinformatics* online). A higher proportion of differences were found in 2019 vs. 2020 and 2016 vs. 2017. Differences between 2019 and 2020 may be explained by patient healthcare utilization changes and organizational upheaval at healthcare systems due to COVID-19, while those between 2016 and 2017 may reflect the delayed impact of the ICD-9 to ICD-10 transition in October 2015.

### 3.3 Mitigating heterogeneity in coding between KPWA and KPNW

In this section, we focus on the cataract phecode group to investigate if data-driven code mapping can address the code substitution identified in Section 3.2 by linking specific cataract codes used at KPWA with corresponding non-specific codes at KPNW. This analysis included 80 379 eligible patients at KPWA and 68 484 at KPNW, as it does not require patients to have ≥9 total months of follow-up during a qualifying study year.

We generated embeddings for non-rare codes (frequency ≥10), including 6400 ICD-9, 14 537 ICD-10, and 4253 CPT codes from KPWA, and 5545 ICD-9, 11 142 ICD-10, and 3334 CPT codes from KPNW. Before space alignment, the average within-group directional similarity was 0.046, which increased to 0.270 with rotation-based alignment and to 0.336 with projection-based alignment.

Within the cataract group, there were 17 and 15 non-rare codes at KPWA and KPNW, respectively. Figure 2a, available as supplementary data at *Bioinformatics* online presents a matrix of directional similarities (cosine values) of all possible pairs of these codes, while Fig. 2b, available as supplementary data at *Bioinformatics* online presents the refined matrix after incorporating individual code frequency.

**Figure 3:**
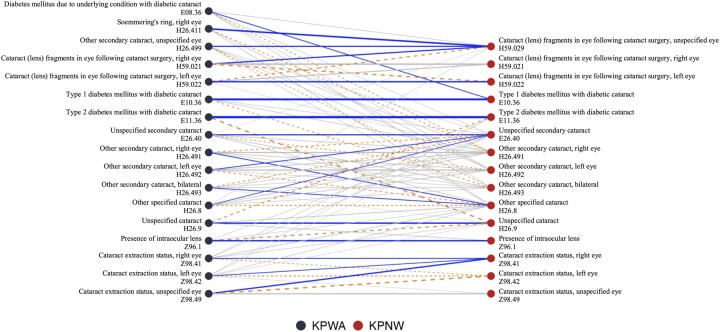
Results of mapping from KPWA to KPNW from the main analysis based on the RADS method. 17 KPWA codes are listed on the left and 15 KPNW codes are listed on the right. Gray lines represent links with similarities larger than the data-driven thresholding (which is equal to 0.13), blue lines correspond to top 1 mapping, i.e. for each source code at KPWA, a blue line links it to the code at KPNW with the largest similarities as this source code, and orange dashed lines are based on selecting the second largest similarities. Line width indicates the magnitude of the similarity between the pair of linked codes.

Figure 3 presents code mapping results from KPWA to KPNW using the RADS method. Thicker lines indicate higher similarity, with line color and type denoting mapping methods. Most lines are horizontal, indicating similar coding patterns between systems. More specific codes at KPWA were collapsed and mapped to the relevant less specific codes at KPNW. For example, “cataract (lens) fragments in eye following cataract surgery, right eye” at KPWA were mapped to “cataract (lens) fragments in eye following cataract surgery, unspecified eye” at KPNW.

We also noticed that three codes for “cataract extraction status” at KPWA (left, right, and unspecified eye) were all mapped to the right eye at KPNW based on top 1 mapping. In the similarity matrix, these three codes at KPWA showed high similarity to both the right and left eye codes at KPNW, but not to the unspecified eye code due to its less frequent use at KPNW. This example highlights the need to explore multiple ways to select a map, such as top 1, top 2, and thresholding.

For validation, we computed the AUC of classifying a patient’s health system site using their harmonized codes. Ideally, harmonization should yield an AUC of 0.5 (no better than chance) because the coding patterns from each health system site should be indistinguishable. The cross-validated AUC was 0.715 (0.709, 0.720) before harmonization and 0.586 (0.580, 0.592) after harmonization using the RADS method, much closer to the ideal value. This indicates that data from both systems were now well-aligned after harmonization. Using alternative alignment methods yielded similar reductions in site-classification performance, with cross-validated AUCs of 0.556 (95% CI: 0.550, 0.562) for PADS and 0.533 (0.527, 0.539) for RARS, which indicates that the conclusion of improved harmonization is robust to alignment choices.

Clinicians and informaticians on the team reviewed the code comparison and mapping results, focusing on specific disease conditions, including myocardial infarction, heart failure, diabetes, hypertension, and cataracts. For instance, to understand why KPWA uses more “specified” codes in the cataract group, we found that external providers frequently use specific codes for billing purposes, whereas such specificity is unnecessary in integrated care settings. KPWA provides insurance coverage across a much larger geographic region than KPNW and includes many providers from networks that are “external” to the organization. We confirmed that KPWA has a higher proportion of coding events that are generated by external providers: 19.76% of Optometry codes and 46.82% of Ophthalmology codes at KPWA are generated externally, compared to only 2.57% and 0.49% at KPNW, respectively.

Sensitivity analyses using PADS and RARS methods produced similar results (Fig. 3, available as supplementary data at *Bioinformatics* online), indicating robustness to different space alignment and similarity measurement methods.

## 4 Discussion

In this 2011–2022 study of diabetic patients aged 50 and older, we applied novel data-driven statistical methods to describe and mitigate coding differences between two health system sites: KPWA and KPNW. We showed that coded EHR data can be highly heterogeneous, with five-fold differences in many coding frequencies, even across two sites with similar health plans and patient populations, and even at an aggregated group level. We detected more local codes at KPNW, more granular codes at KPWA, and code substitution within the cataract phecode group. Our mapping methods successfully increased coding similarity between KPWA and KPNW. For phecode groups like cataract, our mapping corrected code substitution by collapsing and mapping more specific codes at KPWA to less specific ones at KPNW. These results highlight the importance of addressing heterogeneity before conducting multi-site analysis or model transfer to ensure consistent assessment of key study measures and model inputs. Our approach can provide a foundational data quality step to promote semantic interoperability, enhance data integration across healthcare systems, and improve the integrity of study conclusions.

Our statistical code mapping approach for structured EHR data is inspired by existing language translation methods. However, direct application of word embedding algorithms from NLP may not be suitable for EHR coding patterns. Word embeddings are often obtained by decomposing a shifted positive pointwise mutual information (SPPMI) matrix, which zeroes out rows for extremely common words or stop words that do not provide semantic information (such as “and” or “the”). This principle, while useful in NLP, is unsuitable for EHR data where high-frequency medical codes may designate common disease conditions that are important outcomes or predictors. In this study, 15 (0.06%) of 27 167 non-rare codes in KPWA and 34 (0.14%) of 23 767 in KPNW were zeroed out based on the SPPMI matrix, including important codes like CPT code 83036 [Hemoglobin; glycosylated (A1c)]. Therefore, we recommend using the PMI matrix for generating code embeddings instead of traditional word embedding methods.

Our methods address key challenges in EHR data harmonization. They are data-driven and objective. They do not simply collapse individual-level codes into phenotypes or general concepts, which is often used to bypass coding differences but may not suffice for all research objectives, particularly for prognostic models that use individual codes. Our mapping approach is also computationally efficient, feasible in distributed data settings where only population-level summary data need to be shared, and is comprehensive, in that it could be scaled to evaluate the entire structured EHR. It can also be applied to codes at different granularity levels, such as rolled-up codes or code groups. These technical and practical strengths offer promise that such procedures could be automated and integrated into data quality assurance routines for data networks like Sentinel, either comprehensively or for targeted priority codes. Doing so would meet a critical goal noted by the FDA (Burns *et al.* 2022, Girman *et al.* 2022) to better describe variation in medical coding among health system sites and over time to ensure consistency.

A key challenge is to distinguish discrepancies in coding practices from genuine differences in patient populations and healthcare practices. In our study, we implemented several strategies to mitigate potential differences in patients and healthcare practices (rather than coding practices). First, we selected similar health systems (two regions of KP in close geographical proximity) and restricted analyses to a disease-specific cohort with similar patient characteristics and healthcare practices. Table 1 confirms the comparability of our diabetic cohorts. Second, we adjusted for patient characteristics in t-test, SKAT, and burden test for code comparisons, including baseline age, sex, insulin use, and Elixhauser index, and performed space alignment prior to code mapping. These design- and model-based adjustments are intended to attenuate population-related differences; however, such differences may only be partially accounted for. For example, variations in healthcare practices may exist even within KP, such as specialty clinics at one site that may not be available at another site, which may force a mapping to less specific codes. As a result, code mapping procedures may reduce both site-specific coding differences and, in some cases, clinically meaningful differences. Similarly, our validation strategy based on site-classification performance should be interpreted as a pragmatic diagnostic of residual site-specific coding signal, as it operates solely on code-based representations. Residual site distinguishability after harmonization may therefore reflect coding heterogeneity, population differences, or both. Future research should focus on developing methods to detect and account for these residual differences to enhance harmonization validity. One potential direction is to develop privacy-preserving entropy-weighting approaches that balance factors likely to contaminate coding differences across sites, such as patient characteristics, referral patterns, and care-delivery structures. This approach requires sharing only aggregated moments of these factors to estimate entropy-balancing weights, so that the weighted distribution of patient characteristics at one site matches that of another site with respect to a prespecified set of balance functions. A key limitation is the assumption that all relevant factors are fully captured and available within the healthcare systems. An alternative strategy that can accommodate unmeasured population differences is falsification, which involves examining a set of codes known to be consistently used across sites. Null results in code comparisons and mappings would be expected; any significant differences detected would likely reflect residual differences unrelated to coding practices.

Our mapping method application lays the groundwork for future studies. First, validation methods could be improved. Our proposed validation approach can be further leveraged to select tuning parameters for code embedding, space alignment, and code mapping thresholds, all of which could improve mapping results. One could also assess the coefficients of codes in the site classification model to investigate which codes remain imbalanced between sites. Alternatively, one could reconduct SKAT, burden, and t-test analyses on harmonized codes to assess whether coding differences are reduced by code mapping. Second, one could extend our harmonization methods to multiple sites: embedding construction and alignment can be performed for each site, followed by mapping to a selected reference site or a consensus latent space; validation can be extended using multiclass or one-versus-rest site classifiers. Identifying a reference site can be challenging and subjective. A suitable reference site may be characterized by the availability of outcome labels, richer and more complete data, stable coding practices, high-integrity coding documentation, or relevance to downstream analyses. Importantly, reference-site selection can substantially influence harmonization quality and downstream inference. For example, a reference site with poor data completeness or coding quality may induce noisy or unstable mappings, while a reference site misaligned with the scientific objective of interest may limit the generalizability of downstream analysis. While Sentinel has implemented a distributed data network infrastructure and a standardized data model to accommodate diverse operational and administrative structures across healthcare systems, conducting multi-site comparisons may still present challenges due to different coding workflows, care delivery models, health information technology systems, reimbursement models, and so on. Greater heterogeneity may arise across substantially different Sentinel data partners (e.g. KP versus non-KP systems), and such settings may require stronger adjustment, additional diagnostics, or more conservative interpretation of mappings and validation results. Third, our study focused on one disease cohort. Cataracts were selected because they exhibit clear differences in coding granularity and substitution across sites while remaining clinically well-defined. For conditions with lower prevalence or greater clinical heterogeneity, embedding stability and mapping performance may vary due to sparser co-occurrence patterns and more diffuse coding practices. It is thus important to investigate the generalizability and robustness of our methods across different medical conditions with varying degrees of complexity, different coding practices, or less comprehensive data. Fourth, our methods can be potentially implemented in the Sentinel Routine Querying System, a SAS tool that facilitates rapid execution of standardized queries on the Sentinel common data model, to conduct periodic recalibration as clinical guidelines and coding practices evolve over time. Future studies should evaluate whether harmonization effects vary over time. Fifth, our current approach maps KPWA to KPNW, possibly leaving some KPNW codes unmapped. In practice, bi-directional mapping may be preferable and warrants future research. Lastly, integrating unstructured clinical notes with structured data could improve overall data integration. Sixth, differences in missingness of baseline covariates across sites (e.g. BMI or HbA1c) may reflect variation in data capture mechanisms and can affect covariate adjustment, thereby influencing harmonization results. Because code embeddings are learned from code co-occurrence patterns rather than from raw covariate values, the direct impact of covariate missingness on embedding construction is partially mitigated. Nevertheless, differential missingness may still indirectly affect harmonization and remains an important consideration when interpreting heterogeneity detection and mapping results.

## Supplementary Material

btag107_Supplementary_Data

## Data Availability

The data underlying this article cannot be shared publicly for the privacy of individuals that participated in the study. The data will be shared on reasonable request to the corresponding author.
