# Supplementary material for: Statistical methods to harmonize electronic health record data across healthcare systems: case study and lessons learned
Source: Bioinformatics. 2026 Mar 2;42(3):btag107. doi: 10.1093/bioinformatics/btag107 (PMC13005927; doi:10.1093/bioinformatics/btag107)
Supplement: btag107_Supplementary_Data [file btag107_supplementary_data.docx]

**Supplementary Material for “Harmonizing Electronic Health Record Data Across FDA Sentinel Initiative Data Partners Using Privacy-Protecting Unsupervised Learning: Case Study and Lessons Learned”**

**1. Study Population detailed definition**

KPWA and KPNW members who meet the following criteria:

- Time period for eligibility is January 1^st^, 2011, through December 31^st^, 2019 (i.e., 2011, 2012, 2013, 2014, 2015, 2016, 2017, 2018, 2019).
- Index date for sample identification (which involves the study year following the eligibility year) is December 31 of the first eligible year.
- Member must be enrolled for a minimum of 9 months in a potential eligibility year. Enrollment in the year does not need to be continuous nor enrolled at the beginning or end of the year.
- Member should be 49+ on January 1 of an eligibility year.
- Member meets the following criteria for any diabetes in an eligibility year:
- Identify patients with any diabetes per one of the criteria below in eligibility year:

**ICD-10 era**:

- - 1 in-patient (enctype = IP) claim with diabetes diagnosis code (E10/E11/E13)
  - 2 out-patient/ambulatory visit (enctype = AV) claims with diabetes diagnosis code (E10/E11/E13)
  - A1c >= 6.5%
  - Use of any diabetes medications

**ICD-9 era:**

- - 1 IP (enctype = IP) claim with diabetes diagnosis code (250.xx, 357.2, 366.41, 362.01-362.07)
  - 2 OP (enctype = AV) claims with diabetes diagnosis code (250.xx, 357.2, 366.41, 362.01-362.07)
  - A1c >= 6.5%
  - Use of any diabetes medications
- Have at least one month of observed data during the study year. Observed data refers to periods during which a patient had active enrollment and at least one recorded healthcare encounter, diagnosis, procedure, laboratory result, or medication record during the study year.

**Note:** ICD-9 codes that appear in the ICD-10 era (i.e., after October 1st, 2015) may be used, as well as ICD-10 codes in the ICD-9 era.

**2. Details of code embedding and code mapping methods**

**2.1. Code embedding**

**2.1.1. Introduction to code embedding**

Modern natural language processing (NLP) tasks often utilize word embeddings: low-dimensional vector representations of words, such that words with similar meanings have similar “locations” or directions in the induced vector space. Based on the analogy between words in human language and codes in healthcare data, one can generate medical code embeddings using the same strategy that has been used in NLP by characterizing the co-occurrence patterns of codes appearing in patients’ medical records (Beam et al. 2020). Co-occurrence measures identify codes that are typically recorded either at the same care encounter or over a small pre-defined window of time. Intuitively, if code A and B are commonly found together at one site while code A and C are found at another site, then there is evidence that code B may have the same meaning as code C. Operationally, by constraining the dimension of the representation space, we identify codes that are endorsed with a similar context of co-occurring codes suggesting semantic similarity.

As proposed in Beam et al. (2020) and Levy and Goldberg (2014), code embeddings can be derived by factorizing a version of the pointwise mutual information (PMI) matrix computed from co-occurrence counts of each pair of codes and taking the top dimension = p eigenvectors as latent features of medical codes (see Figure S1 of the supplementary material as an illustration). The directions of these code embeddings represent the relationship and meaning of the corresponding medical codes in the same way that word embeddings represent relationships among words with similar meanings. A typical pre-processing step is to normalize the length of the p-dimensional vectors to one. The rationale is that it is only the direction that matters and carries information about code relationship and meaning -- codes with similar meanings tend to have embeddings pointing to similar directions (Xing et al. 2015).

By leveraging such a vector representation of medical codes, one can compute directional distances between any pair of codes to measure their similarity or expected proximity in the medical record. One possible distance metric is the cosine of the angle between a pair of code embedding vectors, referred to as cosine similarity, which ranges between -1 and 1 where values closer to one indicate stronger similarity between the corresponding pair of codes. Computing site-specific embeddings for the codes at each site is an initial step in representing the local medical coding “dialect” and a precursor to mitigating differences through a realignment process. In sections 2.1.2 and 2.1.3 below, we provide technical details for Step 1 (code embedding).

**2.1.2. Generating, validating, and tuning code embeddings**

We generated embeddings for ICD-9, ICD-10, and CPT codes within each system, KPWA and KPNW that select both time windows to define co-occurrence and dimensionality of embedding space. All patients with any follow-up time in a study year can contribute to the development of embeddings and the measurement of co-occurrence. We first specified a range of time windows (w = 1, 2, 4, 6, 8, 15, 31 days) rolling across the entire patient record history, within which we counted the number of appearances of each code-context pair across all patient records. Co-occurrence counts serve as a population-level summary of the pairwise relationship (“correlation”) between medical codes. We then applied the method described in Beam et al. (2020) to generate code embeddings of varying dimensions. The width of time windows, w, and the dimension of the code embeddings, p (p = 10, 30, 50, 100, 150, 200, 250, 300, 350, 400), needed to be fine-tuned to optimize knowledge extraction and code representation. For example, higher-dimensional embeddings may lead to better mapping but also come with larger variation. To select the best combination of (w, p), we generated the corresponding code embeddings and then measured their ability to group ICD-9, ICD-10, and CPT codes respectively into phecode groups and CCS groups (Zhou et al. 2022). Specifically, we computed the Area Under the ROC Curve (AUC) value based on (1) predicted score of being in the same phecode group computed as cosine similarities between code pairs and (2) correspondingly generated true outcome labels from the phecode grouping.

Because rare codes provide little information, there is insufficient power to study a mapping among such rare codes between KPWA and KPNW. We collapsed rare codes with frequency less than 10 into groups according to phecode grouping and CCS, then generated embeddings for such groups along with common codes (frequency ≥ 10). Although the embeddings for such groups of rare codes do not enter the next step of code mapping, the combined data from multiple rare codes served as informative contexts to improve embedding for common codes.

**2.1.3 Results of hyperparameter tuning**

As shown in Figure S5, embedding quality was relatively stable across a broad range of window sizes and embedding dimensions, with consistently high AUC observed for moderate window sizes and dimensions between approximately 100 and 300. Based on PMI matrix, the optimal time window within which co-occurrence counts were calculated was w=1 day and the optimal dimension of embeddings was p = 250, with a corresponding maximal AUC of 0.805 at KPWA and 0.796 at KPNW for this combination.

**2.2. Code mapping**

We provide details below for Steps 2 (space alignment) and 3 (code mapping). A comparison of different methods is summarized in Table S1.

**2.2.1 Space alignment**

Space alignment refers to a procedure used to align two sets of embedding vectors into a single “language” space, such that one can measure distances in a common space where distances are comparable across both sites. Alignment is a critical first step because two sets of code embedding spaces are generated (each from a different healthcare system) and the two spaces are not necessarily aligned. In our case study, the alignment process could in theory be done from KPWA to KPNW or vice versa. We chose to align from KPWA to KPNW for our illustration, which indicates that there is a 'reference site', KPNW, for the mapping.

We considered two methods for space alignment: projection-based alignment (PA) and rotation-based alignment (RA). Let $X_{n\times p}$ and $Y_{m\times p}$ denote the embedding matrices in p-dimensional space of n codes in KPWA and m codes in KPNW, which will be used to generate a mapping from KPWA to KPNW (i.e., mapping from X to Y). For projection-based alignment, a multivariate linear regression is fitted with one set of code embeddings as the outcome and the other set as the predictor, and the estimated coefficient matrix (of dimension p-by-p), given by $\left( X_{(c)}^{'}X_{(c)} \right)^{-1}X_{(c)}^{'}Y_{(c)},$ is then leveraged to project the predictor embeddings to the space of the outcome embeddings. Here, $X_{(c)}$ and $Y_{(c)}$ denote the submatrics of $X_{n\times p}$ and $Y_{m\times p}$ corresponding to the intersection of codes in KPWA and KPNW, respectively. The estimated coefficient matrix is then applied to $X_{n\times p}$ to produce predicted values. Such predicted values and the corresponding embedding space is closer to that of $Y_{m\times p}$ . With slight abuse of notation, we will continue to use $X$to denote the aligned vectors in the following paragraphs. For rotation-based alignment, a refined regression (often referred to as a spherical regression), with a constraint that the alignment doesn’t change the length of embeddings, is fitted instead, acknowledging that embedding vectors are typically preprocessed to be of unit length and thus lie on the surface of a sphere (Xing et al. 2015; Shi et al. 2021). The estimated coefficient matrix is a rotation matrix that does not stretch or shorten the length of the aligned vectors.

**2.2.2 Code mapping**

Once the two sets of trained code embeddings are well aligned, we learn a mapping between the two sets of codes from KPWA to KPNW, allowing for both one-to-one and one-to-many mapping relationships. It is important to note that while embedding alignments are done simultaneously on all codes, we typically generate a code mapping among codes within the same group, because codes from different groups are unlikely to have similar meanings. Specific mappings are done by finding the nearest neighbor(s) of each individual code from KPWA among the codes from KPNW, where distance is defined in two ways: directional similarity (DS) and regression similarity (RS). For code i in KPWA and code j in KPNW, let $X_{i\cdot}'$ and $Y_{j\cdot}'$ denote their (aligned) embedding vectors respectively, where " ' " denotes transpose and "i." denotes the i-th row of a matrix. Specifically, directional similarity is the most commonly used distance measure in the literature of word embedding-based machine translation. The distance is measured by the cosine similarity, given by $cosine\left( i,j \right)= X_{i\cdot}Y_{j\cdot}'$. Regression similarity corresponds to regression coefficients of regressing $X_{i\cdot}^{'}$ on $Y_{j\cdot}'$ while adjusting for other codes within the same group. Letting $Y_{s\times p}$ denote the embedding matrix of s codes within the same group, the regression similarities between $X_{i\cdot}^{'}$ and the s codes is given by $\left( Y_{s\times p}Y_{s\times p}^{'} \right)^{-1}Y_{s\times p}X_{i\cdot}'$, which is a vector of s elements with each element corresponding to the regression similarity between $X_{i\cdot}^{'}$ and the corresponding code among the s codes within the group. Note that the directional similarity can be computed for all codes simultaneously, whereas the regression similarity can only be computed within groups with a restriction that s<p, i.e., we have a large enough sample size, p, to estimate s coefficients. Mathematically, one can see that the cosine similarity essentially produces an unadjusted association between a pair of codes, while the regression similarity corresponds to an adjusted association.

Regardless of the type of distance/similarity metric, we will obtain an $n_{k}$-by-$m_{k}$ matrix of similarity measures where each element denotes the cross-site distance between the corresponding pair of $n_{k}$ codes in KPWA and $m_{k}$ codes in KPNW within the code group $k$, $k=1, ..., K$ with $K$ being the total number of code groups. We denote this similarity matrix by $\hat{\Pi}_{n_{k}\times m_{k}}$, with a higher value indicating more similar and stronger association between a pair of codes. Then for each row of the similarity matrix, one can find the elements with the largest similarity values and such elements are considered matches, i.e., nearer neighbors of the code represented by the row. Elements not identified as a match will be set to zero; this results in a mapping matrix $\tilde{\Pi}_{n_{k}\times m_{k}}$ that is sparse with many zeros. There are multiple ways to find such elements: top-K matching and thresholding, where top-K matching selects the first K elements with the highest similarities, while thresholding selects all elements whose corresponding similarities are higher than a pre-selected threshold. One method for selecting this threshold is cross-validation, which is detailed in Section 3 of the supplementary material. When none of the similarities are higher than the threshold picked, we select the element whose corresponding similarity is the highest.

We also note that so far estimation of the similarity matrix $\hat{\Pi}_{n_{k}\times m_{k}}$ is based on information about joint distributions of codes, while the marginal frequencies of code endorsements have not been utilized. Intuitively, if a code mapping can precisely link together codes from two health systems with similar meanings and utilizations, then the marginal (normalized) frequencies of such linked codes at the corresponding systems should also be similar since codes with similar meanings are used in similar ways within the same patient cohort. As such, we further proposed to refine the similarity matrix by requiring that it matches individual code frequency (normalized to sum to 1) between KPWA and KPNW. Specifically, we estimate $\hat{\Gamma}_{n_{k}\times m_{k}}=argmin_{\mu_{1}=\Gamma_{n_{k}\times m_{k}}\mu_{2}, \Gamma_{n_{k}\times m_{k}}1_{m_{k}}=1_{n_{k}}}\left| \left| \hat{\Pi}_{n_{k}\times m_{k}}-\Gamma_{n_{k}\times m_{k}} \right| \right|_{F}$, where $||\cdot||_{F}$ denotes the Frobenius distance between two matrices, and $\mu_{1}$ and $\mu_{2}$ denote the normalized code frequencies for codes within the code group $k$ at the two systems. Note that we also have a constraint that each row of $\hat{\Gamma}_{n_{k}\times m_{k}}$ sums to one, such that the elements of each row can be interpreted as weights (although not necessarily all positive). This optimization problem has a closed-form solution which is derived in Section 4 of the supplementary material. Then the same data-driven thresholding or top-K mapping is conducted to derive the final mapping matrix $\tilde{\Pi}_{n_{k}\times m_{k}}$.

**Data-driven thresholding:** Let $X_{n_{k}\times p}$ and $Y_{m_{k}\times p}$ respectively denote the embedding matrices (after space alignment) for $n_{k}$ codes in KPWA and $m_{k}$ codes in KPNW within the code group $k$, and $\hat{\Pi}_{n_{k}\times m_{k}}$ denotes the matrix of similarity measures obtained from the embedding matrices to generate a mapping from KPWA to KPNW (i.e., mapping from $X$ to $Y$) within this code group. We use cross-validation to select an optimal threshold for thresholding $\hat{\Pi}_{n_{k}\times m_{k}}$ to ultimately obtain the mapping matrix $\tilde{\Pi}_{n_{k}\times m_{k}}$.

Specifically, we use “leave-one-out” cross-validation optimizing the mean squared error for prediction of $X$, defined as $MSE(\tau)=\sum_{l=1}^{p} {\left| \left| X_{n_{k}\times1}^{(l)} -\tilde{\Pi}_{n_{k}\times m_{k}}^{(l,\tau)}Y_{m_{k}\times1}^{(l)} \right| \right|^{2}}$, where $X_{n_{k}\times1}^{(l)}$ and $Y_{m_{k}\times1}^{(l)}$ denote the selected $l$-th column of $X$ and $Y$, respectively, which serve as validation data, and $\tau$ denote a certain threshold. Here, $\tilde{\Pi}_{n_{k}\times m_{k}}^{(l,\tau)}$ denotes the corresponding mapping matrix thresholding at a certain threshold $\tau$ to map from $X_{n_{k}\times(p-1)}^{(-l)}$to $Y_{m_{k}\times(p-1)}^{(-l)}$, where $X_{n_{k}\times(p-1)}^{(-l)}$ and $Y_{m_{k}\times(p-1)}^{(-l)}$ denote the remaining $(p-1)$ columns (leaving the $l$-th column out) of $X_{n_{k}\times p}$ and $Y_{m_{k}\times p}$, respectively, which serve as training data. We specify the range of potential values for $\tau$ as $\left[ 0,\max_{}\left\{ \hat{\Pi}_{n_{k}\times m_{k}} \right\} \right]$, where $\max_{}\left\{ \hat{\Pi}_{n_{k}\times m_{k}} \right\}$ denotes the maximum value of $\hat{\Pi}_{n_{k}\times m_{k}}$. The “optimal” threshold is given by $argmin_{\tau\in\left[ 0,\max_{}\left\{ \hat{\Pi}_{n_{k}\times m_{k}} \right\} \right]}\sum_{l=1}^{p} {\left| \left| X_{n_{k}\times1}^{(l)} -\tilde{\Pi}_{n_{k}\times m_{k}}^{(l,\tau)}Y_{m_{k}\times1}^{(l)} \right| \right|^{2}}$.

**4. Incorporating code marginal frequency to improve code mapping**

We note that use of a cross-site code similarity matrix is based on characteristics of codes such as the distance between them, but does not directly contain information about the overall marginal frequencies of code endorsements. Intuitively, if a code mapping can precisely link codes with similar meanings and utilizations together, then the marginal frequencies of such linked codes should also be similar because codes with similar meanings are used in similar ways within patient cohorts. Therefore, we further proposed to refine the similarity matrix by requiring that it also matches individual code frequency (normalized to sum to 1).

Specifically, we refine the similarity matrix for each code group by requiring that it matches individual code frequency (normalized to sum to one) between KPWA and KPNW, and that each row of the refined similarity matrix sums to one. Specifically, we aim to estimate $\hat{\Gamma}_{n_{k}\times m_{k}}=argmin_{\mu_{1}=\Gamma\mu_{2}, \Gamma1_{m_{k}}=1_{n_{k}}}\left| \left| \hat{\Pi}_{n_{k}\times m_{k}}-\Gamma_{n_{k}\times m_{k}} \right| \right|_{F}^{2}$, where $\left| \left| \cdot\right| \right|_{F}$ denotes the Frobenius norm, $\mu_{1}$ and $\mu_{2}$ denote the (normalized) code frequencies in KPWA and KPNW within the code group $k$, and $\hat{\Pi}_{n_{k}\times m_{k}}$ denotes the estimated similarity matrix based on information about joint distributions of codes without utilizing the marginal distributions of codes. Let $\Gamma_{i\cdot}^{'}$ denote the $i$-th row vector in $\Gamma_{n_{k}\times m_{k}}$, $\Pi_{i\cdot}^{'}$ denote the $i$-th row vector in $\Pi$, and $\mu_{1_{i}}$ denotes the $i$-th component of $\mu_{1}$. It is easy to show that the optimization problem is equivalent to $\hat{\Gamma}_{i\cdot}=argmin_{\mu_{1_{i}}=\Gamma_{i\cdot}\mu_{2}, \Gamma_{i\cdot}1_{m_{k}}=1}\left| \left| \hat{\Pi}_{i\cdot}-\Gamma_{i\cdot} \right| \right|^{2}$, for $i=1,\cdots,n_{k}$. By the method of Lagrange multipliers, we introduce two new variables $\lambda_{1}$ and $\lambda_{2}$, and solve the following three equations to find $\hat{\Gamma}_{i\cdot}$:

(1) $\frac{\partial\left\{ \left| \left| \hat{\Pi}_{i\cdot}-\Gamma_{i\cdot} \right| \right|^{2}+\lambda_{1}\left( \mu_{1_{i}}-\Gamma_{i\cdot}\mu_{2} \right)+\lambda_{2}\left( \Gamma_{i\cdot}1_{m_{k}}-1 \right) \right\}}{\partial\Gamma_{i\cdot}}=2\left( \hat{\Pi}_{i\cdot}^{'}-\Gamma_{i\cdot}^{'} \right)-\lambda_{1}\mu_{2}+\lambda_{2}1_{m_{k}}=0$;

(2) $\mu_{1_{i}}-\Gamma_{i\cdot}\mu_{2}=0$;

(3) $\Gamma_{i\cdot}1_{m_{k}}-1=0$.

From (1), we have $\Gamma_{i\cdot}^{'}=\hat{\Pi}_{i\cdot}^{'}-\lambda_{1}\mu_{2}/2+\lambda_{2}1_{m_{k}}/2$, which, without loss of generality, could be re-parameterized as $\Gamma_{i\cdot}^{'}=\hat{\Pi}_{i\cdot}^{'}+\lambda_{1}\mu_{2}+\lambda_{2}1_{{m_{k}}}$. Then from (2) and (3), we have $\lambda_{1}\left| \left| \mu_{2} \right| \right|^{2}+\lambda_{2}\mu_{2}^{'}1_{m_{k}}=\mu_{1_{i}}-\hat{\Pi}_{i\cdot}\mu_{2}$ and $\lambda_{1}\mu_{2}^{'}1_{m_{k}}+\lambda_{2}m_{k}=1-\hat{\Pi}_{i\cdot}1_{{m_{k}}}$. Therefore, $\left[ {}_{\lambda_{2}}^{\lambda_{1}} \right]=\left[ {}_{\mu_{2}^{'}1_{m_{k}} m_{k}}^{|| \mu_{2} ||^{2} \mu_{2}^{'}{1_{m_{k}}}} \right]^{-1}\left[ {}_{1-\hat{\Pi}_{i\cdot}1_{{m_{k}}}}^{\mu_{1_{i}}-\hat{\Pi}_{i\cdot}\mu_{2}} \right]$, and finally we get $\Gamma_{i\cdot}^{'}=\hat{\Pi}_{i\cdot}^{'}+\left[ \mu_{2} 1_{m_{k}} \right]\left[ {}_{\mu_{2}^{'}1_{m_{k}} m_{k}}^{|| \mu_{2} ||^{2} \mu_{2}^{'}1_{m_{k}}} \right]^{-1}\left[ {}_{1-\hat{\Pi}_{i\cdot}1_{m_{k}}}^{\mu_{1_{i}}-\hat{\Pi}_{i\cdot}\mu_{2}} \right]$.

**5. Simulation of data heterogeneity and data harmonization for transferring prediction models**

We conducted a simulation study to evaluate whether one can improve the performance of prediction models trained from one site, e.g., KPWA, when applied to another size, e.g., KPNW, by harmonizing data at the target site, KPNW, by leveraging the mapping matrix $\tilde{\Pi}$. We focus on the common situation where a small set of variables is used to create a prediction such as a computable phenotype using a regression formulation. We envision the generation or derivation of a prediction function from one site and then evaluate whether this will translate to another site where coding variation may exist. The key questions are: how much does coding variation in predictive variables degrade predictive performance (accuracy); and can mapping recover the potential loss in accuracy due to coding variation?

We started by simulating target and source data on patient level code counts as well as predicted outcomes based on a pre-specified prediction model. We then computed embeddings via decomposition of the sample covariance matrix within each site, and then estimated a mapping matrix, which is then applied back to the target data to harmonize it towards the source data. Specifically, let $f\left( X_{1}^{(1)},X_{2}^{(1)},\cdots,X_{n}^{(1)} \right)$denote the prediction model trained at the source site (site 1), where the superscript denotes variables at site 1. We consider two candidate prediction functions at the target site (site 2): the untransformed covariate prediction function $f\left( X_{1}^{(2)},X_{2}^{(2)},\cdots,X_{n}^{(2)} \right)$, which directly uses the same predictors; and the mapped covariate prediction function $h\left( Z_{1}^{(2)},Z_{2}^{(2)},\cdots,Z_{m}^{(2)} \right)$ which replaces each covariate $X_{i}^{(2)}$, with a mapped version of that covariate using the methods we outline, $Z_{j}^{(2)}$. We use both the true code mapping and the estimated code mapping in the evaluation.

We compared the true outcome and predicted outcome obtained from transferring the prediction model to harmonized target data to evaluate prediction errors. We compared three methods: (1) the “oracle” method that harmonizes data according to the true code mapping, (2) our proposed “transferred” method that harmonizes data according to the estimated code mapping, and (3) the “naïve” method that directly applies prediction model trained from source site to target site without data harmonization.

Figure S4 presents the prediction error relative to the oracle method, plotted against the proportion of code mismatch simulated in the data. We can see that the naïve method had a substantially larger amount of prediction error which indicates the consequence of data heterogeneity in transporting models from one site to another, which increased as the amount of code mismatches increased. In contrast, with data harmonization, the transferred method substantially reduced prediction error.

**6. Supplementary tables and figures**

Figure S1. Illustration of the process to generate code embeddings. First, for each pair of codes, we count how many times they co-occur within a moving time window in a patient’s record and sum up such counts across all patients within a healthcare system. Then, we derive the pointwise mutual information matrix of dimension n-by-n where n is the number of codes. Finally, the embedding is obtained from singular value decomposition of the pointwise mutual information matrix.


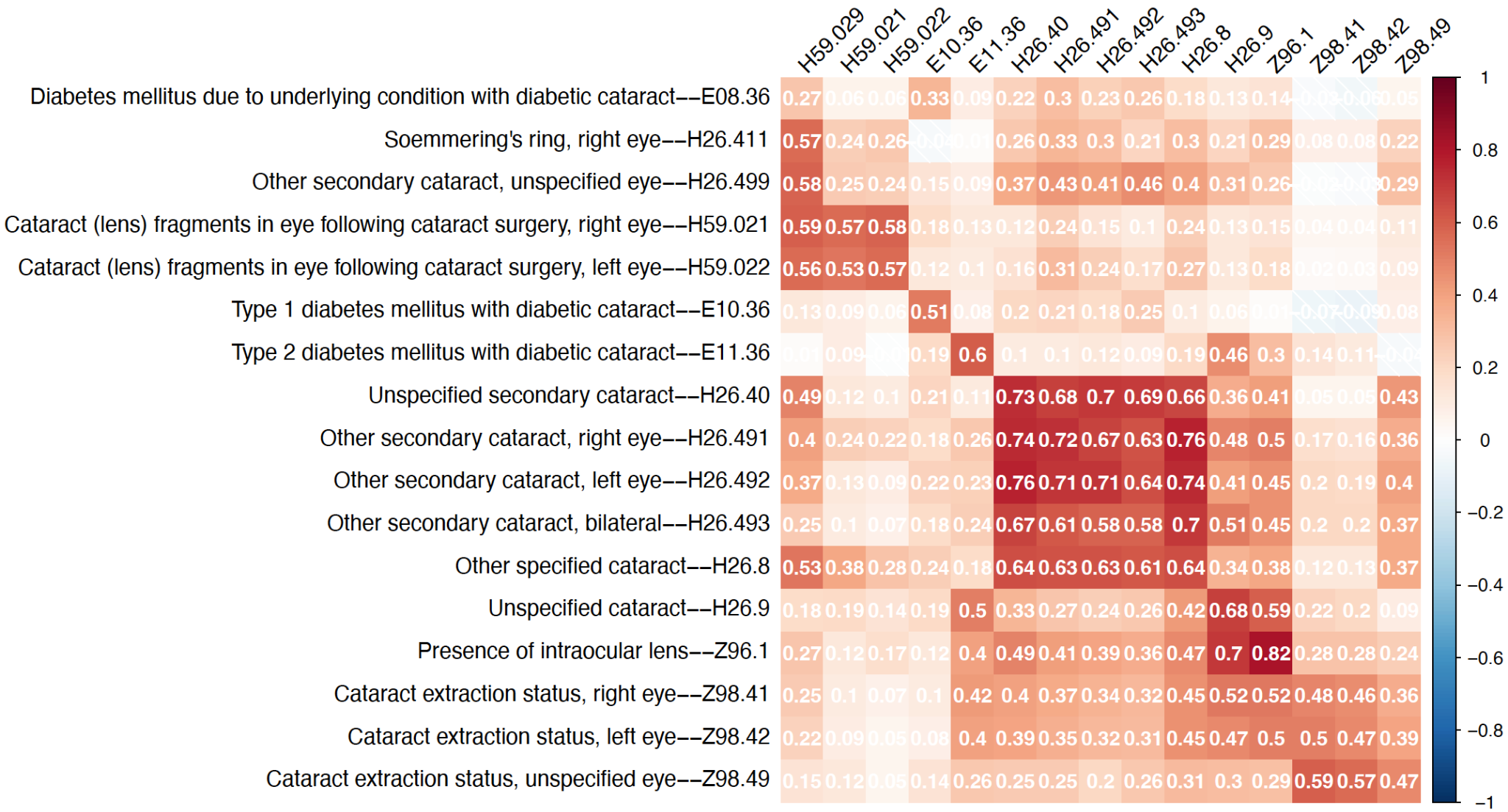

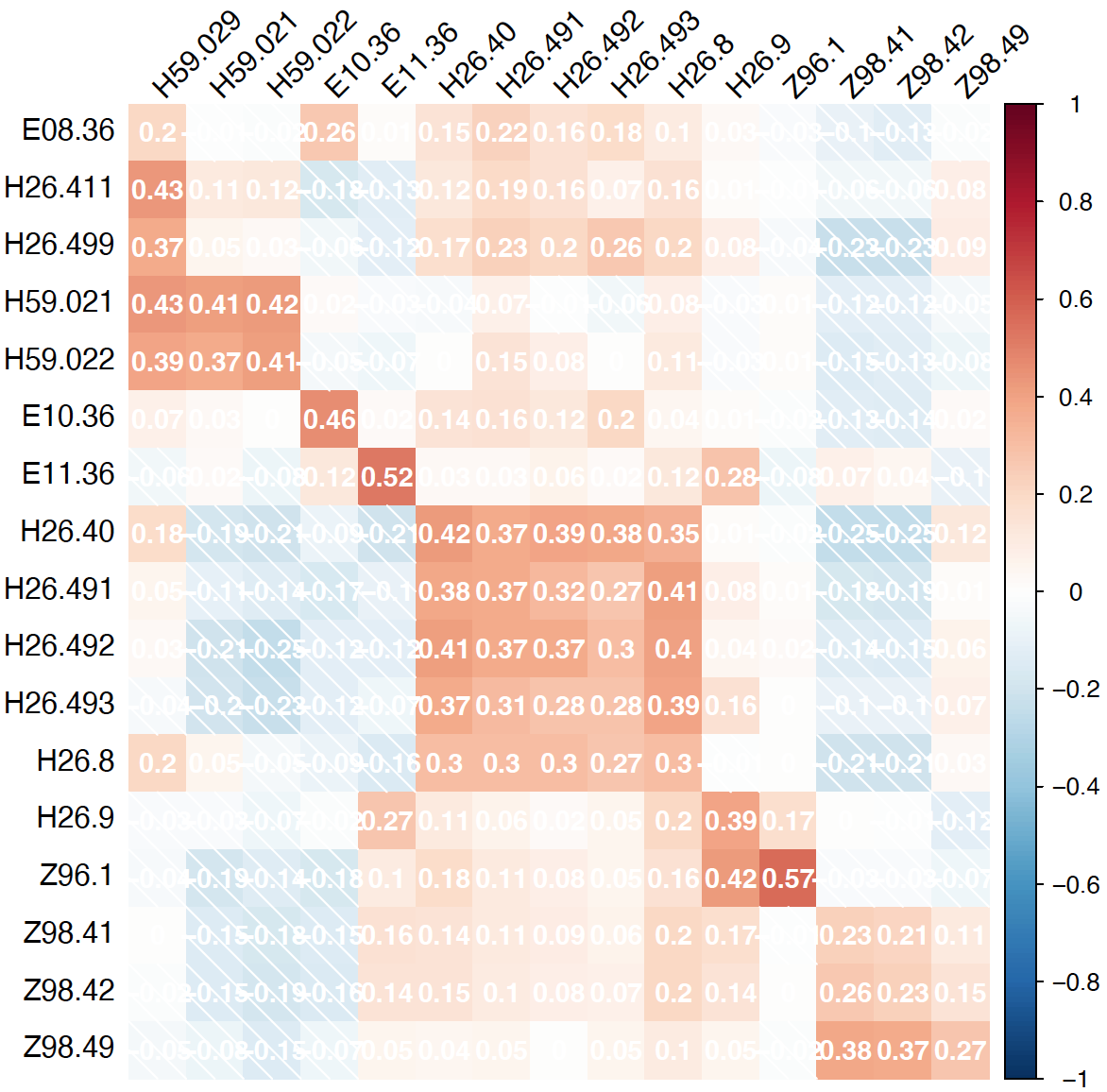


| a | b |
| --- | --- |

Figure S2. Heatmap of matrices of (a) directional similarities and (b) refined similarities (based on method detailed in Section 4 of the supplementary material) of all possible pairs of codes between KPWA and KPNW within the cataract code group. While cosine values in (a) range between -1 and 1, refined similarities in (b) can be any real number, but a higher value still indicates more similar code usage for that pair and higher likelihood to be linked in the mapping.


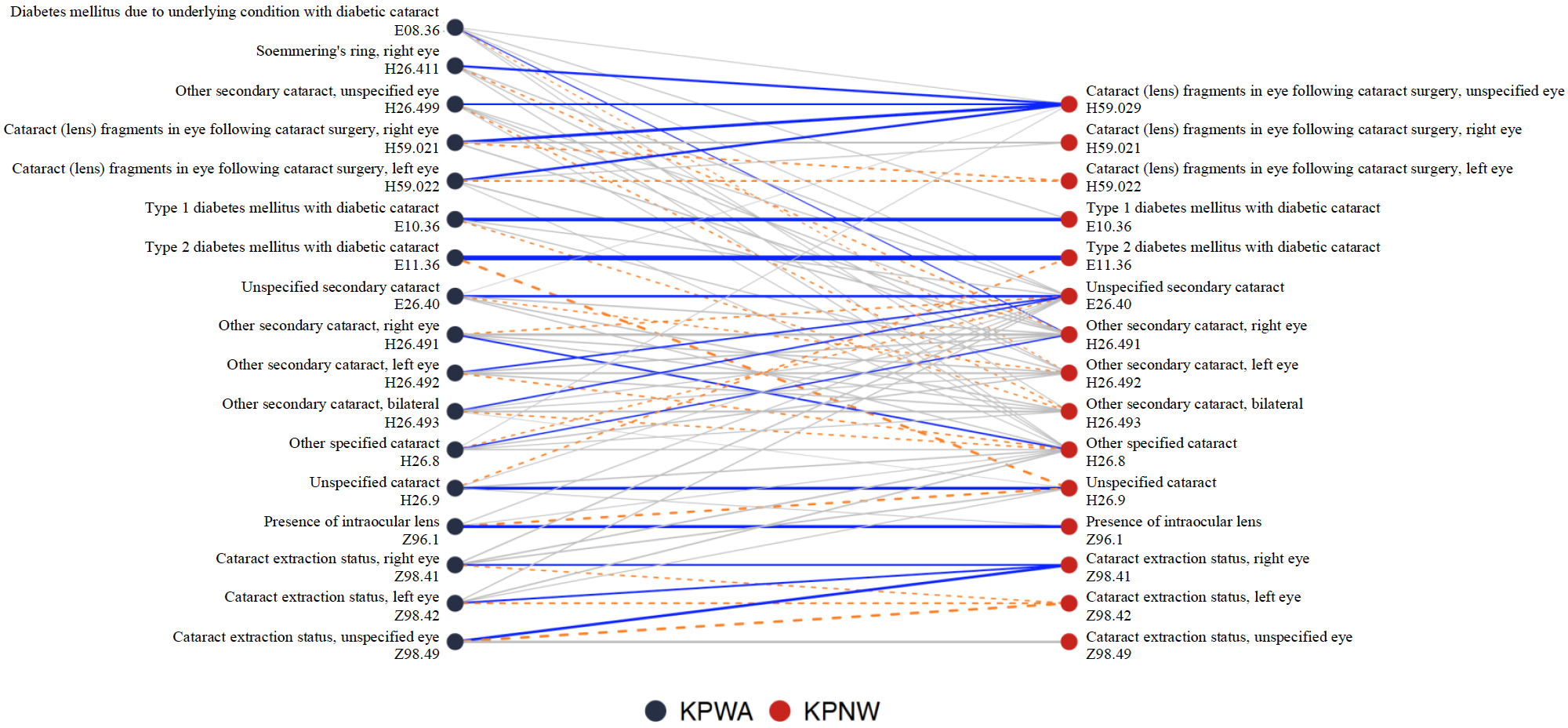


(a)


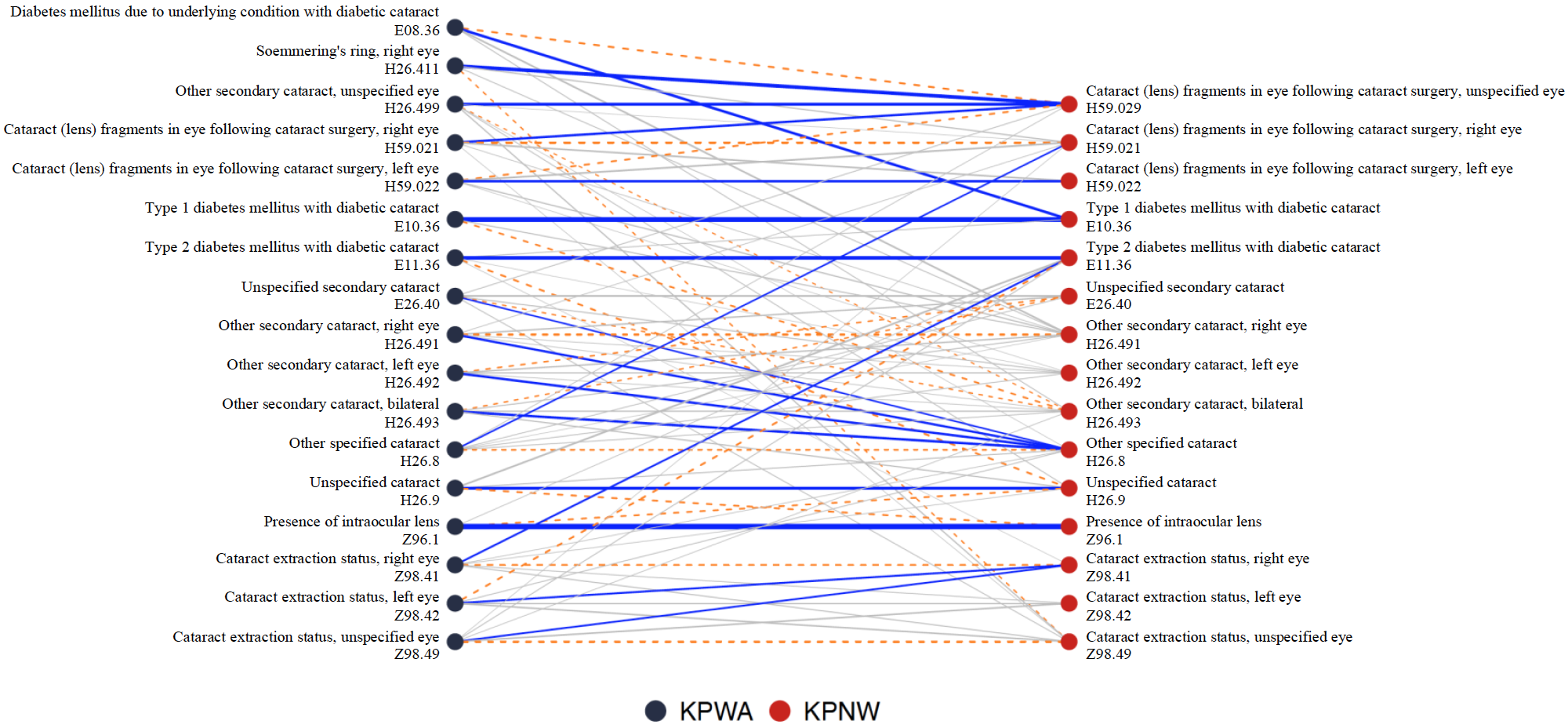


(b)

Figure S3. Mapping results from sensitivity analysis based on (a) PADS and (b) RARS methods. 17 KPWA codes are listed on the left and 15 KPNW codes are listed on the right. Gray lines represent links with similarities larger than the data-driven thresholding (which is equal to 0.16 for PADS and 0.08 for RARS), blue lines correspond to top 1 mapping, i.e., for each source code at KPWA, a blue line links it to the code at KPNW with the largest similarities as this source code, and orange dashed lines are based on selecting the second largest similarities. Line width indicates the magnitude of the similarity between the pair of linked codes.


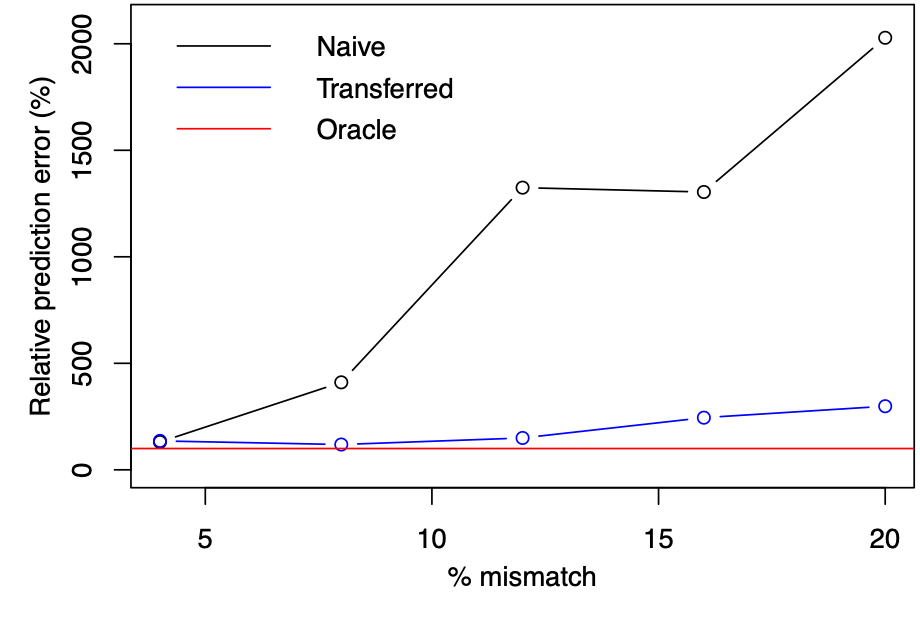


Figure S4. Results of Section 5 of the supplementary material: prediction error relative to the oracle method plotted against the proportion of code mismatch simulated in the data. naïve: fit prediction model at site 1 and apply the model to site 2; Transferred: fit prediction model at site 1, compute code mapping, and transfer the trained model using the learned mapping matrix to site 2 for prediction; Oracle: fit prediction model at site 1 and transfer the trained model using the true mapping matrix to site 2 for prediction.

| 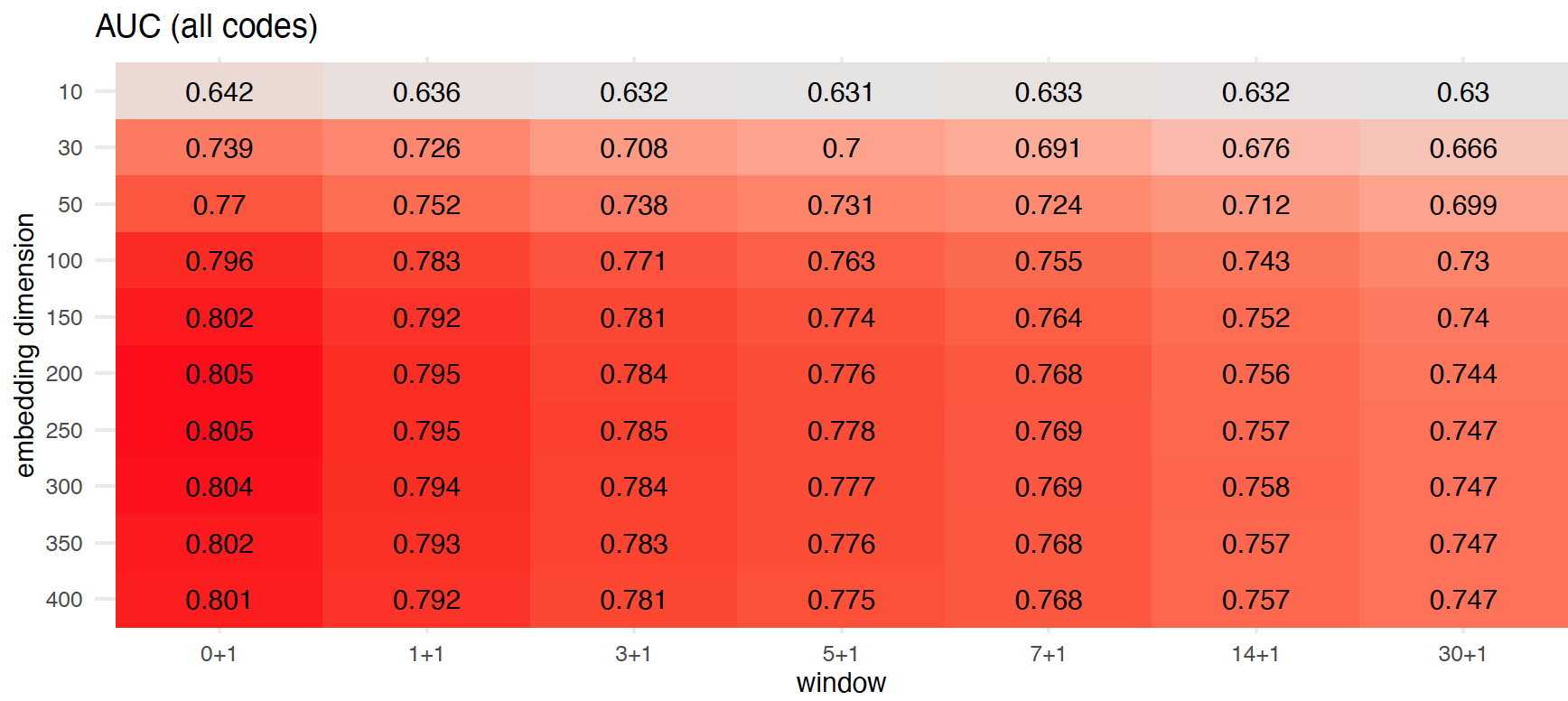 (a) PMI-based, KPWA | 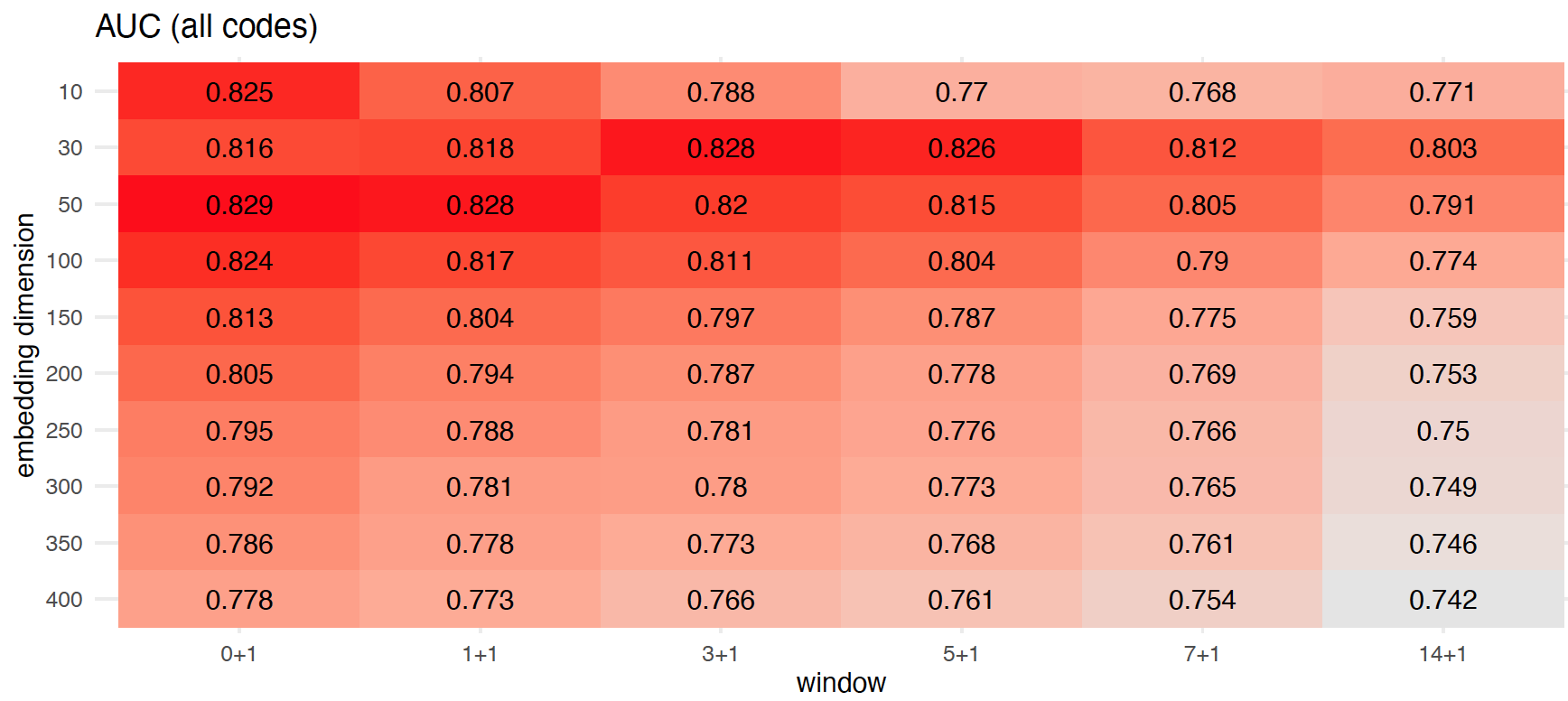 (b) SPPMI-based, KPWA |
| --- | --- |
| 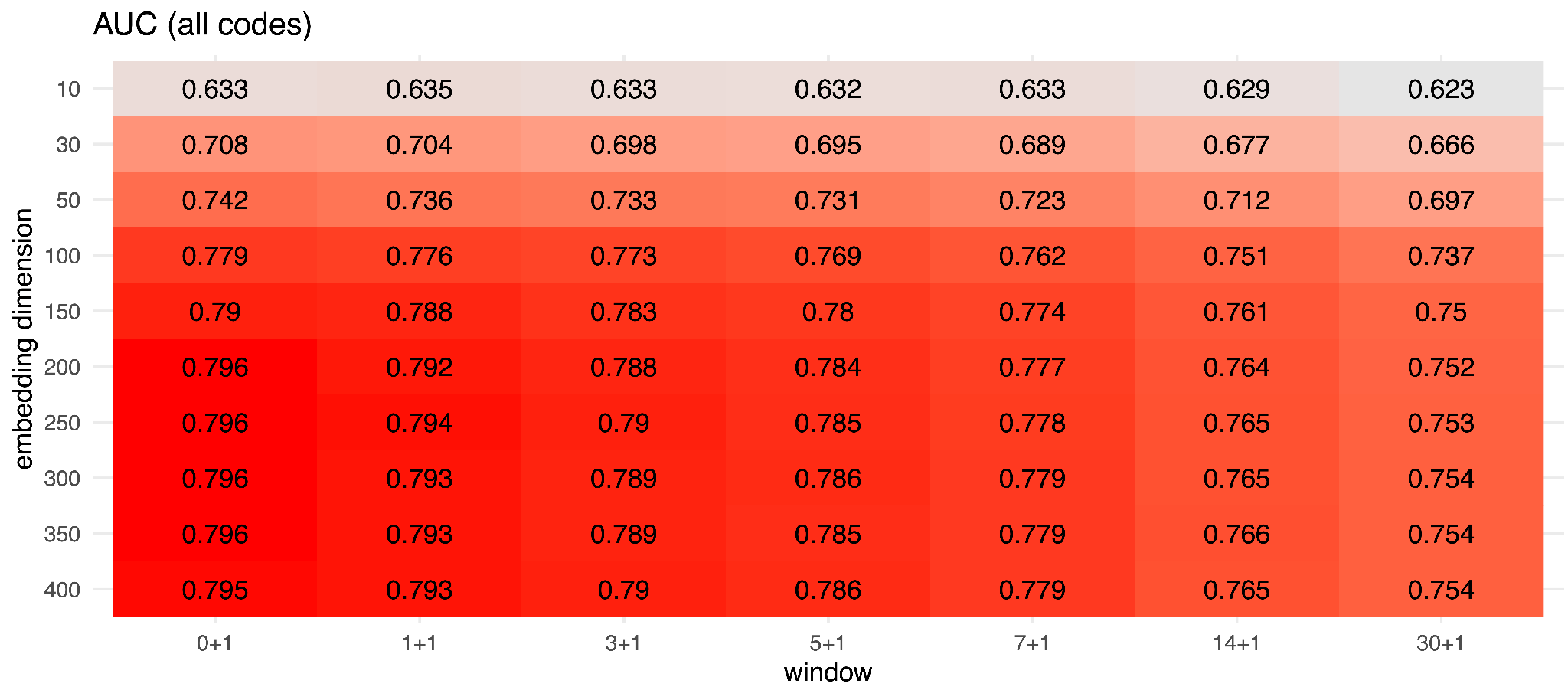 (c) PMI-based, KPNW | 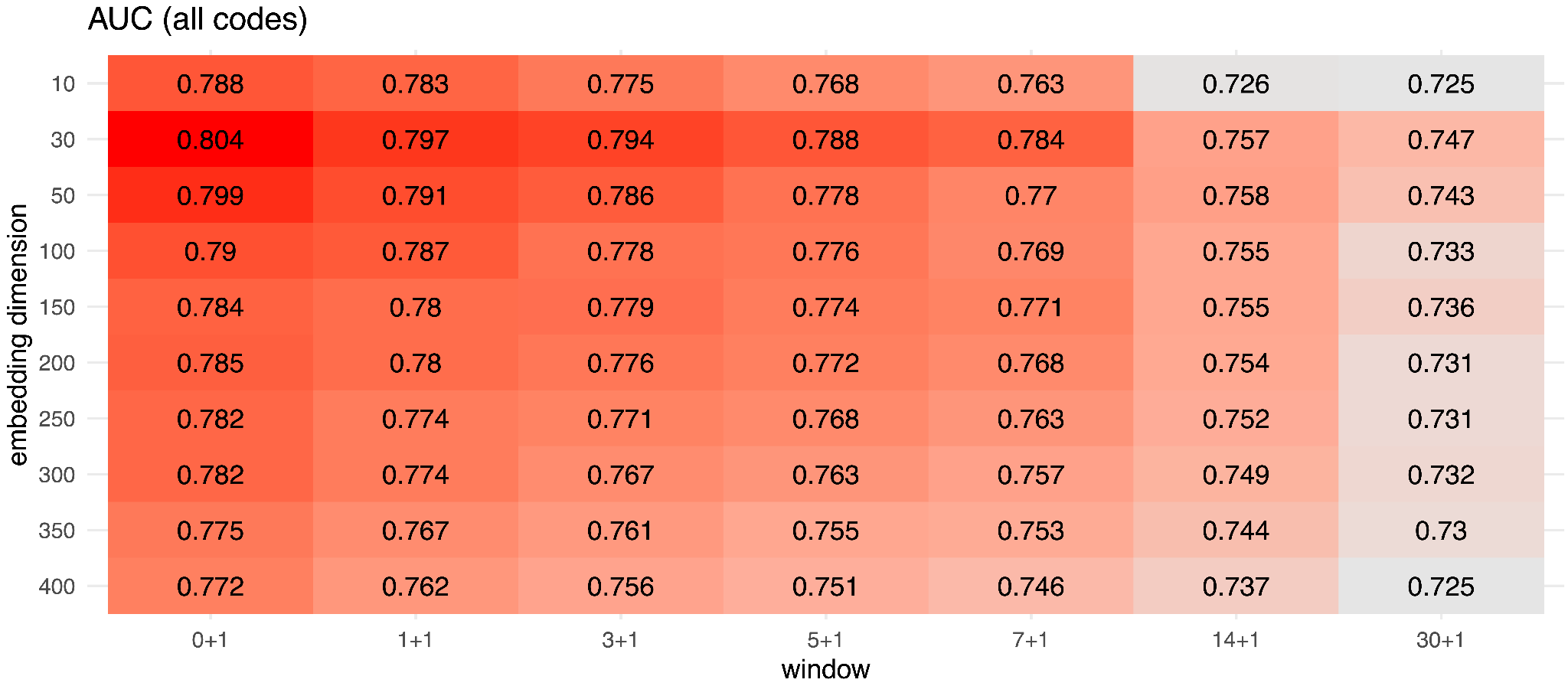 (d) SPPMI-based, KPNW |

Figure S5. Clustering AUC heatmaps assessing sensitivity of embedding quality to window size and embedding dimension for PMI- and SPPMI-based embeddings at KPNW and KPWA. Embedding quality is stable across a wide range of hyperparameters, with degradation mainly at very small dimensions or very large windows, indicating robustness of the learned semantic structure to reasonable parameter choices.

Table S1. Summary of each step for mitigating coding heterogeneity and different methods (PADS, RADS, RARS) for space alignment and code mapping.

|  | **PADS Method** | **Main Method – RADS** | **RARS Method** |
| --- | --- | --- | --- |
| **Step 0 & 1** | Data preparation & generation of code embedding | | |
| **Step 2: space alignment**  (use all codes) | **Projection-based Alignment (PA)**: linear regression to project the embeddings from one system to another | **Rotation-based Alignment (RA)**: spherical regression to rotate the embeddings from one system to another | |
| **Step 3: code mapping**  (within code group) | Find the largest **Directional Similarities (DS)**, i.e., unadjusted association between a pair of codes | | Find the largest **Regression Similarities** (RS), i.e., adjusted association between a pair of codes |

Table S2. Comparison of ICD-10 code groups between consecutive years within each health system, with Bonferroni-corrected significance level.

|  | **KPWA** | | **KPNW** | |
| --- | --- | --- | --- | --- |
|  | **SKAT** | **Burden test** | **SKAT** | **Burden test** |
| **2019 vs 2020** | 39 out of 1151 code groups (**3.39%**) have p-values <0.05/1151 | 36 out of 1151 code groups (**3.13%**) have p-values <0.05/1151 | 48 out of 1114 code groups (**4.31%**) have p-values <0.05/1114 | 40 out of 1114 code groups (**3.59%**) have p-values <0.05/1114 |
| **2018 vs 2019** | 24 out of 1161 code groups (**2.07%**) have p-values <0.05/1161 | 11 out of 1161 code groups (**0.95%**) have p-values <0.05/1161 | 29 out of 1108 code groups (**2.62%**) have p-values <0.05/1108 | 22 out of 1108 code groups (**1.99%**) have p-values <0.05/1108 |
| **2017 vs 2018** | 23 out of 1161 code groups (**2.00%**) have p-values <0.05/1161 | 9 out of 1161 code groups (**0.78%**) have p-values <0.05/1161 | 30 out of 1116 code groups (**2.69%**) have p-values <0.05/1116 | 18 out of 1116 code groups (**1.61%**) have p-values <0.05/1116 |
| **2016 vs 2017** | 49 out of 1157 code groups (**4.24%**) have p-values <0.05/1157 | 21 out of 1157 code groups (**1.82%**) have p-values <0.05/1157 | 46 out of 1109 code groups (**4.15%**) have p-values <0.05/1109 | 21 out of 1109 code groups (**1.89%**) have p-values <0.05/1109 |

**7. Computational prototypes for methods to describe (Section 2.2) and mitigate coding heterogeneity (Section 2.3)**

**7.1 Computational prototypes for methods to describe coding heterogeneity**

**Description**

This function is for the sequence kernel association test (SKAT) (Wu et al. 2011; Lee et al. 2012) or the burden test (Morgenthaler and Thilly 2007; Madsen and Browning 2009; Morris and Zeggini 2010) in the context of comparing medical code endorsement patterns between two healthcare systems adjusting for covariates (Shi et al. 2017), using summary-level data from the two systems. That is, without requiring sharing of individual-level data between sites.

Specifically, the test results are used to answer the question: considering a group of medical codes that have shared general clinical implications and measurement over a given time period, do the two systems differ in their endorsement patterns of codes?

**Usage**

SKATs(CSelect, VTest, VSelect, patientCount, measurement, Sum1d, Sum2d, method, weights.beta)

**Arguments**

**CSelect** a numeric vector of code IDs for all codes within the chosen code group.

**VTest** the name of the variable to be tested, which is the name of the variable indicating the system label of patients in our setting.

**VSelect** a vector of names of all the covariates to be adjusted for, e.g., c(“covariate1”, “covariate2”, “covariate3”)

**patientCount** a data frame with each row corresponding to a stratum specified by [VTest and VSelect] and containing the following columns: 1) column named as VTest (“SITE”) -- the system label of the patient (0 or 1); 2) columns named as VSelect -- (categorical) covariates to be adjusted for; 3) columns named as “nPatients” -- number of patients observed within the stratum specified by [VTest and VSelect]. Note: the number of rows in the data frame patientCount is the number of all possible combinations of [VTest and VSelect].

**measurement** a type of measurement for code endorsement. "count" indicates that the test will be based on the number of times that a code is assigned to a patient, and "binary" indicates that the test will be based on the binary indicator of whether a code has ever been assigned to a patient.

**Sum1d** a data frame for summary-level information about number of code endorsements, with each row corresponding to a stratum specified by [VTest, VSelect and CSelect] and containing the following columns: 1) column named as VTest -- the system label of the patient (0 or 1); 2) columns named as VSelect -- (categorical) covariates to be adjusted for; 3) column named as “CId” -- the code ID; 4) column named as “sumType_binary_sum” -- $\sum_{i\in s} I\left( T_{ij}>0 \right)$, where $T_{ij}$ is the number of times that patient 𝑖 got code 𝑗 in the selected years, and the summation is taken over patients within the stratum $s$ specified by [VTest and VSelect]; 5) column named as “sumType_count_sum” -- $\sum_{i\in s} T_{ij}$; 6) column named as “sumType_count_square_sum” $\sum_{i\in s} T_{ij}^{2}$. Note: the order of rows should be consistent with patientCount and the order of CSelect.

**Sum2d** a (q+2)-d array containing information for co-occurrence matrices within the chosen code group in each stratum specified by [VTest, VSelect and measurement], where q is the dimension of VSelect. For example, if we consider four covariates to be adjusted for (i.e., VSelect contains four elements), then 1) Sum2d[1,2,2,1,3,1,,] is the co-occurrence matrix (binary level) of codes within the chosen code group, and within the stratum of patients with the variable VTest taking the 1st level, the four covariates to be adjusted for respectively taking the 2nd, 2nd, 1st and 3rd levels; 2) Sum2d[1,2,1,2,4,2,,] is the co-occurrence matrix (count level) of codes within the chosen code group, and within the stratum of patients with VTest taking the 1st level, the four covariates to be adjusted for respectively taking the 2nd, 1st, 2nd and 4th levels. Note: the order of rows and columns in the co-occurrence matrix should be consistent with the order of CSelect.

**method** a type of test method. “SKAT” represents the sequence kernel association test, and “burden” represents the burden test. See detail section.

**weights.beta** a two-dimensional numeric vector of parameters for the beta weights for the weighted kernels. Following (Wu et al., 2011), we recommend setting the weights

using $w_{j}=\mathrm{Beta}\left( \left( \boldsymbol{1}\boldsymbol{Z}^{T}\boldsymbol{G} \right)_{j},a,b \right)$ by evaluating the density function of a Beta distribution on code frequencies. The default value is $\left( a,b \right)=\left( 1,25 \right)$. See details.

**Details**

Consider detection of association through use of a logistic regression model that

uses patients’ medical code assignment and covariates to predict the binary label of patients’ healthcare system. Explicitly, the model is

$$\mathrm{logit}\left\{ P\left( y_{i}=1|\boldsymbol{X}_{i},\boldsymbol{G}_{i} \right) \right\}=\alpha_{0}+\boldsymbol{X}_{i}\boldsymbol{\alpha}+\boldsymbol{G}_{i}\boldsymbol{\beta},i=\left\{ 1,\cdots, n \right\}$$

where $\mathrm{logit}\left( p \right)=\log\left\{ \frac{p}{1-p} \right\}$, $n$ is the total number of patients from the two systems, $y_{i}$ is the system label of the $i$th patient, which takes $0$ or $1$ respectively for the two systems, $\boldsymbol{X}_{i}=\left( x_{i1}, \cdot\cdot\cdot, x_{iq^{'}} \right)$ is a zero-one vector for categorical covariates represented by dummy variables, $\boldsymbol{G}_{i}=\left( g_{i1},\cdots,g_{ip} \right)$ is the code endorsement profile of thepatient $i$ within a pre-specified time window is represented, where $g_{ij}$ is either the number of times that code $j$ is assigned to patient $i$ or the binary indicator of whether code $j$ has ever been assigned to patient $i$. The difference between the two systems in the endorsement of the $p$ medical codes is tested with the null hypothesis $\boldsymbol{H}_{0}:\boldsymbol{\beta}=\boldsymbol{0}$.

The SKAT and the burden test make additional model assumptions to focus on detection of key alternatives. Their assumptions are different.

The SKAT assumes that each $\beta_{j}$ independently follows an arbitrary distribution with mean zero and variance $w_{j}^{2}\sigma^{2}$, where $w_{j}\geq0$ is a pre-specified weight for code $j$ typically defined based on code frequency. Under this assumption, the null hypothesis $\boldsymbol{H}_{0}:\boldsymbol{\beta}=\boldsymbol{0}$ can be equivalently written as $\boldsymbol{H}_{0}:\sigma^{2}=0$. The test statistics for the SKAT is $Q_{\mathrm{SKAT}}=\left( \boldsymbol{y}-\hat{\boldsymbol{\mu}} \right)^{T}\boldsymbol{G}\boldsymbol{W}^{2}\boldsymbol{G}^{T}\left( \boldsymbol{y}-\hat{\boldsymbol{\mu}} \right)$, where $\boldsymbol{y}=\left( y_{1},\cdots,y_{n} \right)^{T}$, $\hat{\boldsymbol{\mu}}$ is the prediction from the fitted model under $\boldsymbol{H}_{0}$, i.e., $\hat{\mu}_{i}=\mathrm{logit}^{-1}(\hat{\alpha}_{0}+\boldsymbol{X}_{i}\hat{\boldsymbol{\alpha}})$, $\boldsymbol{W}=\mathrm{diag}\left( w_{1},\cdots,w_{p} \right)$ and $\boldsymbol{G}_{n\times p}\boldsymbol{=}\left( g_{ij} \right)$. Under $\boldsymbol{H}_{0}$, $Q_{SKAT}\sim\sum_{j=1}^{p} \lambda_{j}\chi_{1,j}^{2}$, where $\chi_{1,j}^{2}$ are independent $\chi_{1}^{2}$ random variables and $\lambda_{j}$ are all eigenvalues of $\boldsymbol{W}\boldsymbol{G}^{T}\left[ \boldsymbol{V}-\boldsymbol{V}\tilde{\boldsymbol{X}}\left( {\tilde{\boldsymbol{X}}}^{T}\boldsymbol{V}\tilde{\boldsymbol{X}} \right)^{-1}{\tilde{\boldsymbol{X}}}^{T}\boldsymbol{V} \right]\boldsymbol{GW}$, where $\boldsymbol{V}=\mathrm{diag}\left( \hat{\mu}_{1}\left( 1-\hat{\mu}_{1} \right),\cdots,\hat{\mu}_{n}\left( 1-\hat{\mu}_{n} \right) \right)$ and ${\tilde{\boldsymbol{X}}}_{n\times\left( q^{'}+1 \right)}=\left[ \boldsymbol{1},\boldsymbol{X} \right]$.

The burden test assumes that $\beta_{j}=w_{j}\beta_{0}$ for some constant $\beta_{0}$. Under this assumption, the null hypothesis $\boldsymbol{H}_{0}:\boldsymbol{\beta}=\boldsymbol{0}$ is equivalent to $\boldsymbol{H}_{0}:\beta_{0}=0$. The test statistics for the burden test is $Q_{\mathrm{burden}}=\left( \boldsymbol{y}-\hat{\boldsymbol{\mu}} \right)^{T}\boldsymbol{G}\left( w_{1},\cdots,w_{p} \right)^{T}\left( w_{1},\cdots,w_{p} \right)\boldsymbol{G}^{T}\left( \boldsymbol{y}-\hat{\boldsymbol{\mu}} \right)$. Under $\boldsymbol{H}_{0}$, $Q_{\mathrm{burden}}\sim\lambda\chi_{1}^{2}$, where $\lambda=\left( w_{1},\cdots,w_{p} \right)\boldsymbol{G}^{T}\left[ \boldsymbol{V}-\boldsymbol{V}\tilde{\boldsymbol{X}}\left( {\tilde{\boldsymbol{X}}}^{T}\boldsymbol{V}\tilde{\boldsymbol{X}} \right)^{-1}{\tilde{\boldsymbol{X}}}^{T}\boldsymbol{V} \right]\boldsymbol{G}\left( w_{1},\cdots,w_{p} \right)^{T}$.

**Value**

**p.value** p-value of the SKAT or the burden test. Note that this is just the raw p-value from the test for one code group. When tests are undertaken for multiple code groups, one may need to adjust the p-values. The simplest way to do so is to use the conservative Bonferroni correction method which multiplies the raw p-values by the number of tests (i.e., the number of code groups being tested).

**Q** test statistic of the SKAT or the burden test.

**References**

Lee, S., Wu, M. C., and Lin, X. (2012). Optimal tests for rare variant effects in sequencing association studies. Biostatistics 13, 762–775.

Li, B. and Leal, S. M. (2008). Methods for detecting associations with rare variants for

common diseases: application to analysis of sequence data. The American Journal of

Human Genetics 83, 311–321.

Madsen, B. E. and Browning, S. R. (2009). A groupwise association test for rare mutations using a weighted sum statistic. PLoS genetics 5, e1000384.

Shi, X., Pashova, H., and Heagerty, P. J. (2017). Comparing healthcare utilization patterns via global differences in the endorsement of current procedural terminology codes. The Annals of Applied Statistics 11, 1349–1374.

Wu, M. C., Lee, S., Cai, T., Li, Y., Boehnke, M., and Lin, X. (2011). Rare-variant association testing for sequencing data with the sequence kernel association test. The American Journal of Human Genetics 89, 82–93.

**Examples**

########################### Test for two code groups ############################

####################### create (individual-level) example data #######################

#CIds: code IDs for all codes within the two code groups

CIds <- 1:20

#code group 1: codes with code ID $1,\cdots, 8$

#code group 2: codes with code ID $9,\cdots, 20$

CSelect_1 <- 1:8

CSelect_2 <- 9:20

VTest <- "SITE"

#two categorical covariates: age and sex

#age - three levels: <=30, 31-50, >=51

#sex - two levels: female and male

VSelect <- c("age", "sex")

#pR: patient information during all selected years, a data frame in which each row corresponds to a unique patient

#PId: the patient ID

#SITE: the system label of the patient (0 or 1)

pR <- data.frame(PId=1:200,

SITE=c(rep(0,100), rep(1,100)),

age=sample(c("<=30","31-50",">=51"), 200, replace = TRUE),

sex=sample(c("female","male"), 200, replace = TRUE))

pR$SITE <- as.factor(pR$SITE)

pR$age <- as.factor(pR$age)

pR$sex <- as.factor(pR$sex)

#YSelect: a vector of all selected years during which period the summaries are drawn

YSelect <- c(2012,2013,2014)

#cR: code record with information of CId, PId, and Year, a data frame in which each row corresponds to a code endorsement

#CId: the code ID

#PId: the patient ID

#Year: the year during which the code was assigned to the patient

cR <- data.frame(CId=NULL, PId=NULL, Year=NULL)

for (pid in 1:200) {

temp_cR_num <- sample.int(20, 1)

temp_cR <- data.frame(CId=sample(CIds, temp_cR_num, replace = TRUE),

PId=rep(pid,temp_cR_num),

Year=sample(YSelect, temp_cR_num, replace = TRUE))

cR <- rbind(cR,temp_cR)

}

############# obtain the necessary summary data for the SKAT / burden test ##############

#function “cal_patient_count” helps to get the summary data patientCount from the individual-level data pR

library(dplyr)

cal_patient_count <- function(pR,VTest,VSelect){

pR=subset(pR,select=c("PId",VTest,VSelect))

patient_count <- pR %>%

group_by_at(c(VTest,VSelect)) %>%

summarize(nPatients = length(PId))

patient_count=lapply(patient_count, function(x) { attributes(x) <- NULL; x })

patient_count=data.frame(patient_count)

strata=lapply(patient_count[,1:(dim(patient_count)[2]-1)], unique)

temp=expand.grid(strata)

patient_count<-merge(patient_count,temp,by=c(VTest,VSelect),all.x=TRUE,all.y=TRUE)

patient_count[is.na(patient_count)] <- 0

return(list(patient_count,strata))

}

patientCount_cal <- cal_patient_count(pR, VTest, VSelect)

patientCount <- patientCount_cal[[1]]

strata <- patientCount_cal[[2]]

#function “coc” returns the co-occurrence matrix at both binary and count level (combined as one matrix; the upper part is for binary level)

coc <- function(x,CSelect){

#x: code record

#calculate the co-occurrence at both binary and count level

ncode=length(CSelect)

rslt_temp= x %>% count(PId,CId)

list_temp=list()

for(icode in 1:ncode){

list_temp=append(list_temp,list(subset(rslt_temp,CId==CSelect[icode])[,c(1,3)]))

}

rslt_1=matrix(0,ncol = ncode,nrow=ncode)

rslt_2=rslt_1

for(i in 1:ncode){

for(j in i:ncode){

tempp=merge(list_temp[[i]],list_temp[[j]],by="PId")

rslt_1[i,j]=dim(tempp)[1]

rslt_2[i,j]=sum(tempp$n.x*tempp$n.y)

}

}

rslt_1=pmax(rslt_1,t(rslt_1)) #binary

rslt_2=pmax(rslt_2,t(rslt_2)) #count

return(data.frame(rbind(rslt_1,rslt_2)))

}

#function “cal_sum” returns the summary data: 1) Sum1d and 2) Sum2d

#Cal2d: if TRUE, code-pair summaries are calculated (note: code-pair summaries required only for within-group pairs)

library(reshape2)

library(tidyr)

cal_sum <- function(cR,pR,YSelect,CSelect,VTest,VSelect,strata,Cal2d=TRUE){

### Create structure of strata (add CId as another layer)

strata[[length(strata)+1]]=CSelect

names(strata)[length(strata)]="CId"

tempstr=expand.grid(strata)

if(Cal2d){

strata_2=strata

strata_2[[length(strata)+1]]=1:(2*length(CSelect))

names(strata_2)[length(strata_2)]="temp"

tempstr_2=expand.grid(strata_2)

rm(strata_2)

}

### Select data

pR=subset(pR,select=c("PId",VTest,VSelect))

cR=subset(cR,Year%in%YSelect & CId%in%CSelect)

cR=merge(cR,pR,by="PId",all.x=T,all.y = F)

### Summary in 1-d

Sum1d <- cR %>%

group_by_at(c(VTest,VSelect,"CId")) %>%

summarize(SumType_binary_sum = length(unique(PId)),

SumType_count_sum = length(PId),

SumType_count_square_sum = sum(table(PId)[]^2))

Sum1d=lapply(Sum1d, function(x) { attributes(x) <- NULL; x })

Sum1d=data.frame(Sum1d)

Sum1d <- merge(Sum1d,tempstr,by=c(VTest,VSelect,"CId"),all.x=TRUE,all.y=TRUE)

Sum1d[is.na(Sum1d)] <- 0

if(Cal2d){

### Summary in 2-d

Sum2d <- cR %>%

group_by_at(c(VTest,VSelect)) %>%

do(coc(x=., CSelect=CSelect))

Sum2d=data.frame(Sum2d)

Sum2d=lapply(Sum2d, function(x) { attributes(x) <- NULL; x })

Sum2d=data.frame(Sum2d)

#####################################

### Shape 2d summary as (q+2)-d array

dimnames=list(paste(VTest,levels(pR[,VTest,drop=T]),sep="_"))

for(Vname in VSelect){

dimnames=append(dimnames,list(paste(Vname,levels(pR[,Vname,drop=T]),sep="_")))

}

dimnames2=append(dimnames,list(c("SumType_binary_coc","SumType_count_coc")))

Summary2d=array(data=NA,dim=c(lengths(dimnames2),length(CSelect),length(CSelect)),

dimnames = append(append(dimnames2,list(CSelect)),list(CSelect)))

dM <- reshape2::melt(Sum2d, id.var=c(VTest,VSelect))

names(dM)[dim(tempstr)[2]]="CId"

dM$CId=as.numeric(dM$CId)

dM$CId=CSelect[dM$CId]

dM$temp=rep(1:(2*length(CSelect)),dim(dM)[1]/2/length(CSelect))

dM<-merge(dM,tempstr_2,by=c("CId",VTest,VSelect,"temp"),all.x=TRUE,all.y=TRUE)

dM$value[is.na(dM$value)] <- 0

dM=dM%>%arrange_at(c("CId",VTest,VSelect,"temp"))

ar1 <- array(dM$value, dim=c(2*length(CSelect),lengths(strata)[(length(strata)-1):1],length(CSelect)),

dimnames=append(list(c(strata[[length(strata)]],strata[[length(strata)]])),strata[c((length(strata)-1):1,length(strata))]))

ar1 <- aperm(ar1,c(length(strata):2,length(strata)+1,1))

z <- ar1[slice.index(ar1,length(strata)+1)<=length(CSelect)]

dim(z) <- c(lengths(strata),length(CSelect))

Summary2d[slice.index(Summary2d,length(strata))==1]=z

z <- ar1[slice.index(ar1,length(strata)+1)>length(CSelect)]

dim(z) <- c(lengths(strata),length(CSelect))

Summary2d[slice.index(Summary2d,length(strata))==2]=z

rm(z,Sum2d,dM,ar1)

gc()

}

if(Cal2d){

return(list(Sum1d,Summary2d))

}else{

return(Sum1d)

}

}

#for code group 1

necessary_sum <- cal_sum(cR, pR, YSelect, CSelect_1, VTest, VSelect, strata, Cal2d=TRUE)

Sum1d_1 <- necessary_sum[[1]]

Sum2d_1 <- necessary_sum[[2]]

#for code group 2

necessary_sum <- cal_sum(cR, pR, YSelect, CSelect_2, VTest, VSelect, strata, Cal2d=TRUE)

Sum1d_2 <- necessary_sum[[1]]

Sum2d_2 <- necessary_sum[[2]]

################# perform the SKAT / burden test with summary data ##################

#for code group 1

SKATs_1 <- SKATs(CSelect_1, VTest, VSelect, patientCount, measurement="count",

Sum1d_1, Sum2d_1, method="SKAT", weights.beta=c(1,25))

#you can use measurement="binary" and/or method="burden" to get other test results.

SKATs_1$p.value

#if the p-value<0.05/2, we conclude that there is a significant difference in the use of code group 1 between the two sites

#for code group 2

SKATs_2 <- SKATs(CSelect_2, VTest, VSelect, patientCount, measurement="count",

Sum1d_2, Sum2d_2, method="SKAT", weights.beta=c(1,25))

SKATs_2$p.value

#if the p-value<0.05/2, we conclude that there is a significant difference in the use of code group 2 between the two sites

############### perform the SKAT / burden test with individual-level data ###############

#this section shows how to perform the SKAT and the burden test with the individual-level data using the existing R package - SKAT.

#you can double-check whether the tests using summary data produce the same test results as the tests using individual-level data.

if(is.null(VSelect)){

temp <- "NULL"

} else {

temp <- paste(VSelect,collapse = "+")

}

temp <- paste(VTest,temp,sep="~")

obj <- SKAT_Null_Model(as.formula(temp), data=pR, out_type="D", Adjustment=F)

NPatients <- dim(pR)[1]

#test for code group 1

CSelect <- CSelect_1

#to test for code group 2, run CSelect <- CSelect_2 instead

count_sum <- matrix(0, nrow=NPatients, ncol=length(CSelect))

binary_sum <- matrix(0, nrow=NPatients, ncol=length(CSelect))

for (i in 1:NPatients) {

for (j in 1:length(CSelect)) {

count_sum[i,j] <- nrow(cR[cR$CId==CSelect[j] & cR$PId==pR$PId[i],])

binary_sum[i,j] <- ifelse(count_sum[i,j]>=1,1,0)

}

}

colnames(count_sum) <- CSelect

freq <- colSums(count_sum)/NPatients

weights.beta <- c(1,25)

weights <- dbeta(freq, weights.beta[1], weights.beta[2])

out <- SKAT(count_sum, obj, weights=weights, r.corr=0, is_check_genotype=F)

#r.corr=0 for SKAT and r.corr=1 for the burden test

#to use the binary data of code endorsement, simply substitute count_sum with binary_sum

#this is the p-value of the SKAT / burdern test for code group 1

out$p.value

**R code for the SKATs function**

library(dplyr)

library(SKAT)

Get_Lambda <- function(K){

out.s <- eigen(K,symmetric=TRUE, only.values=TRUE)

lambda1 <- out.s$values

IDX1 <- which(lambda1 >= 0)

# eigenvalue bigger than sum(eigenvalues)/1000

IDX2 <- which(lambda1 > mean(lambda1[IDX1])/100000)

if(length(IDX2) == 0){

#stop("No Eigenvalue is bigger than 0!!")

lambda <- 0

} else {

lambda <- lambda1[IDX2]

}

return(lambda)

}

Get_PValue.Lambda <- function(lambda,Q){

n1 <- length(Q)

p.val <- rep(0,n1)

p.val.liu <- rep(0,n1)

is_converge <- rep(0,n1)

p.val.liu <- Get_Liu_PVal.MOD.Lambda(Q, lambda)

for(i in 1:n1){

out <- SKAT_davies(Q[i],lambda,acc=10^(-6))#####IMPORTANT

p.val[i] <- out$Qq

is_converge[i] <- 1

# check convergence

if(length(lambda) == 1){

p.val[i] <- p.val.liu[i]

} else if(out$ifault != 0){

is_converge[i] <- 0

}

# check p-value

if(p.val[i] > 1 || p.val[i] <= 0 ){

is_converge[i]<-0

p.val[i] <- p.val.liu[i]

}

}

p.val.msg <- NULL

p.val.log <- NULL

#cat(p.val[1])

if(p.val[1] == 0){

param <- Get_Liu_Params_Mod_Lambda(lambda)

p.val.msg <- Get_Liu_PVal.MOD.Lambda.Zero(Q[1], param$muQ, param$muX, param$sigmaQ, param$sigmaX, param$l, param$d)

p.val.log <- Get_Liu_PVal.MOD.Lambda(Q[1], lambda, log.p=TRUE)[1]

}

return(list(p.value=p.val, p.val.liu=p.val.liu, is_converge=is_converge, p.val.log=p.val.log, pval.zero.msg=p.val.msg))

}

Get_Liu_PVal.MOD.Lambda <- function(Q.all, lambda, log.p=FALSE){

param <- Get_Liu_Params_Mod_Lambda(lambda)

Q.Norm <- (Q.all - param$muQ)/param$sigmaQ

Q.Norm1 <- Q.Norm * param$sigmaX + param$muX

p.value <- pchisq(Q.Norm1, df = param$l,ncp=param$d, lower.tail=FALSE, log.p=log.p)

return(p.value)

}

Get_Liu_Params_Mod_Lambda <- function(lambda){

## Helper function for getting the parameters for the null approximation

c1 <- rep(0,4)

for(i in 1:4){

c1[i] <- sum(lambda^i)

}

muQ <- c1[1]

sigmaQ <- sqrt(2 *c1[2])

s1 <- c1[3] / c1[2]^(3/2)

s2 <- c1[4] / c1[2]^2

beta1 <- sqrt(8)*s1

beta2 <- 12*s2

type1 <- 0

if(s1^2 > s2){

a <- 1/(s1 - sqrt(s1^2 - s2))

d <- s1 *a^3 - a^2

l <- a^2 - 2*d

} else {

type1 <- 1

l <- 1/s2

a <- sqrt(l)

d <- 0

}

muX <- l+d

sigmaX <- sqrt(2) *a

re <- list(l=l,d=d,muQ=muQ,muX=muX,sigmaQ=sigmaQ,sigmaX=sigmaX)

return(re)

}

SKAT_davies <- function(q,lambda,h = rep(1,length(lambda)),delta = rep(0,length(lambda)),sigma=0,lim=10000,acc=0.0001) {

r <- length(lambda)

if (length(h) != r) stop("lambda and h should have the same length!")

if (length(delta) != r) stop("lambda and delta should have the same length!")

out <- .C("qfc",lambdas=as.double(lambda),noncentral=as.double(delta),df=as.integer(h),r=as.integer(r),sigma=as.double(sigma),q=as.double(q),lim=as.integer(lim),acc=as.double(acc),trace=as.double(rep(0,7)),ifault=as.integer(0),res=as.double(0),PACKAGE="SKAT")

out$res <- 1 - out$res

return(list(trace=out$trace,ifault=out$ifault,Qq=out$res))

}

Get_Liu_PVal.MOD.Lambda.Zero <- function(Q, muQ, muX, sigmaQ, sigmaX, l, d){

Q.Norm <- (Q - muQ)/sigmaQ

Q.Norm1 <- Q.Norm * sigmaX + muX

temp <- c(0.05,10^-10, 10^-20,10^-30,10^-40,10^-50, 10^-60, 10^-70, 10^-80, 10^-90, 10^-100)

#qchisq(temp, df=1000000000,lower.tail=FALSE)

out <- qchisq(temp,df = l,ncp=d, lower.tail=FALSE)

IDX<-max(which(out < Q.Norm1))

pval.msg<-sprintf("Pvalue < %e", temp[IDX])

return(pval.msg)

}

SKATs <- function(CSelect, VTest, VSelect, patientCount, measurement,

Sum1d, Sum2d, method, weights.beta) {

NPatients <- sum(patientCount$nPatients)

for(Vname in VSelect){

patientCount[,Vname]=factor(patientCount[,Vname])

Sum1d[,Vname]=factor(Sum1d[,Vname])

}

patientCount <- patientCount%>%group_by_at(c(VTest,VSelect))%>%summarize(.groups='drop',nPatients=sum(nPatients))

Sum1d <- Sum1d %>%group_by_at(c("CId",VTest,VSelect))%>%summarize(.groups='drop',SumType_binary_sum=sum(SumType_binary_sum),

SumType_count_sum=sum(SumType_count_sum),

SumType_count_square_sum=sum(SumType_count_square_sum))

Sum1d <- Sum1d %>% arrange_at(c("CId",VTest,VSelect))

patientCount <- patientCount %>% arrange_at(c(VTest,VSelect))

###SKAT/Burden null

lmData <- patientCount[,c(VTest,VSelect,"nPatients")]

lmData[,1] <- lmData[,1]-1

if(is.null(VSelect)){

temp="NULL"

}else{

temp=paste(VSelect,collapse = "+")

}

temp=paste(VTest,temp,sep="~")

lmod <- glm(as.formula(temp),data=lmData, family=binomial(link = "logit"), weights=nPatients)

lmData$mu <- predict(lmod, patientCount, type="response")

Pi_1 <- lmData$mu*(1-lmData$mu)

X1 <- model.matrix(lmod) #check here that the order of the rows of X1 matches that of strata

Sum1d_temp <- subset(Sum1d,CId%in%CSelect)

Sum2d_temp <- Sum2d

temp <- match(c(VTest,VSelect),names(strata))

Sum2d_temp <- apply(Sum2d_temp,c(temp,(length(dim(Sum2d_temp))-2),(length(dim(Sum2d_temp))-1),length(dim(Sum2d_temp))),sum )

############################### Test on count

if (measurement=="count") {

count_sum_temp <- matrix(Sum1d_temp$SumType_count_sum,nrow=dim(patientCount)[1])

colnames(count_sum_temp) <- CSelect

temp <- aperm(Sum2d_temp,c(length(dim(Sum2d_temp)):1))

Sum2d_count_temp <- temp[slice.index(temp,3)==2]

dim(Sum2d_count_temp) <- c(length(CSelect),prod(dim(Sum2d_temp))/length(CSelect)/2)#cbind coc matrix over strata

###Weights

freq_temp <- colSums(count_sum_temp)/NPatients

weights <- dbeta(freq_temp,weights.beta[1],weights.beta[2])

if (method=="SKAT") {

###Test statistics

eG <- t(lmData[,1]-lmData$mu)%*%count_sum_temp #this is a summation over strata

stats <- as.numeric((eG^2)%*%matrix(weights^2,ncol=1))

stats <- stats/2 #divide by 2 to increase computational stability

###Eigenvalues

GTdiagG <- matrix(0,ncol=length(CSelect),nrow=length(CSelect))

for(stratai in 1:dim(lmData)[1]){

GTdiagG=GTdiagG + Pi_1[stratai]*Sum2d_count_temp[,(1:length(CSelect))+length(CSelect)*(stratai-1)]

}

R_temp <- X1*sqrt(Pi_1)

GMtildeG <- t(count_sum_temp*sqrt(Pi_1))%*%R_temp%*%solve(t(R_temp)%*%(R_temp*patientCount$nPatients))%*%t(R_temp)%*%(count_sum_temp*sqrt(Pi_1))

WGTPGW <- t((GTdiagG-GMtildeG)*weights)*weights

WGTPGW <- WGTPGW/2 #divide by 2 to increase computational stability

lambda <- Get_Lambda(WGTPGW)

###p-values

if(sum(lambda)!=0){

re <- Get_PValue.Lambda(lambda, stats)

p.value <- re$p.value

}

}

if (method=="burden") {

###Test statistics

eG <- t(lmData[,1]-lmData$mu)%*%count_sum_temp #this is a summation over strata

stats <- as.numeric((eG%*%matrix(weights,ncol=1))^2)

stats <- stats/2 #divide by 2 to increase computational stability

###Eigenvalues

GTdiagG <- matrix(0,ncol=length(CSelect),nrow=length(CSelect))

for(stratai in 1:dim(lmData)[1]){

GTdiagG <- GTdiagG + Pi_1[stratai]*Sum2d_count_temp[,(1:length(CSelect))+length(CSelect)*(stratai-1)]

}

R_temp <- X1*sqrt(Pi_1)

GMtildeG <- t(count_sum_temp*sqrt(Pi_1))%*%R_temp%*%solve(t(R_temp)%*%(R_temp*patientCount$nPatients))%*%t(R_temp)%*%(count_sum_temp*sqrt(Pi_1))

WGTPGW <- t((GTdiagG-GMtildeG)*weights)*weights

WGTPGW <- WGTPGW/2 #divide by 2 to increase computational stability

lambda <- sum(WGTPGW)

if(lambda!=0) {

p.value <- 1-pchisq(stats/lambda,df=1)

}

}

}

############################### Test on binary

if (measurement=="binary") {

binary_sum_temp <- matrix(Sum1d_temp$SumType_binary_sum,nrow=dim(patientCount)[1])

colnames(binary_sum_temp) <- CSelect

temp <- aperm(Sum2d_temp,c(length(dim(Sum2d_temp)):1))

Sum2d_binary_temp <- temp[slice.index(temp,3)==1]

dim(Sum2d_binary_temp) <- c(length(CSelect),prod(dim(Sum2d_temp))/length(CSelect)/2)

###Weights

freq_temp <- colSums(binary_sum_temp)/NPatients

weights <- dbeta(freq_temp,weights.beta[1],weights.beta[2])

if (method=="SKAT") {

###Test statistics

eG <- t(lmData[,1]-lmData$mu)%*%binary_sum_temp #this is a summation over strata

stats <- as.numeric((eG^2)%*%matrix(weights^2,ncol=1))

stats <- stats/2 #divide by 2 to increase computational stability

###Eigenvalues

GTdiagG <- matrix(0,ncol=length(CSelect),nrow=length(CSelect))

for(stratai in 1:dim(lmData)[1]){

GTdiagG <- GTdiagG + Pi_1[stratai]*Sum2d_binary_temp[,(1:length(CSelect))+length(CSelect)*(stratai-1)]

}

R_temp <- X1*sqrt(Pi_1)

GMtildeG <- t(binary_sum_temp*sqrt(Pi_1))%*%R_temp%*%solve(t(R_temp)%*%(R_temp*patientCount$nPatients))%*%t(R_temp)%*%(binary_sum_temp*sqrt(Pi_1))

WGTPGW <- t((GTdiagG-GMtildeG)*weights)*weights

WGTPGW <- WGTPGW/2 #divide by 2 to increase computational stability

lambda <- Get_Lambda(WGTPGW)

###p-values

if(sum(lambda)!=0){

re <- Get_PValue.Lambda(lambda, stats)

p.value <- re$p.value

}

}

if (method=="burden") {

###Test statistics

eG=t(lmData[,1]-lmData$mu)%*%binary_sum_temp #this is a summation over strata

stats <- as.numeric((eG%*%matrix(weights,ncol=1))^2)

stats <- stats/2 #divide by 2 to increase computational stability

###Eigenvalues

GTdiagG <- matrix(0,ncol=length(CSelect),nrow=length(CSelect))

for(stratai in 1:dim(lmData)[1]){

GTdiagG <- GTdiagG + Pi_1[stratai]*Sum2d_binary_temp[,(1:length(CSelect))+length(CSelect)*(stratai-1)]

}

R_temp <- X1*sqrt(Pi_1)

GMtildeG <- t(binary_sum_temp*sqrt(Pi_1))%*%R_temp%*%solve(t(R_temp)%*%(R_temp*patientCount$nPatients))%*%t(R_temp)%*%(binary_sum_temp*sqrt(Pi_1))

WGTPGW <- t((GTdiagG-GMtildeG)*weights)*weights

WGTPGW <- WGTPGW/2 #divide by 2 to increase computational stability

lambda <- sum(WGTPGW)

###p-values

if(lambda!=0){

p.value <- 1-pchisq(stats/lambda,df=1)

}

}

}

return(list(p.value=p.value,Q=stats))

}

**7.2 Computational prototypes for methods to mitigate coding heterogeneity**

**7.2.1 code embedding**

**Description**

Tools for a method inspired by natural language processing (NLP) to generate medical code embeddings by characterizing the co-occurrence patterns of codes appearing in patients’ medical records.

Specifically, function “embedding” is used to generate code embeddings with a specified dimension, based on the following code information: code frequency (marginal), group label for each code (e.g., phecode groups and Clinical Classification Software (CCS) groups), co-occurrence matrix. function “AUC_tuning” is used for parameter (width of time windows for the co-occurrence matrix and the dimension of the code embeddings) tuning by computing and comparing the Area Under the ROC Curve (AUC) value (see Details).

**Usage**

embedding(codes, coccur, freq_thresh, dim_size)

AUC_tuning(vecs_code, codes, freq_thresh, dim_sizes)

**Arguments**

1. **for embedding()**

**codes** a data frame with each row corresponding to a code and containing the following columns: 1) column named as “CId” -- the code ID; 2) column named as “freq” -- code frequency; 3) column named as “groupLabel” -- the group ID of the group to which the code belong.

**coccur** a data frame with each row corresponding to a code pair and containing the following columns: 1) column named as “code1” -- the code ID of one code within the code pair; 2) column named as “code2” -- the code ID of another code within the code pair; 3) column named as “count” -- the number of appearances of this code pair across all patient records within a selected time window.

**freq_thresh** the threshold for the code frequency. Codes with frequency less than the threshold are “rare” codes. See details.

**dim_size** the specified dimension of the code embeddings. See details.

1. **for AUC_tuning()**

**vecs_code** A matrix with each row corresponding to an embedding vector for a code. The row names are set to be code IDs.

**codes** a data frame with each row corresponding to a code and containing the following columns: 1) column named as “CId” -- the code ID; 2) column named as “freq” -- code frequency; 3) column named as “groupLabel” -- the group ID of the group to which the code belong; 4) column named as “CODE_TYPE” -- code type, e.g., “CPT” or “ICD-10”. See details.

**freq_thresh** the threshold for the code frequency. Codes with frequency less than the threshold are “rare” codes.

**dim_sizes** candidate set for the dimension of the code embeddings. The maximum should be equal to the dimension of **vecs_code**. See details.

**Details**

Computing the co-occurrence matrices using R could be extremely time-consuming in practice. So, instead, we use Python for this step. The Python code can be found in the end of this document. Basically, the input has three columns -- patient ID, date (in integer form, counting $1$ from the very first day under consideration) and code ID, and the output has four columns -- ID of code 1, ID of code 2, total number of co-occurrences, time window (under which the co-occurrence of corresponding codes is counted).

Based on the analogy between words in human language and codes in healthcare data, one can generate medical code embeddings using the same strategy that has been used in NLP by characterizing the co-occurrence patterns of codes appearing in patients’ medical records (Beam et al. 2020). Co-occurrence measures identify codes that are typically recorded either at the same care encounter or over a small pre-defined window of time. Intuitively, if code A and B are commonly found together at one site while code A and C are found at another site, then there is evidence that code B may have the same meaning as code C. Operationally, by constraining the dimension of the representation space, we identify codes that are endorsed with a similar context of co-occurring codes suggesting semantic similarity. As proposed in Beam et al. (2020) and Levy and Goldberg (2014), code embeddings can be derived by factorizing a version of the pointwise mutual information (PMI) matrix computed from co-occurrence counts of each pair of codes and taking the top d eigenvectors as latent features of medical codes. The directions of these code embeddings represent the relationship and meaning of the corresponding medical codes in the same way that word embeddings represent relationships among words with similar meanings. A typical pre-processing step is to normalize the length of the d-dimensional vectors to one. The rationale is that it is only the direction that matters and carries information about code relationship and meaning -- codes with similar meanings tend to have embeddings pointing to similar directions (Xing et al. 2015).

We generate embeddings for different type of codes (e.g., ICD-9, ICD-10, and CPT codes) within a healthcare system as follows. We first define a range of time windows (e.g., $w = 1, 2, 4, 6, 8, 15$ days) rolling across the entire patient record history. Within each rolling window, we count the number of appearances of each code-context pair across all patient records, which served as a population-level summary of the pairwise relationship (“correlation”) between medical codes. We then apply the method described in Beam et al. (2020) to generate code embeddings of dimension $p$. The width of time windows, $w$, and the dimension of the code embeddings, $p$, need to be fine-tuned to optimize knowledge extraction and code representation. For example, higher-dimension embeddings may lead to better mapping but also come with larger variation. To select the best combination of ($w, p$), we generate the corresponding code embeddings and then measure their ability to group ICD-9, ICD-10, and CPT codes respectively into phecode groups and CCS groups (Zhou et al. 2022). Specifically, we compute the AUC value based on (1) predicted score of being in the same phecode group computed as cosine similarities between code pairs and (2) correspondingly generated true outcome labels from the phecode grouping.

Because rare codes provide little information, there is insufficient power to study a mapping among such rare codes between two healthcare systems. We collapse rare codes with frequency less than a threshold (e.g., ten) into groups according to phecode grouping and the CCS, then generate embeddings for such groups along with common codes (frequency larger than or equal to the threshold). Although the embeddings for such groups of rare codes do not enter the next step of code mapping, the combined data from multiple rare codes served as informative contexts to improve embedding for common codes.

**Value**

1. **for embedding()**

**vecs_code** A matrix with each row corresponding to an embedding vector for a code. The row names are set to be code IDs.

1. **for AUC_tuning()**

**auc_icd9** A vector of AUC values computed for corresponding dimensions of the code embeddings; for ICD-9 codes only.

**auc_icd10** A vector of AUC values computed for corresponding dimensions of the code embeddings; for ICD-10 codes only.

**auc_cpt** A vector of AUC values computed for corresponding dimensions of the code embeddings; for CPT codes only.

**auc_Rec** A vector of AUC values computed for corresponding dimensions of the code embeddings; for all types of codes.

**References**

Beam, A. L., Kompa, B., Schmaltz, A., Fried, I., Weber, G., Palmer, N., Shi, X., Cai, T., & Kohane, I. S. (2020). Clinical Concept Embeddings Learned from Massive Sources of Multimodal Medical Data. *Pacific Symposium on Biocomputing. Pacific Symposium on Biocomputing*, *25*, 295–306.

Levy, O. & Goldberg, Y. (2014). Neural word embedding as implicit matrix factorization. In Proceedings of the 27th International Conference on Neural Information Processing Systems - Volume 2 (NIPS'14). MIT Press, Cambridge, MA, USA, 2177–2185.

Xing, C., Wang, D., Liu, C., & Lin, Y. (2015). Normalized Word Embedding and Orthogonal Transform for Bilingual Word Translation. *North American Chapter of the Association for Computational Linguistics*.

Zhou, D., Gan, Z., Shi, X., Patwari, A., Rush, E., Bonzel, C. L., Panickan, V. A., Hong, C., Ho, Y. L., Cai, T., Costa, L., Li, X., Castro, V. M., Murphy, S. N., Brat, G., Weber, G., Avillach, P., Gaziano, J. M., Cho, K., Liao, K. P., … Cai, T. (2022). Multiview Incomplete Knowledge Graph Integration with application to cross-institutional EHR data harmonization. *Journal of biomedical informatics*, *133*, 104147.

**Examples**

############################## create example data ###############################

#code ID, frequency, and group label for all codes

codes <- data.frame(CId=1:200,

freq=sample(1:1000, 200, replace=TRUE),

groupLabel=sample(1:12, 200, replace=TRUE))

#cooccurrence matrix

coccur <- data.frame(code1=rep(1:200,each=200),

code2=rep(1:200,200),

count=sample(1:50, 200*200, replace=TRUE))

#code type: ICD-9, ICD-10, CPT

ngroup_ICD9 <- 4

ngroup_ICD10 <- 4

ngroup_CPT <- 4

codes$CODE_TYPE[codes$groupLabel%in%1:4] <- "09"

codes$CODE_TYPE[codes$groupLabel%in%5:8] <- "10"

codes$CODE_TYPE[codes$groupLabel%in%9:12] <- "CPT"

############################## generate embeddings ##############################

vecs.code <- embedding(codes, coccur, freq_thresh=10, dim_size=50)

################################ compute AUC #################################

AUC_rt <- AUC_tuning(vecs_code, codes, freq_thresh=10, dim_sizes=seq(10,50,10))

AUC_rt$auc_icd9

AUC_rt$auc_icd10

AUC_rt$auc_cpt

AUC_rt$auc_Rec

**R code for the embedding function**

library(Matrix)

library(pROC)

library(gmp)

library(irlba)

library(readr)

library(data.table)

library(dplyr)

library(hash)

library(SNFtool)

library(gtools)

library(stringr)

library(fastDummies)

options(stringsAsFactors=FALSE)

construct_pmi <- function(coccur,singletons,my.smooth=0.75){

names(coccur) = c("code1","code2","joint_count")

ind <- which(coccur$code1!=coccur$code2 &

coccur$code1%in%singletons$marg_word$code &

coccur$code2%in%singletons$marg_context$code)

coccur = coccur[ind,]

coccur$joint_count = as.numeric(coccur$joint_count)

singletons$marg_word$marg_count = as.numeric(singletons$marg_word$marg_count)

singletons$marg_context$marg_count = as.numeric(singletons$marg_context$marg_count)

pmi_df <- coccur %>%

inner_join(singletons$marg_word,by=c("code1" = "code")) %>%

rename(W=marg_count) %>%

inner_join(singletons$marg_context,by=c("code2" = "code")) %>%

rename(C=marg_count) %>%

mutate( PMI = joint_count/(W * ((C/singletons$D)^my.smooth)) ) %>%

mutate(PMI=log(PMI))

# %>%select(code1,code2,PMI)

return(pmi_df)

}

construct_sppmi <- function(pmi,k=10) {

#sppmi_df <- pmi %>%

# mutate(SPPMI = pmax(PMI - log(k),0))

sppmi_df <- pmi %>%

mutate(SPPMI = PMI)

all_words <- unique(c(sppmi_df$code1,sppmi_df$code2))

word_2_index <- 1:length(all_words)

names(word_2_index) <- all_words

i <- as.numeric(word_2_index[as.character(sppmi_df$code1)])

j <- as.numeric(word_2_index[as.character(sppmi_df$code2)])

x <- as.numeric(sppmi_df$SPPMI)

## Remove 0s ##

non_zero <- which(x != 0)

i <- i[non_zero]

j <- j[non_zero]

x <- x[non_zero]

if(max(i)<length(all_words)|max(j)<length(all_words)){

i=c(i,length(all_words))

j=c(j,length(all_words))

x=c(x,0)

}

ism <- c(i,j)

jsm <- c(j,i)

xsm <- c(x,x)

sppmi <- sparseMatrix(i=ism,j=jsm,x=xsm)

rownames(sppmi) <- all_words

colnames(sppmi) <- all_words

return(sppmi)

}

factor_sppmi <- function(sppmi,dim_size=100,iters=25,remove_empty=TRUE,use_sum=F) {

fit <- irlba(sppmi,nv=dim_size,maxit=iters,verbose=TRUE)

W <- fit$u %*% diag(sqrt(fit$d))

vecs <- W

if(use_sum) {

C <- fit$v %*% diag(sqrt(fit$d))

vecs <- vecs + C

}

rownames(vecs) <- rownames(sppmi)

if(remove_empty) {

## Remove empty word vectors ##

vecs <- vecs[which(rowSums(vecs) != 0),]

}

return(list(vecs=vecs,fit=fit))

}

getSingletonTb <- function(coccur){

marg_word = coccur %>% group_by(code1) %>% summarise(marg=sum(count))

marg_context = coccur %>% group_by(code2) %>% summarise(marg=sum(count))

names(marg_word) = c("code","marg_count")

names(marg_context) = c("code","marg_count")

D = sum(as.numeric(coccur$count))

return(list(marg_word=marg_word,marg_context=marg_context,D=D))

}

embedding <- function(codes, coccur, freq_thresh, dim_size) {

MAX_ITERS <- 2000

group_freq_thresh <- 0

codes_freq <- subset(codes,freq>=freq_thresh)

codes_infreq <- subset(codes,freq<freq_thresh)

group_by_var <- "groupLabel"

group_freq <- codes_infreq[codes_infreq$groupLabel!="NA",] %>%

group_by_at(group_by_var) %>%

summarise(Freq = sum(freq))

codes_infreq <- cbind(codes_infreq,group_freq=group_freq$Freq[match(codes_infreq$groupLabel,group_freq$groupLabel)])

codes_infreq$group_freq[is.na(codes_infreq$group_freq)] <- codes_infreq$freq[is.na(codes_infreq$group_freq)]

codes_rm_from_train <- codes_infreq$CId[codes_infreq$group_freq<group_freq_thresh]

codes_infreq <- cbind(codes_infreq,representor=NA)

for(gLabel in setdiff(unique(codes_infreq[,group_by_var]),"NA")){

representor <- codes_infreq$CId[match(gLabel,codes_infreq[,group_by_var])]

codes_infreq$representor[codes_infreq[,group_by_var]==gLabel] <- representor

}

codes_infreq_hash <- codes_infreq[!is.na(codes_infreq$representor),]

codes_infreq_hash <- hash(codes_infreq_hash$CId,paste("g",codes_infreq_hash$representor,sep=""))

coccur <- subset(coccur,(!code1%in%codes_rm_from_train)&(!code2%in%codes_rm_from_train))

coccur$code1 <- as.character(coccur$code1)

coccur$code2 <- as.character(coccur$code2)

coccur$code1 <- sapply(coccur$code1,function(x){y=codes_infreq_hash[[x]];if(is.null(y)){return(x)}else{return(y)}})

coccur$code2 <- sapply(coccur$code2,function(x){y=codes_infreq_hash[[x]];if(is.null(y)){return(x)}else{return(y)}})

coccur <- coccur%>%group_by(code1,code2)%>%summarise(count=sum(count))

singletons <- getSingletonTb(coccur)

pmi <- construct_pmi(coccur,singletons,my.smooth=0.75)

sppmi <- construct_sppmi(pmi,k=10)

rslt <- factor_sppmi(sppmi,dim_size=dim_size,iters=MAX_ITERS,remove_empty=T,use_sum=F)

rm(sppmi,pmi,singletons,coccur);gc()

vecs.code <- rslt$vecs

vecs.code <- vecs.code[as.numeric(row.names(vecs.code))%in%codes_freq$CId,]

return(vecs.code)

}

**R code for the AUC_tuning function**

library(Matrix)

library(pROC)

library(gmp)

library(irlba)

library(readr)

library(data.table)

library(dplyr)

library(hash)

library(SNFtool)

library(gtools)

library(stringr)

library(fastDummies)

cal_tcos<-function(tcl){

temp_dummies=fastDummies::dummy_cols(tcl)

temp_dummies=as.matrix(temp_dummies[,-1])

ttemp=tcrossprod(temp_dummies,NULL)

tcos=as(ttemp, "sparseMatrix")

return(tcos)

}

cal_auc<-function(V,tcos){

###auc

temp=sqrt(apply(V^2,1,sum))

cos=tcrossprod(V/temp,NULL)#cosine similarity

cos=cos[upper.tri(cos)]

auc=auc(tcos[upper.tri(tcos)], cos)

return(auc)

}

AUC_tuning <- function(vecs_code, codes, freq_thresh, dim_sizes) {

codes_freq <- subset(codes,freq>=freq_thresh)

codes_ICD9 <- subset(codes_freq,CODE_TYPE=="09"&(!groupLabel=="NA"))

codes_ICD10 <- subset(codes_freq,CODE_TYPE=="10"&(!groupLabel=="NA"))

codes_CPT <- subset(codes_freq,CODE_TYPE=="CPT"&(!groupLabel=="NA"))

vecs_code <- cbind(vecs_code,as.numeric(dimnames(vecs_code)[[1]]))

vecs_code <- vecs_code[!is.na(vecs_code[,max(dim_sizes)+1]),]

vecs_ICD9 <- vecs_code[vecs_code[,max(dim_sizes)+1]%in%codes_ICD9$CId,]

vecs_ICD10 <- vecs_code[vecs_code[,max(dim_sizes)+1]%in%codes_ICD10$CId,]

vecs_CPT <- vecs_code[vecs_code[,max(dim_sizes)+1]%in%codes_CPT$CId,]

rm(vecs_code);gc()

tcl_ICD9 <- codes_ICD9$groupLabel[match(vecs_ICD9[,max(dim_sizes)+1],codes_ICD9$CId)]

tcl_ICD10 <- codes_ICD10$groupLabel[match(vecs_ICD10[,max(dim_sizes)+1],codes_ICD10$CId)]

tcl_CPT <- codes_CPT$groupLabel[match(vecs_CPT[,max(dim_sizes)+1],codes_CPT$CId)]

###create 0-1 matrix, to which cosine matrix will be compared to

tcos_ICD9=cal_tcos(tcl_ICD9)

tcos_ICD10=cal_tcos(tcl_ICD10)

tcos_CPT=cal_tcos(tcl_CPT)

gc()

###weight for average nmi and auc

nmi_weight <- c(length(tcl_ICD9),length(tcl_ICD10),length(tcl_CPT))

auc_weight <- nmi_weight*(nmi_weight-1)/2

nmi_weight <- nmi_weight/sum(nmi_weight)

auc_weight <- auc_weight/sum(auc_weight)

###get word vecs

auc_icd9 <- rep(NA,length(dim_sizes))

auc_icd10 <- rep(NA,length(dim_sizes))

auc_cpt <- rep(NA,length(dim_sizes))

auc_Rec <- rep(NA,length(dim_sizes))

for(dimi in 1:length(dim_sizes)){

dim_size <- dim_sizes[dimi]

auc_icd9[dimi] <- cal_auc(V=vecs_ICD9[,1:dim_size],tcos=tcos_ICD9)

auc_icd10[dimi] <- cal_auc(V=vecs_ICD10[,1:dim_size],tcos=tcos_ICD10)

auc_cpt[dimi] <- cal_auc(V=vecs_CPT[,1:dim_size],tcos=tcos_CPT)

auc_Rec[dimi] <- auc_weight[1]*auc_icd9[dimi] +auc_weight[2]*auc_icd10[dimi]+auc_weight[3]*auc_cpt[dimi]

}

return(list(auc_icd9=auc_icd9, auc_icd10=auc_icd10, auc_cpt=auc_cpt, auc_Rec=auc_Rec))

}

**Python code for computing the co-occurrence matrix**

import pandas as pd

import numpy as np

import sys

import os

import datetime

from tqdm import tqdm

from collections import Counter

os.chdir('H:\Desktop\Data')

events = pd.read_csv('codeRecord.csv', header=0)

windows = [0, 1, 3, 5, 7, 14, 30] # set window size under consideration

matrices = [Counter() for _ in range(len(windows))]

bar = tqdm(total=len(events)-1)

for i in range(len(events)-1):

sid, day, code = events.iloc[i]

for j in range(i+1, len(events)):

nsid, nday, ncode = events.iloc[j]

if sid != nsid: break

diffDay = nday - day

if diffDay > windows[-1]: break

tempLoc=np.where([x>=diffDay for x in windows])[0][0]

if code < ncode: matrices[tempLoc][(code,ncode)]+=1

else: matrices[tempLoc][(ncode, code)]+=1

bar.update(1)

tempall = [pd.DataFrame.from_dict(matrices[i], orient='index').reset_index() for i in range(len(windows))]

for i in range(len(windows)):

tempall[i]['window']=windows[i]

tempall[i]['code1']=[x[0] for x in tempall[i]['index']]

tempall[i]['code2']=[x[1] for x in tempall[i]['index']]

temp=pd.concat(tempall, axis=0)

temp.shape

temp=temp.rename(columns={0: 'count'})

co_occur=temp.loc[:,['code1','code2','count','window']]

print('star write out file: ',datetime.datetime.today())

co_occur.to_csv("coc_7windows.csv", index=False)

**7.2.2 code mapping**

**Description**

Tools for generating and validating a mapping between medical codes in two healthcare systems after the code embeddings for each system are obtained.

Specifically, there are two main steps for generating a mapping: space alignment (function “space_alignment”) and code mapping (function “code_mapping”). Space alignment refers to a procedure used to align two sets of embedding vectors into a single “language” space, such that one can measure distances in a common space where distances are comparable across both systems. After the two sets of code embeddings are well aligned, code mapping (from System 1 to System 2) can be done by finding the nearest neighbor(s) of each individual code from System 1 within the codes from System 2 based on certain distance/similarity metric. We highlight that while embedding alignments are done simultaneously on all codes, code mapping is generated among codes within the same code group, because codes belonging to different groups are unlikely to have similar meanings across systems. Finally, to empirically validate the generated code mapping and evaluate how well the mapping can harmonize heterogeneous data from the two healthcare systems, we answer the question “can we confuse a system classifier after data harmonization?” (function “validation”).

We also provide some example code for data visualization, including heatmap for the distance matrix and plot for the mapping results.

**Usage**

space_alignment(vecs_code_sys1, vecs_code_sys2, align_method)

code_mapping(vecs_code_sys1_aligned, vecs_code_sys2, distance, frequency, mapping, K, thresh, lambda)

validation(code_data_sys1, code_data_sys2, mapping_mat, V, confidence)

**Arguments**

1. **for space_alignment()**

**vecs_code_sys1** A matrix with each row corresponding to an embedding vector for a code in System 1. The row names are set to be code IDs.

**vecs_code_sys2** A matrix with each row corresponding to an embedding vector for a code in System 2. The row names are set to be code IDs.

**align_method** A type of methods for space alignment. “PA” represents projection-based alignment, and “RA” represents rotation-based alignment. See details.

1. **for code_mapping()**

**vecs_code_sys1_aligned** A matrix with each row corresponding to an embedding vector (after space alignment) for a code within the selected code group in System 1. The row names are set to be code IDs.

**vecs_code_sys2** A matrix with each row corresponding to an embedding vector for a code within the selected code group in System 2. The row names are set to be code IDs.

**distance** A type of metric applied to distance between code embeddings. “DS” represents directional similarity and “RS” represents regression similarity. See details.

**frequency** A data frame with each row corresponding to a code within the selected code group and containing the following columns: 1) column named as “CId” -- the code ID; 2) column named as “freq_sys1” -- the overall marginal code frequency in System 1; 3) column named as “freq_sys2” -- the overall marginal code frequency in System 2. With such information, one can further refine the similarity matrix by requiring that it also matches individual code frequency (normalized to sum to 1). To get the code mapping results without incorporating information about marginal frequencies, simply set **frequency** = NULL. See details.

**mapping** A type of method to select matches based on the similarity matrix. “top-K” represents top-K matching which selects the first K elements with the highest (non-negative) similarities, and “thresh” represents thresholding which selects all elements whose corresponding similarities are higher than a threshold.

**K** Number of elements with the highest similarities selected when **mapping** = “top-K”. Ser **K** = NULL when **mapping** = “thresh”.

**thresh** A specified threshold when **mapping** = “thresh”. Set **thresh** = “cv” to use cross-validation as a data-driven method for selecting an “optimal” threshold. Set **thresh** = NULL when **mapping** = “top-K”.

**lambda** A specified value for lambda when **distance** = “RS”. Set **lambda** = “cv” to use cross-validation as a data-driven method for selecting an “optimal” lambda. Set **lambda** = NULL when **distance** = “DS”.

1. **for validation()**

**code_data_sys1** A matrix containing code endorsement data from System 1, with each row corresponding to a patient and each column corresponding to a code within the selected code group. Specifically, the ($i,j$)-th element could be the number of times that code $j$ is assigned to patient $i$ (or the binary indicator of whether code $j$ has ever been assigned to patient $i$).

**code_data_sys2** A matrix containing code endorsement data from System 2.

**mapping_mat** A matrix indicating the mapping results (from System 1 to System 2).

**V** A number specifying the fold number (V-fold) in cross-validated area under the ROC curve

(AUC). See details.

**confidence** A number between 0 and 1 that represents confidence level for cross-validated AUC estimates. See details.

**Details**

We considered two methods for space alignment: projection-based alignment (PA) and rotation-based alignment (RA). Briefly, projection-based alignment uses regression techniques to predict codes across sites, while rotation-based alignment includes an additional constraint that the predictions remain standardized or have a common length (Xing et al. 2015; Shi et al. 2021).

Once the two sets of trained code embeddings are well aligned, we learn a mapping between two sets of codes within a group from System 1 to System 2, allowing for both one-to-one and one-to-many mapping relationships. It is important to note that while embedding alignments are done simultaneously on all codes, we typically generate code mapping among codes within the same group, because codes from different groups are unlikely to have similar meanings. Specific mappings are done by finding the nearest neighbor(s) of each individual code from System 1 within the codes from System 2, where distance is defined in two ways: directional similarity (DS) and regression similarity (RS). Directional similarity is the most commonly used distance measure in the literature of word embedding-based machine translation. It essentially produces an unadjusted association between a pair of codes, while the regression similarity corresponds to an adjusted association.

Regardless of the type of distance/similarity metric, we will obtain a matrix of similarity measures where each element denotes the cross-site distance between the corresponding codes from System 1 and the codes in System 2 within the selected code group. Then for each row of the similarity matrix, one can find the elements with the largest similarity values and define such elements as candidate matches. There are multiple ways to select such elements: top-K matching and thresholding, where top-K matching selects the first K elements with the highest similarities, while thresholding selects all elements whose corresponding similarities are higher than a threshold. One data-driven method for selecting the threshold is cross-validation.

We also note that use of a between-site code similarity matrix is based on characteristics of codes such as the distance between them but does not directly contain information about the overall marginal frequencies of code endorsements. Intuitively, if a code mapping can precisely link codes with similar meanings and utilizations together, then the marginal frequencies of such linked codes should also be similar because codes with similar meanings are used in similar ways within the same patient cohort. As such, we further propose a method to refine the similarity matrix by requiring that it also matches individual code frequency (normalized to sum to 1). When using function “code_mapping”, one can choose whether or not to incorporate marginal frequencies into the mapping procedure.

To empirically validate the generated code mapping and evaluate how well the mapping can harmonize heterogeneous data from the two healthcare systems, we combine data from both systems and use the system indicator as the outcome label in a logistic regression model including endorsement data for all codes as predictors. For input predictors we use the raw medical code data from System 1 and the “harmonized” data from System 2. If the mapping was successful, the combined data would not be able to predict the system from which it was derived. Specifically, if the mapping achieved good data harmonization performance, then we expect the prediction performance of such a simple logistic regression to be poor, with an AUC value that is close to 0.5. We also computed the AUC value before harmonization focusing on available overlapping codes as predictors. The idea of this method is rooted in transfer learning and domain confusion. We utilize function “ci.cvAUC” in R package “cvAUC” to calculate influence curve based confidence intervals for cross-validated AUC estimates.

**Value**

1. **for space_alignment()**

**vecs_code_sys1_aligned** A matrix with each row corresponding to an embedding vector (after space alignment) for a code in System 1. The row names are set to be code IDs.

1. **for code_mapping()**

**similarity** A matrix of similarity measures where each element denotes the cross-system distance between the embeddings for the corresponding codes in System 1 and the codes in System 2 within the selected code group.

**mapping_mat** A matrix indicating the mapping results.

1. **for validation()**

**cvAUC_before** Cross-validated AUC estimate before harmonization.

**ci_before** A vector of length two containing the upper and lower bounds for the confidence interval for cross-validated AUC before harmonization.

**cvAUC_after** Cross-validated AUC estimate after harmonization.

**ci_after** A vector of length two containing the upper and lower bounds for the confidence interval for cross-validated AUC after harmonization.

**References**

LeDell E, Petersen M, van der Laan M (2022). cvAUC: Cross-Validated Area Under the ROC Curve Confidence Intervals. R package version 1.1.4. <https://CRAN.R-project.org/package=cvAUC>

Shi, X., Li, X., & Cai, T. (2021). Spherical Regression Under Mismatch Corruption With Application to Automated Knowledge Translation, J*ournal of the American Statistical Association, 116*(536), 1953–1964. <https://doi.org/10.1080/01621459.2020.1752219>

Xing, C., Wang, D., Liu, C., & Lin, Y. (2015). Normalized Word Embedding and Orthogonal Transform for Bilingual Word Translation. *North American Chapter of the Association for Computational Linguistics*.

**Examples**

############################## create example data ###############################

#code IDs for all codes

CId_all <- 1:2000

CId_all_sys1 <- sample(CId_all, sample(1900:2000,1,replace=FALSE), replace=FALSE)

CId_all_sys1 <- CId_all_sys1[order(CId_all_sys1)]

CId_all_sys2 <- sample(CId_all, sample(1900:2000,1,replace=FALSE), replace=FALSE)

CId_all_sys2 <- CId_all_sys2[order(CId_all_sys2)]

#code embeddings for all codes in each system

library(matrixStats)

vecs_code_sys1 <- matrix(NA, nrow=length(CId_all_sys1), ncol=50)

vecs_code_sys2 <- matrix(NA, nrow=length(CId_all_sys2), ncol=50)

for (i in 1:length(CId_all_sys1)) {

temp_mean <- runif(1,-0.02,0.02)

temp_sd <- runif(1,0,0.2)

vecs_code_sys1[i,] <- rnorm(50, mean=temp_mean, sd=temp_sd)

}

for (i in 1:length(CId_all_sys2)) {

temp_mean <- runif(1,-0.02,0.02)

temp_sd <- runif(1,0,0.2)

vecs_code_sys2[i,] <- rnorm(50, mean=temp_mean, sd=temp_sd)

}

row.names(vecs_code_sys1) <- CId_all_sys1

row.names(vecs_code_sys2) <- CId_all_sys2

#code_selected: code IDs for all codes within a selected code group

code_selected <- sample(CId_all, 20, replace=FALSE)

#code frequencies within a selected code group in each system

frequency <- data.frame(CId=code_selected,

freq_sys1=sample(10:10000, 20, replace=TRUE),

freq_sys2=sample(10:10000, 20, replace=TRUE))

frequency$freq_sys1[!(frequency$CId%in%CId_all_sys1)] <- 0

frequency$freq_sys2[!(frequency$CId%in%CId_all_sys2)] <- 0

frequency <- frequency[order(frequency$CId),]

#code endorsement data from each system

code_data_sys1 <- matrix(NA,

nrow=sample(20000:21000, 1, replace=FALSE),

ncol=sum(row.names(vecs_code_sys1)%in%code_selected))

code_data_sys2 <- matrix(NA,

nrow=sample(20000:21000, 1, replace=FALSE),

ncol=sum(row.names(vecs_code_sys2)%in%code_selected))

code_data_sys1 <- matrix(sample(0:20, nrow(code_data_sys1)*ncol(code_data_sys1), replace=TRUE),

nrow=nrow(code_data_sys1))

colnames(code_data_sys1) <- row.names(vecs_code_sys1)[row.names(vecs_code_sys1)%in%code_selected]

code_data_sys2 <- matrix(sample(0:20, nrow(code_data_sys2)*ncol(code_data_sys2), replace=TRUE),

nrow=nrow(code_data_sys2))

colnames(code_data_sys2) <- row.names(vecs_code_sys2)[row.names(vecs_code_sys2)%in%code_selected]

################################ space alignment ################################

vecs_code_sys1_aligned <- space_alignment(vecs_code_sys1, vecs_code_sys2, align_method=“RA”)$vecs_code_sys1_aligned

################################ code mapping #################################

#Note that code mappings will only be bone within a selected code group. The next few lines filter codes within a group

code_mapping_rt <- code_mapping(vecs_code_sys1_aligned[row.names(vecs_code_sys1_aligned)%in%code_selected,], vecs_code_sys2[row.names(vecs_code_sys2)%in%code_selected,], distance="DS", frequency, mapping="thresh", K=NULL, thresh="cv", lambda=NULL)

similarity <- code_mapping_rt$similarity

mapping_mat <- code_mapping_rt$mapping_mat

code_mapping_rt <- code_mapping(vecs_code_sys1_aligned[row.names(vecs_code_sys1_aligned)%in%code_selected,], vecs_code_sys2[row.names(vecs_code_sys2)%in%code_selected,], distance="RS", frequency, mapping="top-K", K=2, thresh=NULL, lambda="cv")

similarity <- code_mapping_rt$similarity

mapping_mat <- code_mapping_rt$mapping_mat

################################# visualization #################################

#example code for visualization of the code mapping results

library(corrplot)

library(igraph)

plot.map <- function(M,cols=c("#273046","#CB2314")){

g <- graph.incidence(M, weighted = T)

V(g)$color <- V(g)$type

V(g)$color=gsub("FALSE",cols[1],V(g)$color)

V(g)$color=gsub("TRUE",cols[2],V(g)$color)

return(g)

}

p_title <- paste("Similarity matrix in certain ICD-10 code group)\n row/column labels correspond to codes in System 1 / 2",sep="")

corrplot(round(similarity,2),method='shade',

col=COL2('RdBu', 200)[200:1], tl.srt = 45,

mar=c(0,0,4,0),title=p_title, tl.col='black',

col.lim = c(-1,1),is.corr=F,number.cex=0.9,addCoef.col="white")

g <- plot.map(M=mapping_mat)

rName <- "Syetem 1"

cName <- "Syetem 2"

cols <- c("#273046","#CB2314")

LO <- matrix(c(rep(0,dim(mapping_mat)[1]),rep(1,dim(mapping_mat)[2]),dim(mapping_mat)[1]:1,dim(mapping_mat)[2]:1),ncol=2)

Map_color_top1 <- mapping_mat

Map_color_top2 <- mapping_mat

for (i in 1:dim(mapping_mat )[1]){

Map_color_top1[i,which.max(mapping_mat [i,])] <- "blue"

Map_color_top2[i,which.max(mapping_mat [i,])] <- "blue"

if(sum(mapping_mat [i,]==1)==0){

Map_color_top2[i,order(mapping_mat [i,],decreasing=TRUE)[2]] <- "purple"

}

}

Map_color_top1 <- as.vector(t(Map_color_top1))

Map_color_top1 <- Map_color_top1[Map_color_top1!=0]

Map_color_top1[Map_color_top1!="blue"] <- "gray"

Map_color_top2 <- as.vector(t(Map_color_top2))

Map_color_top2 <- Map_color_top2[Map_color_top2!=0]

Map_color_top2[Map_color_top2!="blue" & Map_color_top2!="purple"] <- "gray"

Map_line_top2 <- ifelse(Map_color_top2=="purple",2,1)

plot.igraph(g, main=paste("Plot for mapping results"),

edge.color=Map_color_top2,

edge.lty=Map_line_top2,

edge.width=E(g)$weight*5,layout=LO,

vertex.frame.color=V(g)$color,

vertex.size=5,

vertex.label.degree=c(rep(pi,dim(mapping_mat )[1]),rep(0,dim(mapping_mat)[2])),

vertex.label.dist=2,vertex.label.color="black")

legend("bottom", legend=c(rName,cName), col=cols, bty="n", pch=20 , pt.cex=3, cex=1.2, text.col="black" ,

horiz=T, inset=c(0, -0.1))

############################### validate mapping ################################

library(cvAUC)

validate_results <- validation(code_data_sys1, code_data_sys2, mapping_mat,

V=3, confidence=0.95)

validate_results$cvAUC_before

validate_results$ci_before

validate_results$cvAUC_after

validate_results$ci_after

**R code for the space_alignment function**

space_alignment <- function (vecs_code_sys1, vecs_code_sys2, align_method) {

CId_set <- as.numeric(intersect(row.names(vecs_code_sys1),row.names(vecs_code_sys2)))

CId_set <- CId_set[order(CId_set)]

SEV_sys1 <- vecs_code_sys1[match(CId_set,as.numeric(row.names(vecs_code_sys1))),]

SEV_sys2 <- vecs_code_sys2[match(CId_set,as.numeric(row.names(vecs_code_sys2))),]

if (align_method=="RA") {

### projection to unit sphere

temp1 <- sqrt(apply(SEV_sys1^2,1,sum))

temp2 <- sqrt(apply(SEV_sys2^2,1,sum))

SEV_sys1 <- SEV_sys1/temp1

SEV_sys2 <- SEV_sys2/temp2

##################### Rotation (minimize ||SEV_sys2-SEV_sys1%*%W||_F)

temp <- t(SEV_sys1)%*%SEV_sys2

temp <- svd(temp)

temp <- temp$u%*%t(temp$v)

SEV_sys1 <- vecs_code_sys1

SEV_sys2 <- vecs_code_sys2

temp1 <- sqrt(apply(SEV_sys1^2,1,sum))

temp2 <- sqrt(apply(SEV_sys2^2,1,sum))

SEV_sys1 <- SEV_sys1/temp1

SEV_sys2 <- SEV_sys2/temp2

return(list(vecs_code_sys1_aligned=SEV_sys1%*%temp))

}

if (align_method=="PA") {

W_hat <- solve(t(SEV_sys1)%*%SEV_sys1)%*%t(SEV_sys1)%*%SEV_sys2

return(list(vecs_code_sys1_aligned=vecs_code_sys1%*%temp))

}

}

**R code for the code_mapping function**

code_mapping <- function (vecs_code_sys1_aligned, vecs_code_sys2, distance, frequency, mapping, K, thresh, lambda) {

#row normalization

temp1 <- sqrt(apply(vecs_code_sys1_aligned^2,1,sum))

temp2 <- sqrt(apply(vecs_code_sys2^2,1,sum))

vecs_code_sys1_aligned <- vecs_code_sys1_aligned/temp1

vecs_code_sys2 <- vecs_code_sys2/temp2

if (distance=="DS" & is.null(frequency) & mapping=="top-K") {

CoS <- vecs_code_sys1_aligned%*%t(vecs_code_sys2)

top_n <- K

CoS_top <- pmax(CoS,0)

for(i in 1:dim(CoS_top)[1]){

temp <- order(CoS_top[i,],decreasing = T)

CoS_top[i,temp[-(1:top_n)]] <- 0

CoS_top[i,] <- CoS_top[i,]/(sum(CoS_top[i,]))

}

return(list(similarity=CoS, mapping_mat=CoS_top))

}

if (distance=="DS" & is.null(frequency) & mapping=="thresh") {

CoS <- vecs_code_sys1_aligned%*%t(vecs_code_sys2)

if (thresh!="cv" & is.numeric(thresh)) {

top_n <- 1

CoS_top1 <- pmax(CoS,0)

for(i in 1:dim(CoS_top1)[1]){

temp <- order(CoS_top1[i,],decreasing = T)

CoS_top1[i,temp[-(1:top_n)]] <- 0

CoS_top1[i,] <- CoS_top1[i,]/(sum(CoS_top1[i,]))

}

CoS_thr <- pmax(CoS,thresh)

CoS_thr[which(CoS_thr==thresh, arr.ind=T)] <- 0

for(i in 1:dim(CoS_thr)[1]){

if(sum(CoS_thr[i,])==0) {

CoS_thr[i,] <- CoS_top1[i,]

}

}

temp <- apply(CoS_thr,1,sum)

temp[temp==0] <- 1

CoS_thr <- CoS_thr/temp

}

if (thresh=="cv") {

#### fold number = dimension of embedding vectors ####

thr_set <- seq(0,max(CoS),0.01)

cv_sum <- rep(NA,length(thr_set))

k_fold <- dim(vecs_code_sys1_aligned)[2]

cv_dim <- dim(vecs_code_sys1_aligned)[2]/k_fold

# Randomly shuffle by column

new_order <- sample(1:dim(vecs_code_sys1_aligned)[2])

vecs_code_sys2_s <- vecs_code_sys2[,new_order]

vecs_code_sys1_aligned_s <- vecs_code_sys1_aligned[,new_order]

for (thr_i in 1:length(thr_set)){

thr <- thr_set[thr_i]

cv_sum_temp <- 0

for (cv in 1:k_fold){

test_start <- (cv-1)*cv_dim+1

test_end <- cv*cv_dim

vecs_code_sys1_aligned_train <- vecs_code_sys1_aligned_s[,-(test_start:test_end)]

vecs_code_sys2_train <- vecs_code_sys2_s[,-(test_start:test_end)]

vecs_code_sys1_aligned_test <- vecs_code_sys1_aligned_s[,test_start:test_end]

vecs_code_sys2_test <- vecs_code_sys2_s[,test_start:test_end]

vecs_code_sys1_aligned_train <- vecs_code_sys1_aligned_train/sqrt(apply(vecs_code_sys1_aligned_train^2,1,sum))

vecs_code_sys2_train <- vecs_code_sys2_train/sqrt(apply(vecs_code_sys2_train^2,1,sum))

CoS_temp <- vecs_code_sys1_aligned_train%*%t(vecs_code_sys2_train)

top_n <- 1

CoS_top_temp <- pmax(CoS_temp,0)

for(i in 1:dim(CoS_top_temp)[1]){

temp=order(CoS_top_temp[i,],decreasing=T)

CoS_top_temp[i,temp[-(1:top_n)]] <- 0

CoS_top_temp[i,] <- CoS_top_temp[i,]/(sum(CoS_top_temp[i,]))

}

CoS_top1_temp <- CoS_top_temp

CoS_thr_temp <- pmax(CoS_temp,thr)

CoS_thr_temp[which(CoS_thr_temp==thr,arr.ind=T)] <- 0

for(i in 1:dim(CoS_thr_temp)[1]){

if(sum(CoS_thr_temp[i,])==0) {

CoS_thr_temp[i,] <- CoS_top1_temp[i,]

}

}

temp <- apply(CoS_thr_temp,1,sum)

temp[temp==0] <- 1

CoS_thr_temp <- CoS_thr_temp/temp

cv_sum_temp <- cv_sum_temp + (norm(vecs_code_sys1_aligned_test-CoS_thr_temp%*%vecs_code_sys2_test,type="F"))^2

}

cv_sum[thr_i] <- cv_sum_temp

}

thresh <- thr_set[which.min(cv_sum)]

CoS_thr <- pmax(CoS,thresh)

CoS_thr[which(CoS_thr==thresh, arr.ind=T)] <- 0

for(i in 1:dim(CoS_thr)[1]){

if(sum(CoS_thr[i,])==0) {

CoS_thr[i,] <- CoS_top1[i,]

}

}

temp <- apply(CoS_thr,1,sum)

temp[temp==0] <- 1

CoS_thr <- CoS_thr/temp

}

return(list(similarity=CoS, mapping_mat=CoS_thr))

}

if (distance=="DS" & !is.null(frequency) & mapping=="top-K") {

CoS <- vecs_code_sys1_aligned%*%t(vecs_code_sys2)

Gamma_tilde <- CoS

col_freq <- frequency$freq_sys2[match(row.names(vecs_code_sys2),frequency$CId)]

col_wt <- col_freq/sum(col_freq)

A <- matrix(c(t(col_wt)%*%col_wt,

dim(vecs_code_sys2)[1]*mean(col_wt),

dim(vecs_code_sys2)[1]*mean(col_wt),

dim(vecs_code_sys2)[1]),

nrow=2)

row_freq <- frequency$freq_sys1[match(row.names(vecs_code_sys1_aligned),frequency$CId)]

row_wt <- row_freq/sum(row_freq)

for (i in 1:dim(CoS)[1]){

Gamma_tilde[i,] <- CoS[i,] + cbind(col_wt,rep(1,dim(vecs_code_sys2)[1]))%*%solve(A)%*%as.matrix(c(row_wt[i]-t(as.vector(CoS[i,]))%*%col_wt,1-t(as.vector(CoS[i,]))%*%rep(1,length(col_wt))))

}

CoS <- Gamma_tilde

top_n <- K

CoS_top <- pmax(CoS,0)

for(i in 1:dim(CoS_top)[1]){

temp <- order(CoS_top[i,],decreasing = T)

CoS_top[i,temp[-(1:top_n)]] <- 0

CoS_top[i,] <- CoS_top[i,]/(sum(CoS_top[i,]))

}

return(list(similarity=CoS, mapping_mat=CoS_top))

}

if (distance=="DS" & !is.null(frequency) & mapping=="thresh") {

CoS <- vecs_code_sys1_aligned%*%t(vecs_code_sys2)

Gamma_tilde <- CoS

col_freq <- frequency$freq_sys2[match(row.names(vecs_code_sys2),frequency$CId)]

col_wt <- col_freq/sum(col_freq)

A <- matrix(c(t(col_wt)%*%col_wt,

dim(vecs_code_sys2)[1]*mean(col_wt),

dim(vecs_code_sys2)[1]*mean(col_wt),

dim(vecs_code_sys2)[1]),

nrow=2)

row_freq <- frequency$freq_sys1[match(row.names(vecs_code_sys1_aligned),frequency$CId)]

row_wt <- row_freq/sum(row_freq)

for (i in 1:dim(CoS)[1]){

Gamma_tilde[i,] <- CoS[i,] + cbind(col_wt,rep(1,dim(vecs_code_sys2)[1]))%*%solve(A)%*%as.matrix(c(row_wt[i]-t(as.vector(CoS[i,]))%*%col_wt,1-t(as.vector(CoS[i,]))%*%rep(1,length(col_wt))))

}

CoS <- Gamma_tilde

if (thresh!="cv" & is.numeric(thresh)) {

top_n <- 1

CoS_top1 <- pmax(CoS,0)

for(i in 1:dim(CoS_top1)[1]){

temp <- order(CoS_top1[i,],decreasing = T)

CoS_top1[i,temp[-(1:top_n)]] <- 0

CoS_top1[i,] <- CoS_top1[i,]/(sum(CoS_top1[i,]))

}

CoS_thr <- pmax(CoS,thresh)

CoS_thr[which(CoS_thr==thresh, arr.ind=T)] <- 0

for(i in 1:dim(CoS_thr)[1]){

if(sum(CoS_thr[i,])==0) {

CoS_thr[i,] <- CoS_top1[i,]

}

}

temp <- apply(CoS_thr,1,sum)

temp[temp==0] <- 1

CoS_thr <- CoS_thr/temp

}

if (thresh=="cv") {

#### fold number = dimension of embedding vectors ####

thr_set <- seq(0,max(CoS),0.01)

cv_sum <- rep(NA,length(thr_set))

k_fold <- dim(vecs_code_sys1_aligned)[2]

cv_dim <- dim(vecs_code_sys1_aligned)[2]/k_fold

# Randomly shuffle by column

new_order <- sample(1:dim(vecs_code_sys1_aligned)[2])

vecs_code_sys2_s <- vecs_code_sys2[,new_order]

vecs_code_sys1_aligned_s <- vecs_code_sys1_aligned[,new_order]

for (thr_i in 1:length(thr_set)){

thr <- thr_set[thr_i]

cv_sum_temp <- 0

for (cv in 1:k_fold){

test_start <- (cv-1)*cv_dim+1

test_end <- cv*cv_dim

vecs_code_sys1_aligned_train <- vecs_code_sys1_aligned_s[,-(test_start:test_end)]

vecs_code_sys2_train <- vecs_code_sys2_s[,-(test_start:test_end)]

vecs_code_sys1_aligned_test <- vecs_code_sys1_aligned_s[,test_start:test_end]

vecs_code_sys2_test <- vecs_code_sys2_s[,test_start:test_end]

vecs_code_sys1_aligned_train <- vecs_code_sys1_aligned_train/sqrt(apply(vecs_code_sys1_aligned_train^2,1,sum))

vecs_code_sys2_train <- vecs_code_sys2_train/sqrt(apply(vecs_code_sys2_train^2,1,sum))

CoS_temp <- vecs_code_sys1_aligned_train%*%t(vecs_code_sys2_train)

Gamma_tilde <- CoS_temp

col_freq <- frequency$freq_sys2[match(row.names(vecs_code_sys2_train),frequency$CId)]

col_wt <- col_freq/sum(col_freq)

A <- matrix(c(t(col_wt)%*%col_wt,

dim(vecs_code_sys2_train)[1]*mean(col_wt),

dim(vecs_code_sys2_train)[1]*mean(col_wt),

dim(vecs_code_sys2_train)[1]),

nrow=2)

row_freq <- frequency$freq_sys1[match(row.names(vecs_code_sys1_aligned_train),frequency$CId)]

row_wt <- row_freq/sum(row_freq)

for (i in 1:dim(CoS_temp)[1]){

Gamma_tilde[i,] <- CoS_temp[i,] + cbind(col_wt,rep(1,dim(vecs_code_sys2_train)[1]))%*%solve(A)%*%as.matrix(c(row_wt[i]-t(as.vector(CoS_temp[i,]))%*%col_wt,1-t(as.vector(CoS_temp[i,]))%*%rep(1,length(col_wt))))

}

CoS_temp <- Gamma_tilde

top_n <- 1

CoS_top_temp <- pmax(CoS_temp,0)

for(i in 1:dim(CoS_top_temp)[1]){

temp=order(CoS_top_temp[i,],decreasing=T)

CoS_top_temp[i,temp[-(1:top_n)]] <- 0

CoS_top_temp[i,] <- CoS_top_temp[i,]/(sum(CoS_top_temp[i,]))

}

CoS_top1_temp <- CoS_top_temp

CoS_thr_temp <- pmax(CoS_temp,thr)

CoS_thr_temp[which(CoS_thr_temp==thr,arr.ind=T)] <- 0

for(i in 1:dim(CoS_thr_temp)[1]){

if(sum(CoS_thr_temp[i,])==0) {

CoS_thr_temp[i,] <- CoS_top1_temp[i,]

}

}

temp <- apply(CoS_thr_temp,1,sum)

temp[temp==0] <- 1

CoS_thr_temp <- CoS_thr_temp/temp

cv_sum_temp <- cv_sum_temp + (norm(vecs_code_sys1_aligned_test-CoS_thr_temp%*%vecs_code_sys2_test,type="F"))^2

}

cv_sum[thr_i] <- cv_sum_temp

}

thresh <- thr_set[which.min(cv_sum)]

CoS_thr <- pmax(CoS,thresh)

CoS_thr[which(CoS_thr==thresh, arr.ind=T)] <- 0

for(i in 1:dim(CoS_thr)[1]){

if(sum(CoS_thr[i,])==0) {

CoS_thr[i,] <- CoS_top1[i,]

}

}

temp <- apply(CoS_thr,1,sum)

temp[temp==0] <- 1

CoS_thr <- CoS_thr/temp

}

return(list(similarity=CoS, mapping_mat=CoS_thr))

}

if (distance=="RS" & is.null(frequency) & mapping=="top-K" & is.numeric(lambda)) {

PI_tilde <- vecs_code_sys1_aligned%*%t(vecs_code_sys2)%*%solve(vecs_code_sys2%*%t(vecs_code_sys2)+lambda*diag(dim(vecs_code_sys2)[1]))

top_n <- K

CoS_top <- pmax(PI_tilde,0)

for(i in 1:dim(CoS_top)[1]){

temp <- order(CoS_top[i,],decreasing = T)

CoS_top[i,temp[-(1:top_n)]] <- 0

CoS_top[i,] <- CoS_top[i,]/(sum(CoS_top[i,]))

}

return(list(similarity=PI_tilde, mapping_mat=CoS_top))

}

if (distance=="RS" & is.null(frequency) & mapping=="top-K" & lambda=="cv") {

lambda_set <- seq(0,20,0.1) # candidate set for lambda; could be changed

cv_sum <- rep(NA,length(lambda_set))

k_fold <- dim(vecs_code_sys1_aligned)[2]

cv_dim <- dim(vecs_code_sys1_aligned)[2]/k_fold

# Randomly shuffle by column

new_order <- sample(1:dim(vecs_code_sys2)[2])

vecs_code_sys2_s <- vecs_code_sys2[,new_order]

vecs_code_sys1_aligned_s <- vecs_code_sys1_aligned[,new_order]

for (lambda_i in 1:length(lambda_set)){

lambda <- lambda_set[lambda_i]

cv_sum_temp <- 0

for (cv in 1:k_fold){

test_start <- (cv-1)*cv_dim+1

test_end <- cv*cv_dim

vecs_code_sys1_aligned_train <- vecs_code_sys1_aligned_s[,-(test_start:test_end)]

vecs_code_sys2_train <- vecs_code_sys2_s[,-(test_start:test_end)]

vecs_code_sys1_aligned_test <- vecs_code_sys1_aligned_s[,test_start:test_end]

vecs_code_sys2_test <- vecs_code_sys2_s[,test_start:test_end]

vecs_code_sys1_aligned_train <- vecs_code_sys1_aligned_train/sqrt(apply(vecs_code_sys1_aligned_train^2,1,sum))

vecs_code_sys2_train <- vecs_code_sys2_train/sqrt(apply(vecs_code_sys2_train^2,1,sum))

PI_tilde_temp <- vecs_code_sys1_aligned_train%*%t(vecs_code_sys2_train)%*%solve(vecs_code_sys2_train%*%t(vecs_code_sys2_train)+lambda*diag(dim(vecs_code_sys2_train)[1]))

top_n <- K

CoS_top <- pmax(PI_tilde_temp,0)

for(i in 1:dim(CoS_top)[1]){

temp <- order(CoS_top[i,],decreasing = T)

CoS_top[i,temp[-(1:top_n)]] <- 0

CoS_top[i,] <- CoS_top[i,]/(sum(CoS_top[i,]))

}

cv_sum_temp <- cv_sum_temp + (norm(vecs_code_sys1_aligned_test-CoS_top%*%vecs_code_sys2_test,type="F"))^2

}

cv_sum[lambda_i] <- cv_sum_temp

}

lambda_best <- lambda_set[which.min(cv_sum)]

PI_tilde <- vecs_code_sys1_aligned%*%t(vecs_code_sys2)%*%solve(vecs_code_sys2%*%t(vecs_code_sys2)+lambda_best*diag(dim(vecs_code_sys2)[1]))

top_n <- K

CoS_top <- pmax(PI_tilde,0)

for(i in 1:dim(CoS_top)[1]){

temp <- order(CoS_top[i,],decreasing = T)

CoS_top[i,temp[-(1:top_n)]] <- 0

CoS_top[i,] <- CoS_top[i,]/(sum(CoS_top[i,]))

}

return(list(similarity=PI_tilde, mapping_mat=CoS_top))

}

if (distance=="RS" & is.null(frequency) & mapping=="thresh" & is.numeric(lambda)) {

PI_tilde <- vecs_code_sys1_aligned%*%t(vecs_code_sys2)%*%solve(vecs_code_sys2%*%t(vecs_code_sys2)+lambda*diag(dim(vecs_code_sys2)[1]))

if (thresh!="cv" & is.numeric(thresh)) {

top_n <- 1

CoS_top1 <- pmax(PI_tilde,0)

for(i in 1:dim(CoS_top1)[1]){

temp <- order(CoS_top1[i,],decreasing = T)

CoS_top1[i,temp[-(1:top_n)]] <- 0

CoS_top1[i,] <- CoS_top1[i,]/(sum(CoS_top1[i,]))

}

CoS_thr <- pmax(PI_tilde,thresh)

CoS_thr[which(CoS_thr==thresh, arr.ind=T)] <- 0

for(i in 1:dim(CoS_thr)[1]){

if(sum(CoS_thr[i,])==0) {

CoS_thr[i,] <- CoS_top1[i,]

}

}

temp <- apply(CoS_thr,1,sum)

temp[temp==0] <- 1

CoS_thr <- CoS_thr/temp

}

if (thresh=="cv") {

#### fold number = dimension of embedding vectors ####

thr_set <- seq(0,max(PI_tilde),0.01)

cv_sum <- rep(NA,length(thr_set))

k_fold <- dim(vecs_code_sys1_aligned)[2]

cv_dim <- dim(vecs_code_sys1_aligned)[2]/k_fold

# Randomly shuffle by column

new_order <- sample(1:dim(vecs_code_sys1_aligned)[2])

vecs_code_sys2_s <- vecs_code_sys2[,new_order]

vecs_code_sys1_aligned_s <- vecs_code_sys1_aligned[,new_order]

for (thr_i in 1:length(thr_set)){

thr <- thr_set[thr_i]

cv_sum_temp <- 0

for (cv in 1:k_fold){

test_start <- (cv-1)*cv_dim+1

test_end <- cv*cv_dim

vecs_code_sys1_aligned_train <- vecs_code_sys1_aligned_s[,-(test_start:test_end)]

vecs_code_sys2_train <- vecs_code_sys2_s[,-(test_start:test_end)]

vecs_code_sys1_aligned_test <- vecs_code_sys1_aligned_s[,test_start:test_end]

vecs_code_sys2_test <- vecs_code_sys2_s[,test_start:test_end]

vecs_code_sys1_aligned_train <- vecs_code_sys1_aligned_train/sqrt(apply(vecs_code_sys1_aligned_train^2,1,sum))

vecs_code_sys2_train <- vecs_code_sys2_train/sqrt(apply(vecs_code_sys2_train^2,1,sum))

PI_tilde_temp <- SEV_kpwa_selected_train%*%t(vecs_code_sys2_train)%*%solve(vecs_code_sys2_train%*%t(vecs_code_sys2_train)+lambda*diag(dim(vecs_code_sys2_train)[1]))

top_n <- 1

CoS_top_temp <- pmax(PI_tilde_temp,0)

for(i in 1:dim(CoS_top_temp)[1]){

temp=order(CoS_top_temp[i,],decreasing=T)

CoS_top_temp[i,temp[-(1:top_n)]] <- 0

CoS_top_temp[i,] <- CoS_top_temp[i,]/(sum(CoS_top_temp[i,]))

}

CoS_top1_temp <- CoS_top_temp

CoS_thr_temp <- pmax(PI_tilde_temp,thr)

CoS_thr_temp[which(CoS_thr_temp==thr,arr.ind=T)] <- 0

for(i in 1:dim(CoS_thr_temp)[1]){

if(sum(CoS_thr_temp[i,])==0) {

CoS_thr_temp[i,] <- CoS_top1_temp[i,]

}

}

temp <- apply(CoS_thr_temp,1,sum)

temp[temp==0] <- 1

CoS_thr_temp <- CoS_thr_temp/temp

cv_sum_temp <- cv_sum_temp + (norm(vecs_code_sys1_aligned_test-CoS_thr_temp%*%vecs_code_sys2_test,type="F"))^2

}

cv_sum[thr_i] <- cv_sum_temp

}

thresh <- thr_set[which.min(cv_sum)]

CoS_thr <- pmax(PI_tilde_temp,thresh)

CoS_thr[which(CoS_thr==thresh, arr.ind=T)] <- 0

for(i in 1:dim(CoS_thr)[1]){

if(sum(CoS_thr[i,])==0) {

CoS_thr[i,] <- CoS_top1[i,]

}

}

temp <- apply(CoS_thr,1,sum)

temp[temp==0] <- 1

CoS_thr <- CoS_thr/temp

}

return(list(similarity=PI_tilde, mapping_mat=CoS_thr))

}

if (distance=="RS" & is.null(frequency) & mapping=="thresh" & lambda=="cv") {

if (thresh!="cv" & is.numeric(thresh)) {

lambda_set <- seq(0,20,0.1) # candidate set for lambda; could be changed

cv_sum <- rep(NA,length(lambda_set))

k_fold <- dim(vecs_code_sys1_aligned)[2]

cv_dim <- dim(vecs_code_sys1_aligned)[2]/k_fold

# Randomly shuffle by column

new_order <- sample(1:dim(vecs_code_sys2)[2])

vecs_code_sys2_s <- vecs_code_sys2[,new_order]

vecs_code_sys1_aligned_s <- vecs_code_sys1_aligned[,new_order]

for (lambda_i in 1:length(lambda_set)){

lambda <- lambda_set[lambda_i]

cv_sum_temp <- 0

for (cv in 1:k_fold){

test_start <- (cv-1)*cv_dim+1

test_end <- cv*cv_dim

vecs_code_sys1_aligned_train <- vecs_code_sys1_aligned_s[,-(test_start:test_end)]

vecs_code_sys2_train <- vecs_code_sys2_s[,-(test_start:test_end)]

vecs_code_sys1_aligned_test <- vecs_code_sys1_aligned_s[,test_start:test_end]

vecs_code_sys2_test <- vecs_code_sys2_s[,test_start:test_end]

vecs_code_sys1_aligned_train <- vecs_code_sys1_aligned_train/sqrt(apply(vecs_code_sys1_aligned_train^2,1,sum))

vecs_code_sys2_train <- vecs_code_sys2_train/sqrt(apply(vecs_code_sys2_train^2,1,sum))

PI_tilde_temp <- vecs_code_sys1_aligned_train%*%t(vecs_code_sys2_train)%*%solve(vecs_code_sys2_train%*%t(vecs_code_sys2_train)+lambda*diag(dim(vecs_code_sys2_train)[1]))

top_n <- 1

CoS_top1_temp <- pmax(PI_tilde_temp,0)

for(i in 1:dim(CoS_top1_temp)[1]){

temp <- order(CoS_top1_temp[i,],decreasing = T)

CoS_top1_temp[i,temp[-(1:top_n)]] <- 0

CoS_top1_temp[i,] <- CoS_top1_temp[i,]/(sum(CoS_top1_temp[i,]))

}

CoS_thr_temp <- pmax(PI_tilde_temp,thresh)

CoS_thr_temp[which(CoS_thr_temp==thresh, arr.ind=T)] <- 0

for(i in 1:dim(CoS_thr_temp)[1]){

if(sum(CoS_thr_temp[i,])==0) {

CoS_thr_temp[i,] <- CoS_top1_temp[i,]

}

}

temp <- apply(CoS_thr_temp,1,sum)

temp[temp==0] <- 1

CoS_thr_temp <- CoS_thr_temp/temp

cv_sum_temp <- cv_sum_temp + (norm(vecs_code_sys1_aligned_test-CoS_thr_temp%*%vecs_code_sys2_test,type="F"))^2

}

cv_sum[lambda_i] <- cv_sum_temp

}

lambda_best <- lambda_set[which.min(cv_sum)]

PI_tilde <- vecs_code_sys1_aligned%*%t(vecs_code_sys2)%*%solve(vecs_code_sys2%*%t(vecs_code_sys2)+lambda_best*diag(dim(vecs_code_sys2)[1]))

top_n <- 1

CoS_top1 <- pmax(PI_tilde,0)

for(i in 1:dim(CoS_top1)[1]){

temp <- order(CoS_top1[i,],decreasing = T)

CoS_top1[i,temp[-(1:top_n)]] <- 0

CoS_top1[i,] <- CoS_top1[i,]/(sum(CoS_top1[i,]))

}

CoS_thr <- pmax(PI_tilde,thresh)

CoS_thr[which(CoS_thr==thresh, arr.ind=T)] <- 0

for(i in 1:dim(CoS_thr)[1]){

if(sum(CoS_thr[i,])==0) {

CoS_thr[i,] <- CoS_top1[i,]

}

}

temp <- apply(CoS_thr,1,sum)

temp[temp==0] <- 1

CoS_thr <- CoS_thr/temp

}

if (thresh=="cv") {

lambda_set <- seq(0.1,20,0.1)

cv_sum <- NULL

lambda_thr_set <- NULL

k_fold <- dim(vecs_code_sys1_aligned)[2]

cv_dim <- dim(vecs_code_sys1_aligned)[2]/k_fold

# Randomly shuffle by column

new_order <- sample(1:dim(vecs_code_sys2)[2])

vecs_code_sys2_s <- vecs_code_sys2[,new_order]

vecs_code_sys1_aligned_s <- vecs_code_sys1_aligned[,new_order]

for (lambda_i in 1:length(lambda_set)){

lambda <- lambda_set[lambda_i]

PI_tilde_temp <- vecs_code_sys1_aligned%*%t(vecs_code_sys2)%*%solve(vecs_code_sys2%*%t(vecs_code_sys2)+lambda*diag(dim(vecs_code_sys2)[1]))

thr_set_temp <- seq(0,max(PI_tilde_temp),0.01)

for (thr_i in 1:length(thr_set_temp)){

thr <- thr_set_temp[thr_i]

lambda_thr_temp <- c(lambda,thr)

lambda_thr_set <- rbind(lambda_thr_set,lambda_thr_temp)

cv_sum_temp <- 0

for (cv in 1:k_fold){

test_start <- (cv-1)*cv_dim+1

test_end <- cv*cv_dim

vecs_code_sys1_aligned_train <- vecs_code_sys1_aligned_s[,-(test_start:test_end)]

vecs_code_sys2_train <- vecs_code_sys2_s[,-(test_start:test_end)]

vecs_code_sys1_aligned_test <- vecs_code_sys1_aligned_s[,test_start:test_end]

vecs_code_sys2_test <- vecs_code_sys2_s[,test_start:test_end]

vecs_code_sys1_aligned_train <- vecs_code_sys1_aligned_train/sqrt(apply(vecs_code_sys1_aligned_train^2,1,sum))

vecs_code_sys2_train <- vecs_code_sys2_train/sqrt(apply(vecs_code_sys2_train^2,1,sum))

PI_tilde_temp <- vecs_code_sys1_aligned_train%*%t(vecs_code_sys2_train)%*%solve(vecs_code_sys2_train%*%t(vecs_code_sys2_train)+lambda*diag(dim(vecs_code_sys2_train)[1]))

top_n=1

PI_tilde_top_temp=pmax(PI_tilde_temp,0)

for(i in 1:dim(PI_tilde_top_temp)[1]){

temp=order(PI_tilde_top_temp[i,],decreasing = T)

PI_tilde_top_temp[i,temp[-(1:top_n)]]=0

PI_tilde_top_temp[i,]=PI_tilde_top_temp[i,]/(sum(PI_tilde_top_temp[i,]))

}

PI_tilde_top1_temp <- PI_tilde_top_temp

PI_tilde_thr_temp=pmax(PI_tilde_temp,thr)

PI_tilde_thr_temp[which(PI_tilde_thr_temp==thr,arr.ind=T)]=0

for(i in 1:dim(PI_tilde_thr_temp)[1]){

if(sum(PI_tilde_thr_temp[i,])==0)

PI_tilde_thr_temp[i,]=PI_tilde_top1_temp[i,]

}

temp=apply(PI_tilde_thr_temp,1,sum)

temp[temp==0]=1

PI_tilde_thr_temp=PI_tilde_thr_temp/temp

cv_sum_temp <- cv_sum_temp + (norm(vecs_code_sys1_aligned_test-PI_tilde_thr_temp%*%vecs_code_sys2_test,type="F"))^2

}

cv_sum <- c(cv_sum,cv_sum_temp)

}

}

lambda_thr_set <- as.data.frame(lambda_thr_set)

colnames(lambda_thr_set) <- c("lambda","thr")

lambda_thr_best <- lambda_thr_set[which.min(cv_sum),]

lambda_best <- lambda_thr_best$lambda

thr_best$thr

PI_tilde <- vecs_code_sys1_aligned%*%t(vecs_code_sys2)%*%solve(vecs_code_sys2%*%t(vecs_code_sys2)+lambda_best*diag(dim(vecs_code_sys2)[1]))

top_n <- 1

CoS_top1 <- pmax(PI_tilde,0)

for(i in 1:dim(CoS_top1)[1]){

temp <- order(CoS_top1[i,],decreasing = T)

CoS_top1[i,temp[-(1:top_n)]] <- 0

CoS_top1[i,] <- CoS_top1[i,]/(sum(CoS_top1[i,]))

}

CoS_thr <- pmax(PI_tilde,thresh)

CoS_thr[which(CoS_thr==thresh, arr.ind=T)] <- 0

for(i in 1:dim(CoS_thr)[1]){

if(sum(CoS_thr[i,])==0) {

CoS_thr[i,] <- CoS_top1[i,]

}

}

temp <- apply(CoS_thr,1,sum)

temp[temp==0] <- 1

CoS_thr <- CoS_thr/temp

}

return(list(similarity=PI_tilde, mapping_mat=CoS_thr))

}

if (distance=="RS" & !is.null(frequency) & mapping=="top-K" & is.numeric(lambda)) {

PI_tilde <- vecs_code_sys1_aligned%*%t(vecs_code_sys2)%*%solve(vecs_code_sys2%*%t(vecs_code_sys2)+lambda*diag(dim(vecs_code_sys2)[1]))

Gamma_tilde <- PI_tilde

col_freq <- frequency$freq_sys2[match(row.names(vecs_code_sys2),frequency$CId)]

col_wt <- col_freq/sum(col_freq)

A <- matrix(c(t(col_wt)%*%col_wt,

dim(vecs_code_sys2)[1]*mean(col_wt),

dim(vecs_code_sys2)[1]*mean(col_wt),

dim(vecs_code_sys2)[1]),

nrow=2)

row_freq <- frequency$freq_sys1[match(row.names(vecs_code_sys1_aligned),frequency$CId)]

row_wt <- row_freq/sum(row_freq)

for (i in 1:dim(PI_tilde)[1]){

Gamma_tilde[i,] <- PI_tilde[i,] + cbind(col_wt,rep(1,dim(vecs_code_sys2)[1]))%*%solve(A)%*%as.matrix(c(row_wt[i]-t(as.vector(PI_tilde[i,]))%*%col_wt,1-t(as.vector(PI_tilde[i,]))%*%rep(1,length(col_wt))))

}

PI_tilde <- Gamma_tilde

top_n <- K

CoS_top <- pmax(PI_tilde,0)

for(i in 1:dim(CoS_top)[1]){

temp <- order(CoS_top[i,],decreasing = T)

CoS_top[i,temp[-(1:top_n)]] <- 0

CoS_top[i,] <- CoS_top[i,]/(sum(CoS_top[i,]))

}

return(list(similarity=PI_tilde, mapping_mat=CoS_top))

}

if (distance=="RS" & !is.null(frequency) & mapping=="top-K" & lambda=="cv") {

lambda_set <- seq(0,20,0.1) # candidate set for lambda; could be changed

cv_sum <- rep(NA,length(lambda_set))

k_fold <- dim(vecs_code_sys1_aligned)[2]

cv_dim <- dim(vecs_code_sys1_aligned)[2]/k_fold

# Randomly shuffle by column

new_order <- sample(1:dim(vecs_code_sys2)[2])

vecs_code_sys2_s <- vecs_code_sys2[,new_order]

vecs_code_sys1_aligned_s <- vecs_code_sys1_aligned[,new_order]

for (lambda_i in 1:length(lambda_set)){

lambda <- lambda_set[lambda_i]

cv_sum_temp <- 0

for (cv in 1:k_fold){

test_start <- (cv-1)*cv_dim+1

test_end <- cv*cv_dim

vecs_code_sys1_aligned_train <- vecs_code_sys1_aligned_s[,-(test_start:test_end)]

vecs_code_sys2_train <- vecs_code_sys2_s[,-(test_start:test_end)]

vecs_code_sys1_aligned_test <- vecs_code_sys1_aligned_s[,test_start:test_end]

vecs_code_sys2_test <- vecs_code_sys2_s[,test_start:test_end]

vecs_code_sys1_aligned_train <- vecs_code_sys1_aligned_train/sqrt(apply(vecs_code_sys1_aligned_train^2,1,sum))

vecs_code_sys2_train <- vecs_code_sys2_train/sqrt(apply(vecs_code_sys2_train^2,1,sum))

PI_tilde_temp <- vecs_code_sys1_aligned_train%*%t(vecs_code_sys2_train)%*%solve(vecs_code_sys2_train%*%t(vecs_code_sys2_train)+lambda*diag(dim(vecs_code_sys2_train)[1]))

Gamma_tilde <- PI_tilde_temp

col_freq <- frequency$freq_sys2[match(row.names(vecs_code_sys2_train),frequency$CId)]

col_wt <- col_freq/sum(col_freq)

A <- matrix(c(t(col_wt)%*%col_wt,

dim(vecs_code_sys2)[1]*mean(col_wt),

dim(vecs_code_sys2)[1]*mean(col_wt),

dim(vecs_code_sys2)[1]),

nrow=2)

row_freq <- frequency$freq_sys1[match(row.names(vecs_code_sys1_aligned_train),frequency$CId)]

row_wt <- row_freq/sum(row_freq)

for (i in 1:dim(PI_tilde_temp)[1]){

Gamma_tilde[i,] <- PI_tilde_temp[i,] + cbind(col_wt,rep(1,dim(vecs_code_sys2_train)[1]))%*%solve(A)%*%as.matrix(c(row_wt[i]-t(as.vector(PI_tilde_temp[i,]))%*%col_wt,1-t(as.vector(PI_tilde_temp[i,]))%*%rep(1,length(col_wt))))

}

PI_tilde_temp <- Gamma_tilde

top_n <- K

CoS_top <- pmax(PI_tilde_temp,0)

for(i in 1:dim(CoS_top)[1]){

temp <- order(CoS_top[i,],decreasing = T)

CoS_top[i,temp[-(1:top_n)]] <- 0

CoS_top[i,] <- CoS_top[i,]/(sum(CoS_top[i,]))

}

cv_sum_temp <- cv_sum_temp + (norm(vecs_code_sys1_aligned_test-CoS_top%*%vecs_code_sys2_test,type="F"))^2

}

cv_sum[lambda_i] <- cv_sum_temp

}

lambda_best <- lambda_set[which.min(cv_sum)]

PI_tilde <- vecs_code_sys1_aligned%*%t(vecs_code_sys2)%*%solve(vecs_code_sys2%*%t(vecs_code_sys2)+lambda_best*diag(dim(vecs_code_sys2)[1]))

Gamma_tilde <- PI_tilde

col_freq <- frequency$freq_sys2[match(row.names(vecs_code_sys2),frequency$CId)]

col_wt <- col_freq/sum(col_freq)

A <- matrix(c(t(col_wt)%*%col_wt,

dim(vecs_code_sys2)[1]*mean(col_wt),

dim(vecs_code_sys2)[1]*mean(col_wt),

dim(vecs_code_sys2)[1]),

nrow=2)

row_freq <- frequency$freq_sys1[match(row.names(vecs_code_sys1_aligned),frequency$CId)]

row_wt <- row_freq/sum(row_freq)

for (i in 1:dim(PI_tilde)[1]){

Gamma_tilde[i,] <- PI_tilde[i,] + cbind(col_wt,rep(1,dim(vecs_code_sys2)[1]))%*%solve(A)%*%as.matrix(c(row_wt[i]-t(as.vector(PI_tilde_temp[i,]))%*%col_wt,1-t(as.vector(PI_tilde_temp[i,]))%*%rep(1,length(col_wt))))

}

PI_tilde <- Gamma_tilde

top_n <- K

CoS_top <- pmax(PI_tilde,0)

for(i in 1:dim(CoS_top)[1]){

temp <- order(CoS_top[i,],decreasing = T)

CoS_top[i,temp[-(1:top_n)]] <- 0

CoS_top[i,] <- CoS_top[i,]/(sum(CoS_top[i,]))

}

return(list(similarity=PI_tilde, mapping_mat=CoS_top))

}

if (distance=="RS" & !is.null(frequency) & mapping=="thresh" & is.numeric(lambda)) {

PI_tilde <- vecs_code_sys1_aligned%*%t(vecs_code_sys2)%*%solve(vecs_code_sys2%*%t(vecs_code_sys2)+lambda*diag(dim(vecs_code_sys2)[1]))

Gamma_tilde <- PI_tilde

col_freq <- frequency$freq_sys2[match(row.names(vecs_code_sys2),frequency$CId)]

col_wt <- col_freq/sum(col_freq)

A <- matrix(c(t(col_wt)%*%col_wt,

dim(vecs_code_sys2)[1]*mean(col_wt),

dim(vecs_code_sys2)[1]*mean(col_wt),

dim(vecs_code_sys2)[1]),

nrow=2)

row_freq <- frequency$freq_sys1[match(row.names(vecs_code_sys1_aligned),frequency$CId)]

row_wt <- row_freq/sum(row_freq)

for (i in 1:dim(PI_tilde)[1]){

Gamma_tilde[i,] <- PI_tilde[i,] + cbind(col_wt,rep(1,dim(vecs_code_sys2)[1]))%*%solve(A)%*%as.matrix(c(row_wt[i]-t(as.vector(PI_tilde[i,]))%*%col_wt,1-t(as.vector(PI_tilde[i,]))%*%rep(1,length(col_wt))))

}

PI_tilde <- Gamma_tilde

if (thresh!="cv" & is.numeric(thresh)) {

top_n <- 1

CoS_top1 <- pmax(PI_tilde,0)

for(i in 1:dim(CoS_top1)[1]){

temp <- order(CoS_top1[i,],decreasing = T)

CoS_top1[i,temp[-(1:top_n)]] <- 0

CoS_top1[i,] <- CoS_top1[i,]/(sum(CoS_top1[i,]))

}

CoS_thr <- pmax(PI_tilde,thresh)

CoS_thr[which(CoS_thr==thresh, arr.ind=T)] <- 0

for(i in 1:dim(CoS_thr)[1]){

if(sum(CoS_thr[i,])==0) {

CoS_thr[i,] <- CoS_top1[i,]

}

}

temp <- apply(CoS_thr,1,sum)

temp[temp==0] <- 1

CoS_thr <- CoS_thr/temp

}

if (thresh=="cv") {

#### fold number = dimension of embedding vectors ####

thr_set <- seq(0,max(PI_tilde),0.01)

cv_sum <- rep(NA,length(thr_set))

k_fold <- dim(vecs_code_sys1_aligned)[2]

cv_dim <- dim(vecs_code_sys1_aligned)[2]/k_fold

# Randomly shuffle by column

new_order <- sample(1:dim(vecs_code_sys1_aligned)[2])

vecs_code_sys2_s <- vecs_code_sys2[,new_order]

vecs_code_sys1_aligned_s <- vecs_code_sys1_aligned[,new_order]

for (thr_i in 1:length(thr_set)){

thr <- thr_set[thr_i]

cv_sum_temp <- 0

for (cv in 1:k_fold){

test_start <- (cv-1)*cv_dim+1

test_end <- cv*cv_dim

vecs_code_sys1_aligned_train <- vecs_code_sys1_aligned_s[,-(test_start:test_end)]

vecs_code_sys2_train <- vecs_code_sys2_s[,-(test_start:test_end)]

vecs_code_sys1_aligned_test <- vecs_code_sys1_aligned_s[,test_start:test_end]

vecs_code_sys2_test <- vecs_code_sys2_s[,test_start:test_end]

vecs_code_sys1_aligned_train <- vecs_code_sys1_aligned_train/sqrt(apply(vecs_code_sys1_aligned_train^2,1,sum))

vecs_code_sys2_train <- vecs_code_sys2_train/sqrt(apply(vecs_code_sys2_train^2,1,sum))

PI_tilde_temp <- vecs_code_sys1_aligned_train%*%t(vecs_code_sys2_train)%*%solve(vecs_code_sys2_train%*%t(vecs_code_sys2_train)+lambda*diag(dim(vecs_code_sys2_train)[1]))

Gamma_tilde <- PI_tilde_temp

col_freq <- frequency$freq_sys2[match(row.names(vecs_code_sys2_train),frequency$CId)]

col_wt <- col_freq/sum(col_freq)

A <- matrix(c(t(col_wt)%*%col_wt,

dim(vecs_code_sys2)[1]*mean(col_wt),

dim(vecs_code_sys2)[1]*mean(col_wt),

dim(vecs_code_sys2)[1]),

nrow=2)

row_freq <- frequency$freq_sys1[match(row.names(vecs_code_sys1_aligned_train),frequency$CId)]

row_wt <- row_freq/sum(row_freq)

for (i in 1:dim(PI_tilde_temp)[1]){

Gamma_tilde[i,] <- PI_tilde_temp[i,] + cbind(col_wt,rep(1,dim(vecs_code_sys2)[1]))%*%solve(A)%*%as.matrix(c(row_wt[i]-t(as.vector(PI_tilde_temp[i,]))%*%col_wt,1-t(as.vector(PI_tilde_temp[i,]))%*%rep(1,length(col_wt))))

}

PI_tilde_temp <- Gamma_tilde

top_n <- 1

CoS_top_temp <- pmax(PI_tilde_temp,0)

for(i in 1:dim(CoS_top_temp)[1]){

temp=order(CoS_top_temp[i,],decreasing=T)

CoS_top_temp[i,temp[-(1:top_n)]] <- 0

CoS_top_temp[i,] <- CoS_top_temp[i,]/(sum(CoS_top_temp[i,]))

}

CoS_top1_temp <- CoS_top_temp

CoS_thr_temp <- pmax(PI_tilde_temp,thr)

CoS_thr_temp[which(CoS_thr_temp==thr,arr.ind=T)] <- 0

for(i in 1:dim(CoS_thr_temp)[1]){

if(sum(CoS_thr_temp[i,])==0) {

CoS_thr_temp[i,] <- CoS_top1_temp[i,]

}

}

temp <- apply(CoS_thr_temp,1,sum)

temp[temp==0] <- 1

CoS_thr_temp <- CoS_thr_temp/temp

cv_sum_temp <- cv_sum_temp + (norm(vecs_code_sys1_aligned_test-CoS_thr_temp%*%vecs_code_sys2_test,type="F"))^2

}

cv_sum[thr_i] <- cv_sum_temp

}

thresh <- thr_set[which.min(cv_sum)]

CoS_thr <- pmax(PI_tilde,thresh)

CoS_thr[which(CoS_thr==thresh, arr.ind=T)] <- 0

for(i in 1:dim(CoS_thr)[1]){

if(sum(CoS_thr[i,])==0) {

CoS_thr[i,] <- CoS_top1[i,]

}

}

temp <- apply(CoS_thr,1,sum)

temp[temp==0] <- 1

CoS_thr <- CoS_thr/temp

}

return(list(similarity=PI_tilde, mapping_mat=CoS_thr))

}

if (distance=="RS" & !is.null(frequency) & mapping=="thresh" & lambda=="cv") {

if (thresh!="cv" & is.numeric(thresh)) {

lambda_set <- seq(0,20,0.1) # candidate set for lambda; could be changed

cv_sum <- rep(NA,length(lambda_set))

k_fold <- dim(vecs_code_sys1_aligned)[2]

cv_dim <- dim(vecs_code_sys1_aligned)[2]/k_fold

# Randomly shuffle by column

new_order <- sample(1:dim(vecs_code_sys2)[2])

vecs_code_sys2_s <- vecs_code_sys2[,new_order]

vecs_code_sys1_aligned_s <- vecs_code_sys1_aligned[,new_order]

for (lambda_i in 1:length(lambda_set)){

lambda <- lambda_set[lambda_i]

cv_sum_temp <- 0

for (cv in 1:k_fold){

test_start <- (cv-1)*cv_dim+1

test_end <- cv*cv_dim

vecs_code_sys1_aligned_train <- vecs_code_sys1_aligned_s[,-(test_start:test_end)]

vecs_code_sys2_train <- vecs_code_sys2_s[,-(test_start:test_end)]

vecs_code_sys1_aligned_test <- vecs_code_sys1_aligned_s[,test_start:test_end]

vecs_code_sys2_test <- vecs_code_sys2_s[,test_start:test_end]

vecs_code_sys1_aligned_train <- vecs_code_sys1_aligned_train/sqrt(apply(vecs_code_sys1_aligned_train^2,1,sum))

vecs_code_sys2_train <- vecs_code_sys2_train/sqrt(apply(vecs_code_sys2_train^2,1,sum))

PI_tilde_temp <- SEV_kpwa_selected_train%*%t(vecs_code_sys2_train)%*%solve(vecs_code_sys2_train%*%t(vecs_code_sys2_train)+lambda*diag(dim(vecs_code_sys2_train)[1]))

Gamma_tilde <- PI_tilde_temp

col_freq <- frequency$freq_sys2[match(row.names(vecs_code_sys2_train),frequency$CId)]

col_wt <- col_freq/sum(col_freq)

A <- matrix(c(t(col_wt)%*%col_wt,

dim(vecs_code_sys2)[1]*mean(col_wt),

dim(vecs_code_sys2)[1]*mean(col_wt),

dim(vecs_code_sys2)[1]),

nrow=2)

row_freq <- frequency$freq_sys1[match(row.names(vecs_code_sys1_aligned_train),frequency$CId)]

row_wt <- row_freq/sum(row_freq)

for (i in 1:dim(PI_tilde_temp)[1]){

Gamma_tilde[i,] <- PI_tilde_temp[i,] + cbind(col_wt,rep(1,dim(vecs_code_sys2_train)[1]))%*%solve(A)%*%as.matrix(c(row_wt[i]-t(as.vector(PI_tilde_temp[i,]))%*%col_wt,1-t(as.vector(PI_tilde_temp[i,]))%*%rep(1,length(col_wt))))

}

PI_tilde_temp <- Gamma_tilde

top_n <- 1

CoS_top1_temp <- pmax(PI_tilde_temp,0)

for(i in 1:dim(CoS_top1_temp)[1]){

temp <- order(CoS_top1_temp[i,],decreasing = T)

CoS_top1_temp[i,temp[-(1:top_n)]] <- 0

CoS_top1_temp[i,] <- CoS_top1_temp[i,]/(sum(CoS_top1_temp[i,]))

}

CoS_thr_temp <- pmax(PI_tilde_temp,thresh)

CoS_thr_temp[which(CoS_thr_temp==thresh, arr.ind=T)] <- 0

for(i in 1:dim(CoS_thr_temp)[1]){

if(sum(CoS_thr_temp[i,])==0) {

CoS_thr_temp[i,] <- CoS_top1_temp[i,]

}

}

temp <- apply(CoS_thr_temp,1,sum)

temp[temp==0] <- 1

CoS_thr_temp <- CoS_thr_temp/temp

cv_sum_temp <- cv_sum_temp + (norm(vecs_code_sys1_aligned_test-CoS_thr_temp%*%vecs_code_sys2_test,type="F"))^2

}

cv_sum[lambda_i] <- cv_sum_temp

}

lambda_best <- lambda_set[which.min(cv_sum)]

PI_tilde <- vecs_code_sys1_aligned%*%t(vecs_code_sys2)%*%solve(vecs_code_sys2%*%t(vecs_code_sys2)+lambda_best*diag(dim(vecs_code_sys2)[1]))

Gamma_tilde <- PI_tilde

col_freq <- frequency$freq_sys2[match(row.names(vecs_code_sys2),frequency$CId)]

col_wt <- col_freq/sum(col_freq)

A <- matrix(c(t(col_wt)%*%col_wt,

dim(vecs_code_sys2)[1]*mean(col_wt),

dim(vecs_code_sys2)[1]*mean(col_wt),

dim(vecs_code_sys2)[1]),

nrow=2)

row_freq <- frequency$freq_sys1[match(row.names(vecs_code_sys1_aligned),frequency$CId)]

row_wt <- row_freq/sum(row_freq)

for (i in 1:dim(PI_tilde)[1]){

Gamma_tilde[i,] <- PI_tilde[i,] + cbind(col_wt,rep(1,dim(vecs_code_sys2)[1]))%*%solve(A)%*%as.matrix(c(row_wt[i]-t(as.vector(PI_tilde[i,]))%*%col_wt,1-t(as.vector(PI_tilde[i,]))%*%rep(1,length(col_wt))))

}

PI_tilde <- Gamma_tilde

top_n <- 1

CoS_top1 <- pmax(PI_tilde,0)

for(i in 1:dim(CoS_top1)[1]){

temp <- order(CoS_top1[i,],decreasing = T)

CoS_top1[i,temp[-(1:top_n)]] <- 0

CoS_top1[i,] <- CoS_top1[i,]/(sum(CoS_top1[i,]))

}

CoS_thr <- pmax(PI_tilde,thresh)

CoS_thr[which(CoS_thr==thresh, arr.ind=T)] <- 0

for(i in 1:dim(CoS_thr)[1]){

if(sum(CoS_thr[i,])==0) {

CoS_thr[i,] <- CoS_top1[i,]

}

}

temp <- apply(CoS_thr,1,sum)

temp[temp==0] <- 1

CoS_thr <- CoS_thr/temp

}

if (thresh=="cv") {

lambda_set <- seq(0.1,20,0.1)

cv_sum <- NULL

lambda_thr_set <- NULL

k_fold <- dim(vecs_code_sys1_aligned)[2]

cv_dim <- dim(vecs_code_sys1_aligned)[2]/k_fold

# Randomly shuffle by column

new_order <- sample(1:dim(vecs_code_sys2)[2])

vecs_code_sys2_s <- vecs_code_sys2[,new_order]

vecs_code_sys1_aligned_s <- vecs_code_sys1_aligned[,new_order]

for (lambda_i in 1:length(lambda_set)){

lambda <- lambda_set[lambda_i]

PI_tilde_temp <- vecs_code_sys1_aligned%*%t(vecs_code_sys2)%*%solve(vecs_code_sys2%*%t(vecs_code_sys2)+lambda*diag(dim(vecs_code_sys2)[1]))

Gamma_tilde <- PI_tilde_temp

col_freq <- frequency$freq_sys2[match(row.names(vecs_code_sys2),frequency$CId)]

col_wt <- col_freq/sum(col_freq)

A <- matrix(c(t(col_wt)%*%col_wt,

dim(vecs_code_sys2)[1]*mean(col_wt),

dim(vecs_code_sys2)[1]*mean(col_wt),

dim(vecs_code_sys2)[1]),

nrow=2)

row_freq <- frequency$freq_sys1[match(row.names(vecs_code_sys1_aligned),frequency$CId)]

row_wt <- row_freq/sum(row_freq)

for (i in 1:dim(PI_tilde_temp)[1]){

Gamma_tilde[i,] <- PI_tilde_temp[i,] + cbind(col_wt,rep(1,dim(vecs_code_sys2)[1]))%*%solve(A)%*%as.matrix(c(row_wt[i]-t(as.vector(PI_tilde_temp[i,]))%*%col_wt,1-t(as.vector(PI_tilde_temp[i,]))%*%rep(1,length(col_wt))))

}

PI_tilde_temp <- Gamma_tilde

thr_set_temp <- seq(0,max(PI_tilde_temp),0.01)

for (thr_i in 1:length(thr_set_temp)){

thr <- thr_set_temp[thr_i]

lambda_thr_temp <- c(lambda,thr)

lambda_thr_set <- rbind(lambda_thr_set,lambda_thr_temp)

cv_sum_temp <- 0

for (cv in 1:k_fold){

test_start <- (cv-1)*cv_dim+1

test_end <- cv*cv_dim

vecs_code_sys1_aligned_train <- vecs_code_sys1_aligned_s[,-(test_start:test_end)]

vecs_code_sys2_train <- vecs_code_sys2_s[,-(test_start:test_end)]

vecs_code_sys1_aligned_test <- vecs_code_sys1_aligned_s[,test_start:test_end]

vecs_code_sys2_test <- vecs_code_sys2_s[,test_start:test_end]

vecs_code_sys1_aligned_train <- vecs_code_sys1_aligned_train/sqrt(apply(vecs_code_sys1_aligned_train^2,1,sum))

vecs_code_sys2_train <- vecs_code_sys2_train/sqrt(apply(vecs_code_sys2_train^2,1,sum))

PI_tilde_temp <- vecs_code_sys1_aligned_train%*%t(vecs_code_sys2_train)%*%solve(vecs_code_sys2_train%*%t(vecs_code_sys2_train)+lambda*diag(dim(vecs_code_sys2_train)[1]))

Gamma_tilde <- PI_tilde_temp

col_freq <- frequency$freq_sys2[match(row.names(vecs_code_sys2_train),frequency$CId)]

col_wt <- col_freq/sum(col_freq)

A <- matrix(c(t(col_wt)%*%col_wt,

dim(vecs_code_sys2)[1]*mean(col_wt),

dim(vecs_code_sys2)[1]*mean(col_wt),

dim(vecs_code_sys2)[1]),

nrow=2)

row_freq <- frequency$freq_sys1[match(row.names(vecs_code_sys1_aligned_train),frequency$CId)]

row_wt <- row_freq/sum(row_freq)

for (i in 1:dim(PI_tilde_temp)[1]){

Gamma_tilde[i,] <- PI_tilde_temp[i,] + cbind(col_wt,rep(1,dim(vecs_code_sys2_train)[1]))%*%solve(A)%*%as.matrix(c(row_wt[i]-t(as.vector(PI_tilde_temp[i,]))%*%col_wt,1-t(as.vector(PI_tilde_temp[i,]))%*%rep(1,length(col_wt))))

}

PI_tilde_temp <- Gamma_tilde

top_n=1

PI_tilde_top_temp=pmax(PI_tilde_temp,0)

for(i in 1:dim(PI_tilde_top_temp)[1]){

temp=order(PI_tilde_top_temp[i,],decreasing = T)

PI_tilde_top_temp[i,temp[-(1:top_n)]]=0

PI_tilde_top_temp[i,]=PI_tilde_top_temp[i,]/(sum(PI_tilde_top_temp[i,]))

}

PI_tilde_top1_temp <- PI_tilde_top_temp

PI_tilde_thr_temp=pmax(PI_tilde_temp,thr)

PI_tilde_thr_temp[which(PI_tilde_thr_temp==thr,arr.ind=T)]=0

for(i in 1:dim(PI_tilde_thr_temp)[1]){

if(sum(PI_tilde_thr_temp[i,])==0)

PI_tilde_thr_temp[i,]=PI_tilde_top1_temp[i,]

}

temp=apply(PI_tilde_thr_temp,1,sum)

temp[temp==0]=1

PI_tilde_thr_temp=PI_tilde_thr_temp/temp

cv_sum_temp <- cv_sum_temp + (norm(vecs_code_sys1_aligned_test-PI_tilde_thr_temp%*%vecs_code_sys2_test,type="F"))^2

}

cv_sum <- c(cv_sum,cv_sum_temp)

}

}

lambda_thr_set <- as.data.frame(lambda_thr_set)

colnames(lambda_thr_set) <- c("lambda","thr")

lambda_thr_best <- lambda_thr_set[which.min(cv_sum),]

lambda_best <- lambda_thr_best$lambda

thr_best <- lambda_thr_best$thr

PI_tilde <- vecs_code_sys1_aligned%*%t(vecs_code_sys2)%*%solve(vecs_code_sys2%*%t(vecs_code_sys2)+lambda_best*diag(dim(vecs_code_sys2)[1]))

top_n <- 1

CoS_top1 <- pmax(PI_tilde,0)

for(i in 1:dim(CoS_top1)[1]){

temp <- order(CoS_top1[i,],decreasing = T)

CoS_top1[i,temp[-(1:top_n)]] <- 0

CoS_top1[i,] <- CoS_top1[i,]/(sum(CoS_top1[i,]))

}

CoS_thr <- pmax(PI_tilde,thr_best)

CoS_thr[which(CoS_thr==thr_best, arr.ind=T)] <- 0

for(i in 1:dim(CoS_thr)[1]){

if(sum(CoS_thr[i,])==0) {

CoS_thr[i,] <- CoS_top1[i,]

}

}

temp <- apply(CoS_thr,1,sum)

temp[temp==0] <- 1

CoS_thr <- CoS_thr/temp

}

return(list(similarity=PI_tilde, mapping_mat=CoS_thr))

}

}

**R code for the validation function**

validation <- function (code_data_sys1, code_data_sys2, mapping_mat, V, confidence) {

mapping_mat <- mapping_mat[,colnames(code_data_sys2)]

mapping_mat <- mapping_mat[colnames(code_data_sys1),]

code_data_sys2_mapped <- code_data_sys2%*%t(mapping_mat)

data_logistic <- data.frame(Y=c(rep(0,nrow(code_data_sys1)),

rep(1,nrow(code_data_sys2_mapped))),

rbind(code_data_sys1,code_data_sys2_mapped))

sample_num <- sample(1:nrow(data_logistic))

for (v_num in 1:V) {

assign(paste0("train_", v_num), data_logistic[-sample_num[((nrow(data_logistic)/V)*(v_num-1)+1):((nrow(data_logistic)/V)*v_num)],])

assign(paste0("test_", v_num), data_logistic[sample_num[((nrow(data_logistic)/V)*(v_num-1)+1):((nrow(data_logistic)/V)*v_num)],])

}

for (v_num in 1:V) {

train_temp <- get(paste0("train_", v_num))

test_temp <- get(paste0("test_", v_num))

assign(paste0("model_", v_num), glm(Y~., family="binomial", data=train_temp))

assign(paste0("predicted_", v_num),predict(get(paste0("model_", v_num)), test_temp, type="response"))

}

predicted_c <- NULL

for (v_num in 1:V) {

predicted_c <- c(predicted_c,get(paste0("predicted_", v_num)))

}

test_Y_c <- NULL

for (v_num in 1:V) {

test_Y_c <- c(test_Y_c,get(paste0("test_", v_num))$Y)

}

nrow_c <- list(1:(nrow(data_logistic)/V))

for (v_num in 2:V) {

list_temp <- list(((nrow(data_logistic)/V)*(v_num-1)+1):((nrow(data_logistic)/V)*v_num))

nrow_c <- c(nrow_c,list_temp)

}

cvAUC_result <- ci.cvAUC(predicted_c, test_Y_c,

label.ordering = NULL,

folds = nrow_c,

confidence = 0.95)

colnames_com <- unique(c(colnames(code_data_sys1),colnames(code_data_sys2)))

code_data_sys1_original <- code_data_sys1

code_data_sys1 <- matrix(0,nrow=nrow(code_data_sys1),ncol=length(colnames_com))

code_data_sys2_mapped <- matrix(0,nrow=nrow(code_data_sys2),ncol=length(colnames_com))

for (j in 1:length(colnames_com)) {

if (colnames_com[j]%in%colnames(code_data_sys1_original)) {

code_data_sys1[,j] <- code_data_sys1_original[,colnames(code_data_sys1_original)==(colnames_com[j])]

}

if (colnames_com[j]%in%colnames(code_data_sys2)) {

code_data_sys2_mapped[,j] <- code_data_sys2[,colnames(code_data_sys2)==(colnames_com[j])]

}

}

data_logistic <- data.frame(Y=c(rep(0,nrow(code_data_sys1)),

rep(1,nrow(code_data_sys2_mapped))),

rbind(code_data_sys1,code_data_sys2_mapped))

sample_num <- sample(1:nrow(data_logistic))

for (v_num in 1:V) {

assign(paste0("train_", v_num), data_logistic[-sample_num[((nrow(data_logistic)/V)*(v_num-1)+1):((nrow(data_logistic)/V)*v_num)],])

assign(paste0("test_", v_num), data_logistic[sample_num[((nrow(data_logistic)/V)*(v_num-1)+1):((nrow(data_logistic)/V)*v_num)],])

}

for (v_num in 1:V) {

train_temp <- get(paste0("train_", v_num))

test_temp <- get(paste0("test_", v_num))

assign(paste0("model_", v_num), glm(Y~., family="binomial", data=train_temp))

assign(paste0("predicted_", v_num),predict(get(paste0("model_", v_num)), test_temp, type="response"))

}

predicted_c <- NULL

for (v_num in 1:V) {

predicted_c <- c(predicted_c,get(paste0("predicted_", v_num)))

}

test_Y_c <- NULL

for (v_num in 1:V) {

test_Y_c <- c(test_Y_c,get(paste0("test_", v_num))$Y)

}

nrow_c <- list(1:(nrow(data_logistic)/V))

for (v_num in 2:V) {

list_temp <- list(((nrow(data_logistic)/V)*(v_num-1)+1):((nrow(data_logistic)/V)*v_num))

nrow_c <- c(nrow_c,list_temp)

}

cvAUC_result_before <- ci.cvAUC(predicted_c, test_Y_c, label.ordering = NULL,

folds = nrow_c, confidence = 0.95)

return(list(cvAUC_before=cvAUC_result_before$cvAUC,

ci_before=cvAUC_result_before$ci,

cvAUC_after=cvAUC_result$cvAUC,

ci_after=cvAUC_result$ci))

}
